# Helquat dyes targeting G-quadruplexes as a new class of anti-HIV-1 inhibitors

**DOI:** 10.1038/s41598-023-33263-3

**Published:** 2023-04-13

**Authors:** Marcela Pávová, Paul Eduardo Reyes-Gutiérrez, Jaroslav Kozák, Juraj Dobiaš, Yevgen Yurenko, Martin Lepšík, Filip Teplý, Jan Weber

**Affiliations:** Institute of Organic Chemistry and Biochemistry of the Czech Academy of Sciences, Prague, 160 00 Czech Republic

**Keywords:** Virology, Nucleic acids

## Abstract

The secondary structure of nucleic acids containing quartets of guanines, termed G-quadruplexes, is known to regulate the transcription of many genes. Several G-quadruplexes can be formed in the HIV-1 long terminal repeat promoter region and their stabilization results in the inhibition of HIV-1 replication. Here, we identified helquat-based compounds as a new class of anti-HIV-1 inhibitors that inhibit HIV-1 replication at the stage of reverse transcription and provirus expression. Using Taq polymerase stop and FRET melting assays, we have demonstrated their ability to stabilize G-quadruplexes in the HIV-1 long-terminal repeat sequence. Moreover, these compounds were not binding to the general G-rich region, but rather to G-quadruplex-forming regions. Finally, docking and molecular dynamics calculations indicate that the structure of the helquat core greatly affects the binding mode to the individual G-quadruplexes. Our findings can provide useful information for the further rational design of inhibitors targeting G-quadruplexes in HIV-1.

## Introduction

It is well accepted that G-quadruplexes (G4s) play an important regulatory role in the promoters of many cellular genes^1^, oncogenes in particular, and that their misregulation is associated with various human diseases^2–4^. Over the last few years, the presence of G4s in human pathogens^5–7^, and especially in viruses^8^, and their involvement in many key steps of viral life cycles have attracted increasing interest^9–15^ (summarized in extensive reviews^16–19^).

G4s are promising pharmacological targets, because the distinct molecular features of the individual G4s enable selective recognition by small molecules with minimal side effects^20^. This approach is being investigated in anticancer research by targeting G4s located in telomeres or oncogenes^21–24^ and can be adopted in antiviral research, as documented by Perrone et al., who originally demonstrated the anti-HIV activity of the G4 ligand BRACO-19^11^. This finding was followed by others, resulting in the publication of the antiviral properties of other G4-binding ligands^10,25–33^.

**Figure 1:**
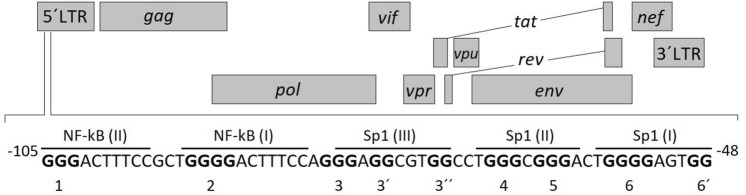
HIV-1 genomic organization and a sequence of the G-rich LTR region spanning nucleotides − 105/− 48. The lines indicate transcription factor binding sites. The G bases involved in the formation of G-quadruplexes are in bold and G-tracts are numbered.

Retroviruses are multifaceted viruses that infect almost all vertebrates and cause severe diseases, including immunodeficiency, neurological disorders, and various types of cancer, representing a major threat across the animal kingdom. The human immunodeficiency virus type 1 (HIV-1), the etiological agent of the acquired immunodeficiency syndrome (AIDS), establishes a persistent infection in cells of the human immune system. Recently, Bohálová et al. analysed all available viral genomes in the NCBI database (11,000 genomes in total) for putative G-quadruplex forming sequences (PQS) and showed that viruses causing persistent infection, such as HIV-1, are enriched for the presence of PQS whereas the viruses causing acute infections only are often reduced in PQS^34^. The presence of G4 structures has been described in both RNA and DNA forms of its genome, with implications throughout the HIV-1 life cycle^35,36^. Perhaps most importantly, the HIV-1 promoter region, specifically the U3 region of the 5′ LTR (long terminal repeat), contains a G-rich sequence of 50 nucleotides upstream from the transcription-starting site and close to the TATA box (Fig. 1). This region readily takes on different G4 configurations within both the RNA virus genome and the single-stranded DNA form during the transcription of the integrated provirus^36^. This region contains several G-patches (labelled as 1–6′ in Fig. 1) that are, based on computational, biophysical and biochemical analyses, able to form three mutually exclusive G4s. Several publications have identified G-patches 3″–6 as a region indispensable for the formation of all relevant G4s^11,36,37^. Moreover, Perrone et al. have observed the formation of an additional G4, exploiting G-patches 4–6′, after treatment with a G4-binding ligand^11^, and Butovskaya et al. have confirmed this G4 as the major G4 structure formed in the HIV-1 LTR^38^. The core of the G4-forming region is composed of G-patches 4 and 5, as documented by two point mutations (G61A and G65A) that completely erase the ability of this region to form any G4^11^.

Despite the considerable success of antiretroviral therapy in the treatment of HIV/AIDS, there is an urgent need for new anti-HIV drugs targeting highly conserved features of HIV-1, which will be difficult for the virus to bypass or compensate for. G4s in the HIV-1 promoter region may represent such a target as documented by their high evolutionary conservation across all HIV-1 subspecies^37,39^. Several studies have linked this region to the effectivity of provirus transcription as documented by the effect of point mutations that disrupt the G4 structures formed in the promoter, or by the binding of G4-stabilizing ligands^11,40,41^. Moreover, mutations in promoter regulatory elements are likely to impact viral fitness, limiting the emergence of resistant strains. Furthermore, the HIV-1 promoter sequence cannot be found in the human genome and the polymorphism of G4 structures indicates that it is possible to achieve a high degree of drug specificity. In addition, the importance of non-B DNA structures in the host genome, including G-quadruplexes, was recently demonstrated for productive and latent HIV-1 integration and the reactivation potential of latent proviruses^42^. Finally, the crucial role of RNA G-quadruplexes at the early steps of HIV-1 life cycle was unravelled when stabilization of HIV G4 structures by G4-binding ligands prevented the initiation of reverse transcription^43^.

**Figure 2:**
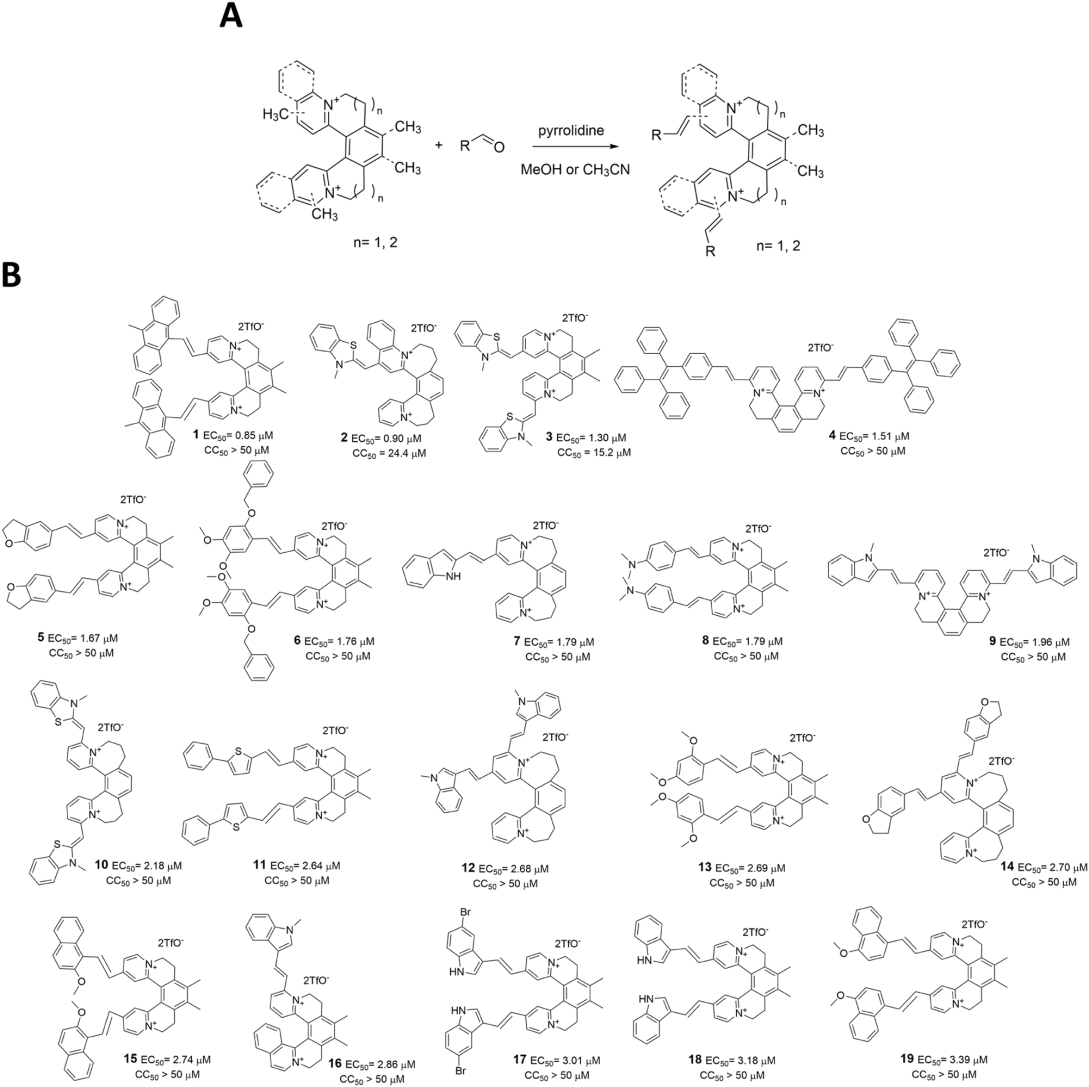
(**A**) Synthesis of substituted helquats using a Knoevenagel condensation. (**B**) Structures and EC_50_ and CC_50_ values of selected helquat compounds in cytopathic effect-based assay using MT-4 cells infected by HIV-1 (NL4-3 strain).

The general features of G4-binding compounds include an electron-rich aromatic core with potential for a flat conformation and an ability to participate in hydrogen bonding^44^. These requirements are perfectly fulfilled by helquats (Fig. 2). Helquat-based compounds can interact with high selectivity for ds-DNA and by changing certain structural features in the main helquat core, this class of compounds could be made selective for ss-DNA over ds-DNA^45^, which makes them attractive for the development of a new class of G4 stabilizers. Here, we synthesized a group of helquat-based compounds capable of interacting with G4-forming sequences leading to their stabilization. These compounds were screened for an inhibitory effect on HIV-1 replication. Selected hits were then evaluated by several virological methods (adsorption and replication assays, a time-of-addition assay) to address the timing of their antiviral action and biophysical methods (FRET and *Taq* polymerase stop assays and light-up experiments) to examine their mode of action. Computational methods were subsequently used to estimate their binding modes to two G4 patches.

## Results

### An CPE-based antiviral assay in HIV-1-infected MT-4 cells was used to determine the EC_50_ of compounds

Based on a facile [2 + 2 + 2]‐cycloisomerization strategy, a robust three‐step synthetic entry into a novel family of helical extended diquats (helquats) was developed^46^. After a straightforward Knoevenagel condensation of the respective helquat parent with different aldehydes (Fig. 2A), more than 500 helquat-based compounds were synthesized and screened for anti-HIV-1 activity in an assay based on the inhibition of the virus-induced cytopathic effect in MT-4 cells, evaluated after 5 days post-infection by an XTT assay. In parallel with the screening, the XTT assay was also used to determine the cytotoxicity of all compounds (Fig. 2B; for more information on the synthesis and characterization of the compounds, see the Supplementary Information).

The compounds were ranked by their EC_50_, and the top 19 compounds that had their EC_50_ lower than 4 µM were selected for this study (Fig. 2). Only two compounds have their EC_50_ values below 1 µM (**1** and **2**). Out of the selected compounds, only two exhibit cytotoxicity to MT-4 cells. Compounds **2** and **3** have CC_50_ values of 24.4 μM and 15.2 μM, respectively. Because the remaining compounds do not exhibit cytotoxicity in the range tested, their CC_50_ values are described as greater than 50 μM.

### Helquats inhibit different stages of the HIV-1 life cycle

It has been reported that compounds with multiple positive charges can unspecifically block viral entry^47^. Because the compounds which we tested are protonated under physiological conditions^48^, their antiviral effect could result from virion damage or reduced virion binding and entry into target cells. To investigate this possibility, we performed adsorption and replication assays.

**Figure 3:**
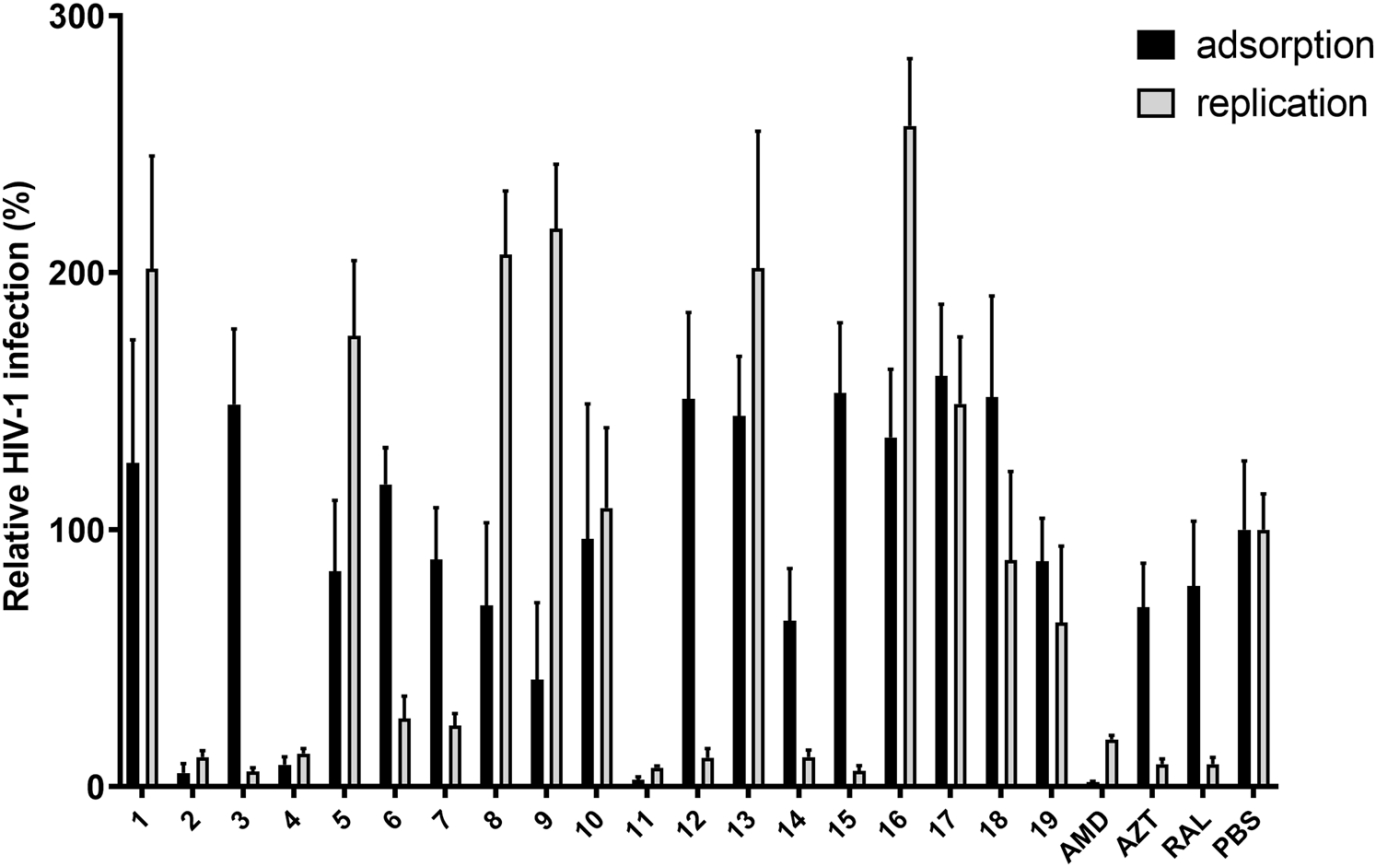
Adsorption and replication assays for individual compounds tested. The levels of HIV-1 infection were normalized against the phosphate-buffered saline (PBS) control in each individual experiment. AMD—AMD3100, AZT—azidothymidine and RAL—raltegravir were used as HIV-1 inhibition controls. Error bars represent standard errors from three independent experiments.

To address virion binding and entry in a competitive setting, we performed an adsorption assay where test compounds and virions were added to MT-4 cells simultaneously and were washed out after 2 h of incubation. In parallel, we performed a replication assay where MT-4 cells were infected with HIV-1 and the tested compounds were added 1 h later. In both cases, the infection was evaluated by measuring reverse transcriptase activity in a cell-free supernatant 5 days after the infection (Fig. 3). As controls, PBS (phosphate-buffered saline) and inhibitors of different HIV-1 life-cycle steps were used: AMD (AMD3100), an inhibitor of gp120 attachment to CXCR4 receptors of the target cell, thus preventing HIV-1 adsorption and entry; AZT (azidothymidine), an inhibitor of reverse transcriptase; and RAL (raltegravir), an integrase inhibitor.

Some compounds (namely **1**, **5**, **10**, **13**, **16**, **17**, **18** and **19**) inhibited neither adsorption nor replication, or their effect was weak, whereas other compounds (**2**, **4**, **8**, **9** and **11**) inhibited adsorption. Compounds compromising viral entry were excluded from further analyses because their effect is probably nonspecific, mainly because their cationic nature. The results of these two experiments led to the selection of compounds **3**, **6**, **7**, **12**, **14** and **15**, which had minimal or no effect on virus adsorption while significantly impairing viral replication.

### Helquats regulate G-quadruplexes in HIV-1 at the level of both RNA and DNA

**Figure 4:**
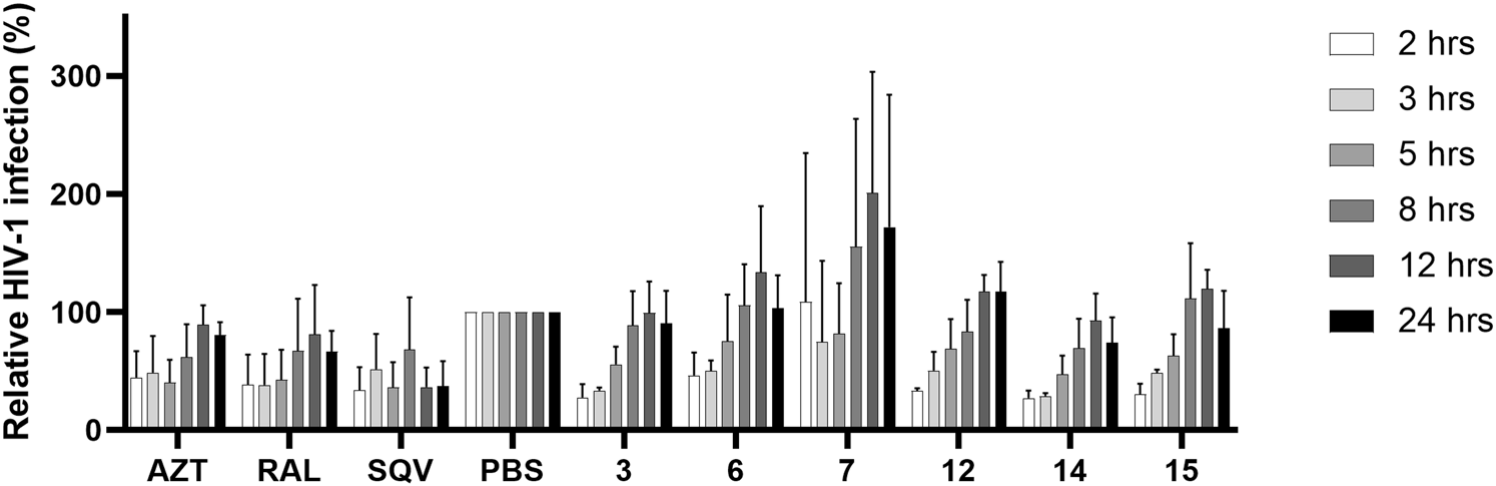
Modified time-of-addition assay. The panel of control compounds includes AZT (azidothymidine, an inhibitor of reverse transcriptase), RAL (raltegravir, an integrase inhibitor), SQV (saquinavir, a protease inhibitor) and PBS (phosphate-buffered saline, control). The levels of HIV-1 infection for each time point were normalized against the respective PBS control. Error bars represent standard errors from three independent experiments.

There are indications that G4s in the HIV-1 promoter might play roles in both RNA and DNA forms. G4s present in the LTR region of HIV-1 genomic RNA could influence reverse transcription. By contrast, G4s in the promoter region of the integrated HIV-1 provirus might regulate the transcription of viral genome and of viral mRNAs and thus the expression of viral proteins and the production of new virions - both the later stages of the HIV-1 life cycle. A modified time-of-addition assay was performed with an emphasis on the later stages of the HIV-1 life cycle, such as reverse transcription, integration and provirus expression. The tested compounds were added at several time points, but the initial time point was 2 h post-infection to eliminate their virucidal/adsorption/entry effects. Several inhibitors of the individual steps of the HIV-1 life cycle were used as controls to resolve the post-entry steps of the HIV-1 life cycle. MT-4 cells were infected with NL4-3 HIV-1 and the infection was allowed to proceed for 1 h. Subsequently, the unbound virions were washed out, and the cells were resuspended in fresh media. The compounds were first added 2 h after the infection. The culture media were harvested 31 h later and the number of released virions was monitored through the activity of reverse transcriptase in the culture media (Fig. 4).

All the compounds presented in Fig. 4, except for **7**, which was excluded from further experiments due to large results variability, were active when added at early time points of the experiment (i.e. two and 3 h post-infection), but only some of them (**12** and **14**) preserved their activity at later time points. In comparison with the control compounds (AZT—inhibitor of reverse transcriptase and RAL—inhibitor of integrase), these results suggest that the effect of **3**, **6** and **15** is mainly manifested when these compounds are administered before or early during reverse transcription; therefore, these compounds are likely to act already at the RNA level. By contrast, the second group of compounds (**12** and **14**) can be added later (up to 8 h), when reverse transcription is mostly completed, indicating that these compounds might influence the G-quadruplexes also at the DNA level.

### Helquats stabilize G-quadruplexes in the HIV-1 LTR promoter

Perrone et al.^11^ have published that the conserved region of the HIV-1 promoter spanning the NF-κB site and Sp1 sites contains G-tracts involved in the formation of mutually exclusive G4 structures. Furthermore, they have shown the stabilization of these G4s by quadruplex-binding ligands, such as TMPyP4 or BRACO-19 and the formation of an additional G4, which is induced by these ligands. The aforementioned experiments have indicated that active compounds might modify the stability of HIV-1 LTR G4s. We have used a construct corresponding to the G-rich part of the core HIV-1 promoter (positions − 105/ − 48 with respect to the transcription initiation site) with FAM and TAMRA moieties placed at their 5′- and 3′-ends, respectively. The stability of the G4s formed in the presence or absence of G4-binding ligands has been assessed by melting-curve experiments monitored based on the increase in FAM-fluorophore fluorescence (a representative examples of FRET melting curves is in Supplement Information Fig. S1).

The results show that all the compounds tested at the concentration of 2 μM stabilize G-quadruplexes in the HIV-1 LTR (0.2 μM concentration), as demonstrated by an increase in melting temperature (T_m_), albeit to a different extent (ΔT_m_ of 10–28 °C, Table 1). This experiment has confirmed that the compounds tested are able to stabilize G-quadruplexes in vitro and indicates that the observed HIV-1 inhibitory effect of the tested compounds might be caused by their stabilization of G-quadruplexes in the HIV-1 LTR region.

**Table 1:** FRET analysis of G-quadruplex melting temperatures in the presence of tested compounds at a concentration of 2 μM.

Compound	T_m_ (°C)	ΔT_m_ (°C)
3	61 ± 0.1	10
6	65 ± 0.1	14
12	79 ± 0.2	28
14	64 ± 0.2	13
15	70 ± 1.0	19
PDS	93 ± 13	42
PBS	51 ± 0.1	0

PDS (pyridostatin)—positive control, PBS (buffer)—negative control. The ΔT_m_ was calculated by subtracting the average T_m_ of PBS (control) from average T_m_ for each compound. The T_m_ results were averaged from three independent experiments, depicted as mean and standard error.

### Helquats bind mostly to the 6 and 6′ G-patches of the HIV-1 promoter

To address specifically the positions in the HIV-1 promoter region that are affected by the binding of G4-stabilizing agents, we performed a *Taq* polymerase stop assay. The wild-type LTR oligonucleotide was extended at the 5′end to include a primer-annealing region and used as a template for a single-cycle *Taq* polymerase reaction. The elongation of the radioactively labelled primer was performed at 45 °C in the presence or absence of the tested compounds and controls. Most of the tested compounds (16 of the 19 compounds originally selected for this study) exhibit some degree of G-quadruplex stabilization at the concentration of 2.5 µM, as manifested by an increase in the intensity of the bands in the individual G-rich patches, even though the extent of the observed effect is different.

**Figure 5:**
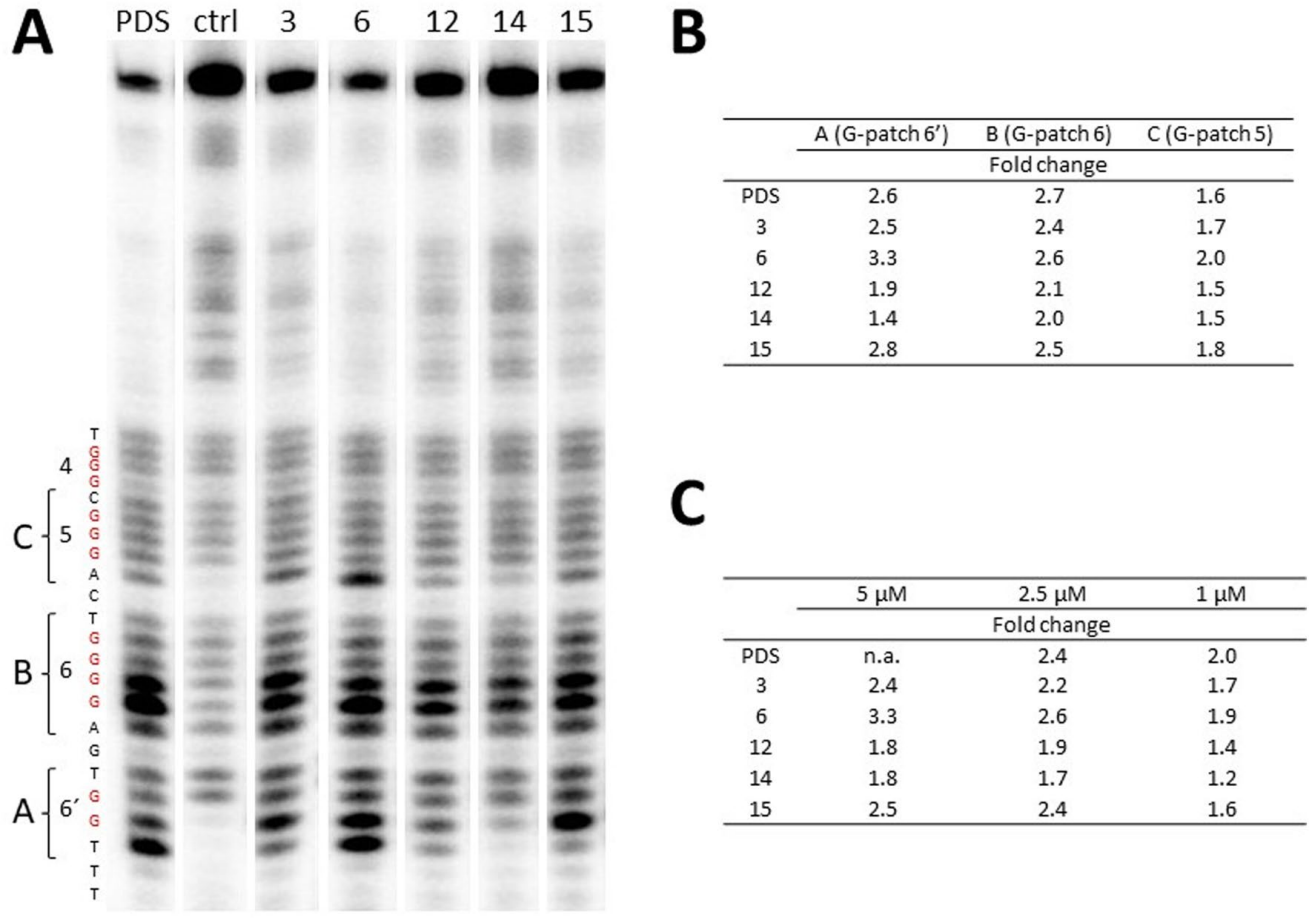
Identification of individual G-tracts stabilized by helquat binding. (**A**) A *Taq* polymerase stop assay in the presence of 2.5 µM of the compounds being tested, with stop regions visible as bands of higher intensity. The sequence and numbered G-tracts (highlighted in red) are indicated on the left. The brackets labelled with letters A, B and C mark the regions selected for quantification. This figure was made by the excision of selected lines from Supplementary Fig. 2C, which shows the entire original gel. (**B**) Quantification of selected regions, normalized against the negative control (ctrl). The values represent a fold change calculated as ratio between pixel intensity of individual region A (G-patch 6′), B (G-patch 6) or C (G-patch 5) and pixel intensity of negative control (ctrl) of the same regions. The automatic background subtraction was performed by Rolling Ball method in 1D gel analysis using ImageQuant TL 8.1. (**C**) Quantification of all regions (A, B and C) together at different concentrations (see Supplementary Fig. S2 for gels) normalized against the control.The values represent a fold change calculated as ratio between pixel intensity of all regions (A, B, C) at different concentrations of compounds and pixel intensity of negative control (ctrl) of all regions (A, B, C). The same background subtraction method was used as in (**B**). n.a.—not analysed, PDS (pyridostatin)—positive control.

The majority of the active candidates selected from the previous experiments (Fig. 5A, the entire gels for all tested compounds can be found in Supplementary Fig. S2) mostly stabilize in the region corresponding to the G-patch 6. Compounds **3**, **6** and **15** show strong stop bands also in G-patch 6′. Some level of stabilization was observed for G-patch 5 as well, especially after incubation with **3** and **6** and partially also with **15** (Fig. 5B, quantification of G-patch regions 5, 6 and 6′). Interestingly, the tested helquats exhibit a different pattern, indicating the involvement of different bases/G-tracts in the stabilized G-quadruplexes.

To evaluate the potency of G-quadruplex-stabilizing compounds, we performed this assay at different compound concentrations (5, 2.5 and 1 μM; Supplementary Fig. S2). This experiment revealed the concentration dependency of the stabilization effect of helquats. The strongest pausing of *Taq* polymerase was observed for **3**, **6**, **15** and partially **12**, which exhibit their stabilization effect even at 1 μM concentration (Fig. 5C).

### Helquats bind specifically to G-quadruplexes, not only to G-rich regions

**Figure 6:**
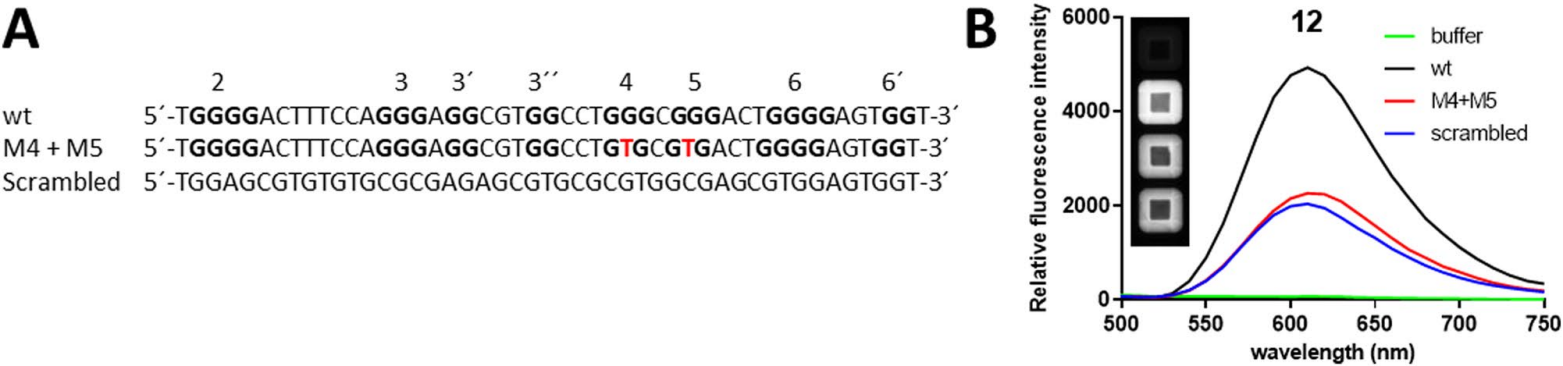
Light-up experiment. (**A**) Sequences of oligonucleotides used in the light-up experiment with the G bases involved in the formation of G-quadruplexes in bold and the G-tracts numbered. The abbreviation ‘wt’ indicates the LTR oligonucleotide with a wild-type sequence capable of folding into G-quadruplex structures. ‘M4 + M5’ indicates the LTR oligonucleotide with two point mutations (highlighted in red) that is unable to form G-quadruplexes. ‘Scrambled’ is the labelling of the LTR oligonucleotide with the identical base composition as the wild-type oligonucleotide, but with a scrambled sequence. (**B**) Emission spectra of **12** after incubation with different oligonucleotides or the reaction buffer alone after excitation at 280 nm. The inset represents a fluorescent image of the wells with compound **12** and a given oligonucleotide after UV-light excitation taken with the Bio-Rad ChemiDoc imaging system.

There are many small molecular probes for specific G4 visualization in cells^49^. Helquats have been shown to have certain optical properties that make them attractive for use as target-specific fluorescence light-up probes for the recognition of targets such as dsDNA or AT-rich DNA sequences^45^. Therefore, we speculated that the most potent helquats presented in our study might show fluorescence light-up in the presence of G-quadruplexes. To distinguish specifically the potential light-up triggered by helquat binding to G-quadruplexes in the HIV-1 LTR, we employed two control oligonucleotides, both unable to form G-quadruplexes (Fig. 6A): an LTR oligonucleotide sequence with two point mutations precluding G4 formation and an LTR oligonucleotide with a scrambled sequence (both adopted from^41^).

Among the compounds tested, only **12** exhibits strong and specific fluorescence after incubation with a quadruplex-forming oligonucleotide (Fig. 6B). The intensity of fluorescence measured after the incubation with both oligonucleotides incompetent of G-quadruplex formation was almost identical, with the maximum intensity 2.5 times lower than that of the G-quadruplex-forming oligonucleotide (wt). This indicates that **12** does not bind non-specifically to G-rich regions but requires and selectively recognizes G-quadruplex formation. To examine the specificity of ligand **12**, we have repeated the light-up experiment with single-stranded G4 LTR in parallel with double-stranded G4 LTR and additional G-quadruplexes (c-myc, c-kit1 and h-telo). We have observed that ligand **12** exhibit strong fluorescence after incubation with a quadruplex forming oligonucleotide ss G4 LTR and only very weak fluorescence after incubation with ds G4 LTR (Supplementary Fig. S3A). The incubation of ligand **12** with other G-quadruplexes showed strong fluorescence in the case of c-myc and c-kit1 oligonucleotides and low fluorescence in the case of h-telo oligonucleotide (Supplement Fig. S3A). This suggests that ligand **12** is not specific to HIV LTR quadruplexes but can bind also to other G-quadruplexes. To further evaluate the specificity of ligand **12**, we performed the light-up titration experiment incubating compound **12** with serial dilutions (1:1) of oligonucleotides (ss G4 LTR wild type, G4 c-myc and c-kit1) spanning final concentrations 2 to 0.002 μM (Supplement Fig. S3B). Here we observed that at oligonucleotides concentration of 0.5 μM and below, compound **12** exhibits stronger fluorescence after incubation with ss G4 LTR than after incubation with c-myc and c-kit1. This can indicate a slight preference of ligand **12** for ss G4 LTR than for c-myc at lower oligonucleotide concentrations.

### Different classes of compounds have distinct modes of binding to G-quadruplexes

**Figure 7.**
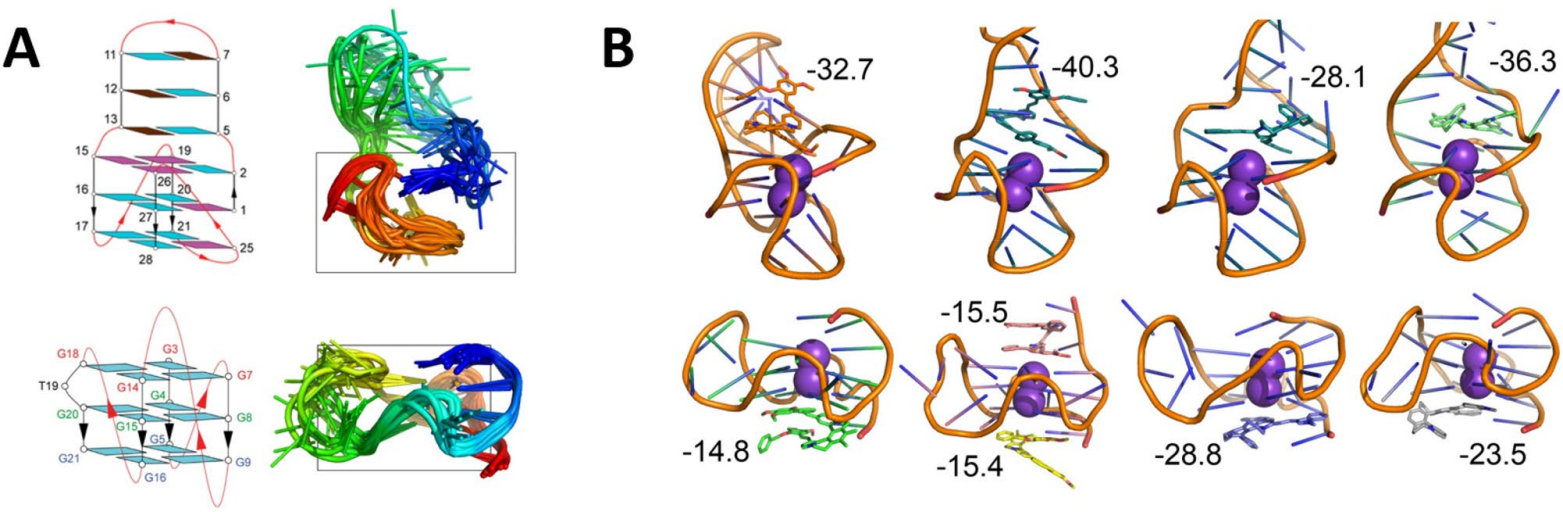
(**A**) Structures of HIV-1 LTR-III (top; PDB: 6H1K)^38^ and LTR-IV (bottom; PDB: 2N4Y)^50^ G4s as determined by NMR. The structures are coloured in a rainbow pattern from the 5′ terminus (blue) to the 3′ terminus (red). (**B**) Computationally predicted most stable poses of **6** and **12** (in sticks) in LTR-III (top row) and LTR-IV (bottom row). G4s are shown as a cartoon, two K+ ions as magenta spheres. The compounds/enantiomers are arranged from left to right: **6/**P, **6**/M, **12**/P, **12**/M. MM-GBSA binding free energies are shown in kcal/mol. For the enantiomer **6**/M, binding to LTR-IV, the iso-energetic poses are shown to be stacked on the top and bottom of the G-quadruplex.

It has already been shown that the promoter region of HIV-1 can form alternative-configuration G4s with different G-patches involved or even induced by G4-binding ligands^11^. The structures of two predominant HIV-1 LTR G4s (LTR-III, comprising G-patches 3–6, and LTR-IV, comprising G-patches 4–6′) have been characterized earlier by NMR spectroscopy^38,50^ and are shown in Fig. 7A. Both G4s are mutually exclusive and show highly distinctive folding and features. Based on the structure of the helquat ‘core’, all the tested compounds can be categorized into two groups: HQ[6,6] dyes (**3**, **6** and **15**) and HQ[7,7] dyes (**12** and **14**). In accordance with previously described results, we selected one representative of each group, namely **6** and **12** (because they are not cytotoxic and also have a different binding patterns to HIV-1 LTR G-patches), and used computational methods to provide a rationale for their interaction with LTR-III and LTR-IV, respectively.

Both enantiomers (M- and P-) of **6** and **12** have been docked into ten conformations of the available NMR structures of HIV-1 LTR-III and LTR-IV^38,50^. For the assessment of their stability, we ran 1 µs molecular dynamics (MD) in explicit solvent. Most of the poses of **6** and **12** docked to LTR-III and LTR-IV were not stable, unbound and rebound on either side of the G-quartet (Supplementary Fig. S4), where they stayed for hundreds of ns. Those poses that were stable in MD bound at the junction of the G-quartet and the stem-loop of LTR-III and stacked on the bottom of the G-quartet of LTR-IV (Fig. 7B). These calculations indicate that the structures and the flexibilities of the individual G4s strongly affect the preferred binding mode of the helquats.

We have observed larger flexibility of the loops in the case of LTR-III (average RMSD of DNA of 6.8 Å) than in LTR-IV (3.6 Å). This behaviour is inherent in the structures of the LTRs (average RMSDs of 6.3 Å and 3.5 Å, respectively, from unliganded LTR MD). It should be noted that the flexibility is attributed to the loops whereas the G-quadruplexes are stable.

The calculations of interaction energy using MM-GBSA have predicted the order of affinity (from the strongest to the weakest) as LTR-III/6 > LTR-III/12 > LTR-IV/12 > LTR-IV/6. This indicates that: (i) both dyes prefer the LTR-III conformation of G4 over the LTR-IV conformation, (ii) compound **6** binds to LTR-IV much more weakly than compound **12**, and (iii) the affinity differences between the M- and P-stereoisomers are larger for LTR-III (7.6 and 8.2 kcal/mol for **6** and **12**, respectively, in favour of the M isomer) than for LTR-IV (0.6 and 5.3 kcal/mol in favour of 6/M and 12/P, respectively). These calculations indicate that the structure of the helquat core strongly affects the binding mode to the individual G4s.

## Discussion

More than 500 helquat-based compounds were synthesized and subsequently screened for their anti-HIV-1 activity. This activity was a factor for the selection of 19 compounds with EC_50_ ˂ 4 µM, whose mechanism of action was then evaluated. After the exclusion of compounds that targeted virus attachment or entry, the timing of the action was determined for the remaining candidates. It was revealed that in order for some compounds (**3**, **6** and **15**) to be active, they had to be administered within 5 h after infection, which corresponded to the time frame of the initial phase of reverse transcription. This implies that they might act via binding to G4s at the RNA level and interfere with the course of reverse transcription. A similar activity time frame was previously published for the G4 ligand BRACO-19, which inhibited the reverse transcription process at the template level^40^. G4s have multiple roles during HIV-1 reverse transcription. It is known that the HIV-1 genome consists of two single-stranded RNA molecules and that both RNA strands are required for the reverse transcription to be successfully completed. HIV-1 RNA dimerises directly without the need for a protein co-factor, and it has been described that this interaction is driven by the formation of an interstrand G4^51^. Furthermore, it is known that the G4 regions of the HIV-1 RNA genome form bi-molecular G4 structures corresponding to HIV-1 recombination hotspots^36^.

Other tested compounds (**12** and **14**) could be administered later (up to 8 h post-infection) and still retain some level of antiviral activity, which provides a sufficient time frame for the completion of reverse transcription. Consequently, they might express their activity also at the DNA level, either before or after integration, but this does not exclude the possibility for them to act at the RNA level as well. It should be noted that the reference compounds in the TOA assay are added at 100-fold their EC_50_ values, but our compounds did not exhibit sufficient selectivity (with the selectivity index ranging between 11.7 and 29.9). Therefore, it is remarkable that we observed such a clear effect even at concentrations only five times greater than their EC_50_ values. Based on the antiviral activity of the G4-binding ligands and its timing, it can be assumed that during viral infection, the G4 structure is formed both in the RNA genome and in the promoter region of the HIV-1 provirus and that it, presumably, has important biological roles in both environments.

The G-cluster in the HIV-1 promotor has previously been shown to be a fine modulator of HIV-1 transcription. Several studies have shown that LTR G4s act as repressor elements of viral transcription, the effect being augmented by G4 ligands^36,40,41^. G-patches 3″–6′ are conserved across HIV-1 sequences, with slight differences in the number of G residues (two to four), in length of linkers (two to five nucleotides) and in their base composition. Interestingly, the common feature shared by all of these sequences is their ability to form stable G4 structures^37^. This observation further supports the assumption that the G4 structure is an important element of the HIV-1 promoter region that is maintained by a strong evolutionary pressure^39^.

It has already been shown that the promoter region of HIV-1 can form alternative G4s with different G-patches involved and that additional conformations of G4 can even be induced by G4-binding ligands^11^ or proteins^52^. The structures of two predominant HIV-1 LTR G4s (LTR-III, comprising G-patches 3–6, and LTR-IV, consisting of G-patches 4–6′) were characterized by NMR spectroscopy^38,50^. The two G4s are mutually exclusive and show very distinctive folding (Fig. 7). Furthermore, their effect on HIV-1 transcription is quite the opposite: The stabilization of LTR-III inhibits viral transcription and thus might promote latency^11^ whereas the formation of LTR-IV increases transcriptional activity and might result in provirus reactivation^50^. These observations have led to the assumption that the balance of LTR-III and LTR-IV may act as a fine regulator of HIV-1 promoter activity.

Indeed, we have observed different stop patterns after the treatment of the LTR template with the compounds studied. All compounds belonging to the HQ[6,6] group of dyes (**3**, **6** and **15**) have shown a similar pattern with strong stop bands at G-patches 6 and, 6′ and weaker *Taq* polymerase pausing also before G-patch 5. By contrast, the representatives of HQ[7,7] group of dyes (**12** and **14**) have forced stop bands only in G-patch 6.

We tried to link these results to LTR structures, dynamics and compound binding. Using molecular dynamics, we confirmed the structural stability of both LTR-III and LTR-IV G4 and the greater flexibility of the loops of the former. We obtained stable binding modes of two selected representatives (**6** from the HQ[6,6] group of dyes and **12** from the HQ[7,7] group of dyes) in both stereoisomeric forms. They were characterized by stacking on one side of the G4s coupled with interactions with the loops. These are typical modes of binding to G4s and have been observed for many ligands by X-ray and NMR methods^53^. The predicted affinities, such as binding free energies, were calculated by the MM-GBSA method as the sum of interaction and deformation free energies (see the “Materials and methods”). These numbers should be interpreted with great caution due to inaccuracies of nucleic-acid and ligand force fields, incomplete sampling and approximations in MD (e.g. the lack of polarization) as well as MM-GBSA (e.g. implicit-solvent) models. Therefore, not even the related, more advanced MM-PBSA method can quantitatively describe G4 conformations from 2.5-µs-long MD simulations^54^. Moreover, entropy effects beyond the scope of the current study, such as the effect of two isoenergetic binding sites on G4 or M/P stereoisomer interconversion in solution, can further contribute to the binding of **6**. These challenges in G4-specific ligand design warrant a multidisciplinary approach involving biology, chemistry and computations.

The regulatory mechanisms of G4s involve not only steric hindrance to transcription and/or translation, but also the binding of the protein factors that modulate G4 conformation and serve as a scaffold for the recruitment of additional protein regulators. Cellular proteins regulate viral latency and effective transcription by inducing or unfolding HIV-1 LTR G4s^41,52,55^. The G4-forming sequence in HIV-1 LTR overlaps with three Sp1 as well as two NFκB binding sites and is crucial for transcription initiation^11^. Sp1 has been shown to bind not only the putative 5′-GGGCGG-3′ sequence in double-stranded DNA, but also the G4 secondary structure^56^, the latter of which it even binds to with a stronger affinity^57^. Sp1 has an ambiguous role in the HIV-1 life cycle: It is a strong activator of provirus expression, but it is also associated with maintaining virus latency^58^. The intriguing fact that HIV-1 LTR possesses two modes of Sp1 binding (G4 structure and consensus sequence) opens new questions about G4 folding and subsequently the role of Sp1 in regulating provirus transcription and HIV-1 latency. Such diversity indicates that HIV-1 uses the G4-forming ability of its promoter region as a complex switching mechanism involving subtle Sp1 interaction differences to fine-tune the transcriptional output. This carefully balanced checkpoint of the HIV-1 life cycle can be exploited as a new target in antiretroviral therapy. In this study, we present a group of chemically similar compounds where subtle modifications of the helquat core lead to a different mode of binding to the G4 region of the HIV-1 promoter. Having strong positive charge these compounds represent poor candidates for antiviral agents at this stage, but they might still serve as lead compounds for further rationale-based drug design. Our experiments show that the presented helquat dyes selectively bind G4s but not only to HIV-1 LTR G4s but can bind also to other G4s e.g. c-myc (as documented by the light-up experiment with **12**). There is a great structural variability of G quadruplexes, differing in the composition and length of the loops and stems^59,60^. It is therefore difficult, if not impossible, to find structural reasons why any ligand, such as helquat would not bind to other types of G quadruplexes. On the other hand, absolute specificity to HIV-1 G4 to achieve antiviral activity might not be necessary^19^. Each infected cells produces many viral genomes, for example the estimated burst size (number of virions released from infected cell) for HIV is between 1000 and 3000 virions per infected cells. Thus we can assume that HIV G4s exceeds the host cells G4s.

Finally, these helquat dyes are also able to distinguish even the distinct G4 conformations (as documented by the different affinities of **6**, and to a lesser extent even of **12**, to LTR-III and LTR-IV quadruplexes). Such diversity could be exploited to design specific ligands able to bind selectively different G4s formed in the HIV-1 LTR and thus specifically regulate the HIV-1 life cycle. Furthermore, these compounds might be a useful tool for determining how and when G4s regulate HIV-1 transcription, which would enhance our understanding of HIV-1 latency and reactivation.

## Materials and methods

### Oligonucleotides, drugs, plasmids and cells

All oligonucleotides were purchased from Sigma-Aldrich (Supplementary Table S1). The following reagents were obtained through the NIH AIDS Reagent Program, Division of AIDS, NIAID, NIH: saquinavir, AMD3100 and raltegravir, MT-4 cells (CD4+ T-cell line) from Dr. Douglas Richman; pNL4-3 plasmid from Dr. Malcolm Martin. HEK293T cells (human embryonic kidney cell line transformed with large T antigen) were obtained from the ATCC (Manassas, VA). MT-4 cells were maintained in RPMI1640 medium with l-glutamine supplemented with 10% fetal bovine serum (FBS), 100 U/ml penicillin, 100 µg/ml streptomycin and 10 mM HEPES (all Sigma-Aldrich). HEK293T cells were maintained in Dulbecco’s Modified Eagle’s Medium (DMEM) with l-glutamine, 10% FBS, 100 U/ml penicillin and 100 µg/ml streptomycin (all Sigma-Aldrich). Cells were grown in a humidified incubator maintained at 37 °C with 5% CO_2_. HEK293T cells were transfected with pNL4-3 to produce NL4-3 HIV-1, as described previously^61^.

### Synthesis and characterization of the compounds tested

The methodology of organic synthesis, together with the structures, MS analysis, NMR characterization and evaluation of the anti-HIV-1 activity of the tested compounds, is described in the Supplementary Information.

### Adsorption and replication assays

MT-4 cells were seeded in 80 µl of the Roswell Park Memorial Institute (RPMI) medium in the amount of 2 × 10^5^ cells per well of a 96-well plate, and 10 µl of the virus stock were immediately added with the final multiplicity of infection (MOI) of 0.05. For a replication assay, the infected cells were incubated at 37 °C for 1 h before the addition of compounds being tested (10 µM final concentration) and subsequently cultivated at 37 °C for another 5 days. For an adsorption test, by contrast, the compounds (10 µM final concentration) were added simultaneously with the virus, and after 2 h of incubation, the cells were washed three times with PBS to remove the compounds and unbound virions; after the addition of 100 µl of the RPMI medium, the cells were cultivated at 37 °C for 5 days. Five days post-infection, aliquots of virus-containing cell-free supernatants were harvested and analysed by reverse transcriptase (RT) assay (described in the Supplementary Information). The experiments were performed as biological replicates where the sample size for each condition was n = 3.

### Time-of-addition assay

MT-4 cells were infected with NL4-3 HIV-1 at a MOI of 1. After 1 h, the cells were washed, seeded (100,000 cells/well) and incubated at 37 °C. The tested compounds were added at 2, 3, 5, 8, 12 and 24 h post-infection at 10 μM final concentration, while the reference compounds were added at a concentration corresponding to 100-fold their EC_50_. HIV-1 production was determined 31 h post-infection by measuring RT activity in the supernatant.

### FRET melting assay

The G4 LTR FRET oligonucleotide was diluted to 0.4 μM in LiCaco buffer (10 mM lithium cacodylate at pH 7.2 and 100 mM KCl), heat-denatured at 95 °C for 5 min, and folded into a G4 structure at room temperature for 4 h. After incubation, the LiCaco buffer was added alone or with the compounds being tested (4 μM in LiCaco buffer and 1% DMSO). The final concentrations for compound screening were: 0.2 μM oligonucleotide, 2 μM tested compound, 100 mM KCl and 0.5% DMSO. Fluorescence melting curves were determined using a Realplex^4^ Mastercycler (Eppendorf). After the first equilibration step at 25 °C for 5 min, a stepwise increase of temperature with a resolution of 0.1 °C was performed to reach 95 °C, with FAM emission monitored in each step. T_m_ was either calculated directly by the Realplex^4^ Mastercycler, or raw data were analysed in GraphPad Prism 7, where the asymmetric (five-parameter) model was used to obtain melting temperatures T_m_ (°C) for the further calculation of ΔT_m_. The ΔT_m_ was calculated by subtracting the average T_m_ of PBS (control) from the average T_m_ for each compound. Three independent experiments were performed.

### *Taq* polymerase stop assay

A *Taq* polymerase stop assay was carried out as described in a previous study^11^. Briefly, the primer (G4 LTR primer, Supplementary Table S1) was 5′-end labelled with [γ-^32^P]ATP using T4 polynucleotide kinase at 37 °C for 30 min. The T4 polynucleotide kinase was then inactivated by incubation at 65 °C for 5 min. The radioactively labelled primer was column-purified and stored at − 20 °C for further use. The labelled primer (1.7 pmol) was annealed to the template (2.1 pmol) in the LiCaco buffer and extended with the Go *Taq* DNA polymerase (2U/reaction, Promega) at 45 °C for 45 min. Samples were incubated with G-quadruplex-ligands at 45 °C for 45 min before DNA polymerase addition. The reactions were stopped by a denaturing solution (80% formamide, 10% glycerol, 0.025% xylen cyanol, 0.025% bromphenol blue and 10 mM EDTA pH 8); extension products were separated on 15% denaturing gel and visualized by phosphorimaging. The pixel intensity of bands was calculated using 1D gel analysis in ImageQuant TL software version 8.1 (GE HealthCare) after automatic background subtraction by Rolling Ball method.

### Light-up experiments

The selected compounds were incubated in the presence of the G4 LTR wild-type (wt) oligonucleotide, the G4 LTR M4 + M5 oligonucleotide containing mutations that disabled G-quadruplex formation (described previously^41^), and the G4 LTR scrambled-sequence oligonucleotide, or the buffer-only control. The compounds were tested at a compound:oligonucleotide ratio of 2:1 (4 μM compounds and 2 μM oligonucleotide) in PBS in a 384-well plate. Emission spectra were measured after excitation at 280 nm in a Tecan Infinite M1000 plate reader. The fluorescent image of the plate was taken with the Bio-Rad ChemiDoc imaging system after UV-light excitation. The Light-up titration experiment was performed similarly as described above with the exception that 4 μM compound 12 was incubated with serial dilutions (1:1) of oligonucleotides (ss G4 LTR wild type, G4 c-myc and G4 c-kit1) spanning final concentrations 2 to 0.002 μM. The excitation at 280 nm and emission at 600 nm were used to monitor the Light-up.

### Computational modelling

Both P- and M-enantiomers of compounds 6 and 12 were docked to all 10 + 10 conformations of the available NMR structures of G4s (LTR-III: 6H1K, LTR-IV: 2N4Y) using Glide SP^62^. Based on visual inspection and the docking score, 49 of the resulting complexes were selected for further evaluation. To assess the stability of the binding modes, 1 µs molecular dynamics (MD) simulations were run in an explicit solvent for the complexes under study, free ligands and G4s. The ligands were parametrized by the antechamber module of AMBER (ambertools20, forcefield: GAFF2 [Amber 2020, University of California, San Francisco] with RESP charges (HF/6-31G*) calculated using the Gaussian 16 package (Gaussian 16, revision A.03, Gaussian, Inc., Wallingford CT, 2016). The systems were prepared in Leap (DNA forcefield: OL15, water model: TIP3P), neutralized with Na^+^, and the NaCl concentration was adjusted to 0.15 M. Hydrogen mass repartitioning was applied to make it possible to use a time step of 4 fs. The systems were simulated using the GPU-accelerated pmemd code in AMBER20 in the following steps: 1000 cycles of minimization, 50 ps of heating to 300 K with the solute restrained (force constant: 2.0 kcal/mol/Å^2^), 200 ps of NpT equilibration with the solute restrained (force constant: 2.0 kcal/mol/Å^2^), 500 ps of NpT equilibration with the solute restrained (force constant: 0.1 kcal/mol/Å^2^) and 1 µs of production without restraints, using a time step of 4 fs. The stability of the 500 frames obtained was analysed using pytraj, and the stable poses were subjected to binding free energy calculations using the MM-GBSA approach^63^ by MMPBSA.py in ambertools20 with default parameters except for ionic strength, which was set to 0.1 M (istrng = 0.1). The individual terms were calculated according to Eqs. (1–4).

1 $${\text{binding energy }} = {\text{ E}}\left( {{\text{complex}}} \right) - {\text{E}}_{{{\text{min}}}} \left( {{\text{DNA}},{\text{ free}}} \right) \, - {\text{ E}}_{{{\text{min}}}} \left( {{\text{ligand}},{\text{ free}}} \right)$$

2 $${\text{interaction energy }} = {\text{ E}}\left( {{\text{complex}}} \right) \, - {\text{ E}}\left( {{\text{DNA}},{\text{ cplx}}} \right) \, - {\text{ E}}\left( {{\text{ligand}},{\text{ cplx}}} \right)$$

3 $${\text{deformation energy }}\left( {{\text{DNA}}} \right) = {\text{ E}}\left( {{\text{DNA}},{\text{cplx}}} \right) \, - {\text{ E}}_{{{\text{min}}}} \left( {{\text{DNA}},{\text{ free}}} \right)$$

4 $${\text{deformation energy }}\left( {{\text{ligand}}} \right) \, = {\text{ E}}\left( {{\text{ligand}},{\text{cplx}}} \right) \, - {\text{ E}}_{{{\text{min}}}} \left( {{\text{ligand}},{\text{ free}}} \right)$$

## Supplementary Information

Supplementary Information.

## Data Availability

All data generated and analysed during this study are included in this published article (and its Supplementary Information files).
