# Supplementary material for: Helquat dyes targeting G-quadruplexes as a new class of anti-HIV-1 inhibitors
Source: Sci Rep. 2023 Apr 13;13:6096. doi: 10.1038/s41598-023-33263-3 (PMC10102027; doi:10.1038/s41598-023-33263-3)
Supplement: Supplementary file 1 — Supplementary Information. [file 41598_2023_33263_MOESM1_ESM.pdf]

SUPPLEMENTARY DATA

Supplementary Table 1 – The oligonucleotides used in this study.

Purpose	Name	Sequence
FRET	G4 LTR FRET	FAM-5'-TGGGGACTTTCCAGGGAGGCGTGG CCTGGGCGGGACTGGGGAGTGGT-3'-TAMRA
DNA stop	G4 LTR Stop	5'-TTTTTGGGGACTTTCCAGGGAGGCGTGGCCTGGGCGGG ACTGGGGAGTGGTTTTTCTGCATATAAGCAGCTG CTTTTTGCC-3'
	G4 LTR Stop primer	5'-GGCAAAAAGCAGCTGCTTATATGCAG-3'
light up	ss G4 LTR wt	5'-TGGGGACTTTCCAGGGAGGCGTGGCCTGGGCGGGACTGGGGAGTGGT-3'
	scrambled	5'-TGGAGCGTGTGTGCGCGAGAGCGTGCGCGTGGCGAGCGTGGAGTGGT-3'
	M4 + M5	5'-TGGGGACTTTCCAGGGAGGC GTGGCCTGTGCGTGACTGGGGAGTGGT-3'
	ds G4 LTR wt	5'-TGGGGACTTTCCAGGGAGGCGTGGCCTGGGCGGGACTGGGGAGTGGT-3'<br>5'-ACCACTCCCCAGTCCCGCCCAGGCCACGCCTCCCTGGAAAGTCCCCA-3'
	c-myc (Pu27)	5'-TGGGGAGGGTGGGGAGGGTGGGGAAGG-3'
	c-kit1	5'-GGGAGGGCGCTGGGAGGAGGG-3'
	h-telo	5'-GGGTTAGGGTTAGGGTTAGGG-3'

SUPPLEMENTARY METHODS

XTT screening. MT-4 cells were seeded in 80 µl of the RPMI without Phenol Red in the amount of $3x10^4$ cells per well of a 96-well plate. 10 µl of tested compounds (500 µM at 5% DMSO) or controls (5% DMSO at PBS, AZT 1mM, SQV 100 µM) were added to the cells; after one-hour incubation at 37 °C, HIV-1 (NL4-3 strain) was added with MOI = 0.05 and the infected cells were cultivated for five days. The viability of the cells was measured by the XTT cell viability and proliferation assay. Briefly, 50 µl of the XTT solution with the addition of PMS (both VWR) was added to the cultivation media and incubated in a humidified incubator maintained at 37 °C with 5% CO_2 for four hours, after which absorbance at 560 nm was measured on a VICTOR plate reader (PerkinElmer).

The cytotoxicity of the tested compounds was evaluated separately without virus addition and analysed by the same assay.

For compounds selected as hits in the initial screening, the effective concentration that inhibits 50% of the viral activity (EC_{50}) and the cytotoxic concentration that reduces the MT-4 cell viability by 50% (CC_{50}) were determined. Viral activity was evaluated after five days as an HIV-1-induced cytopathic effect quantified by the XTT assay. Selected hits were tested at two-fold serial dilutions from 50 µM concentration, and the drug concentrations required to reduce the viral cytopathic effect by 50% (EC_{50}) as well as the drug concentrations that reduced MT-4 cell viability by 50% (CC_{50}) were calculated by nonlinear regression using GraphPad Prism v.8.0.0 (GraphPad Software).

RT assay. The reverse transcriptase (RT) activity in a cell-free supernatant was assessed by an α-^{32}P RT assay. Briefly, the RT activity in the culture medium, which corresponded to the amount of HIV-1, was used to produce a DNA copy of an RNA template by extending a complementary oligo dT DNA primer with radiolabelled nucleotides. A reaction mixture containing 50 mM Tris-HCl pH 7.8, 75 mM KCl, 2 mM DTT, 5 mM $MgCl_2$ and 0.5% NP-40 (all Sigma-Aldrich), 5 µg per ml of poly (rA)-poly (dT) (The Midlan Certified Reagent Company) with 1 µl 10-mCi/ml [α-^{32}P]dTTP (American Radiolabeled Chemicals) per ml, and the tested sample was incubated at 37°C for two hours, after which 10 µl were spotted on DEAE blotting paper (PerkinElmer) and air-dried. After five washes with SSC buffer (0.15 M NaCl, 0.015 M sodium citrate) and three additional washes with 85% ethanol, the blotting paper was exposed to Phosphoscreen overnight, which was scanned on a Typhoon 9410 Phosphorimager instrument (GE Healthcare). The intensity of the dots was analysed by the ImageQuant software (GE Healthcare).

SUPPLEMENTARY FIGURES

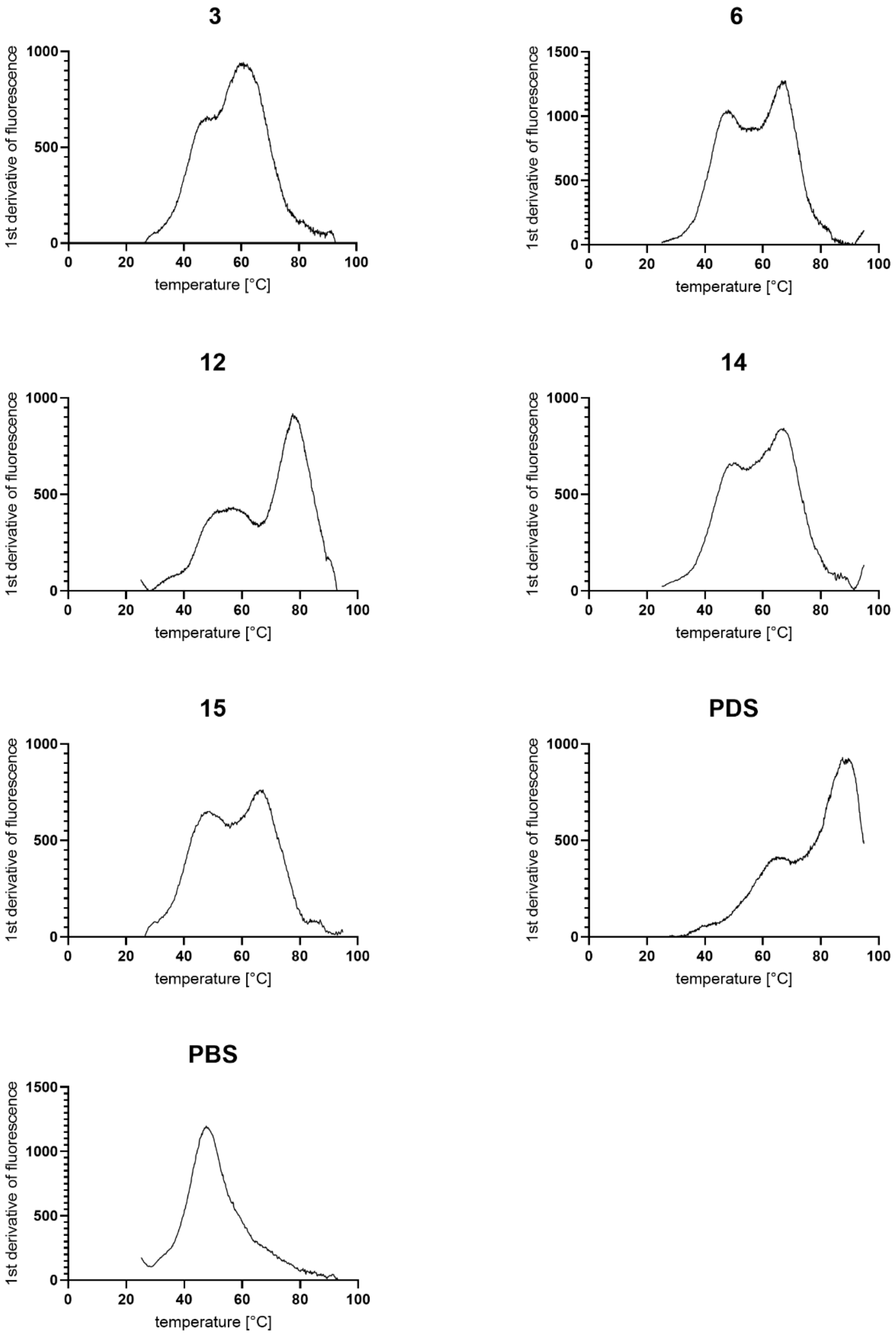

Supplementary Figure S1. Representative FRET melting curves for compounds reported in the table 1.

A

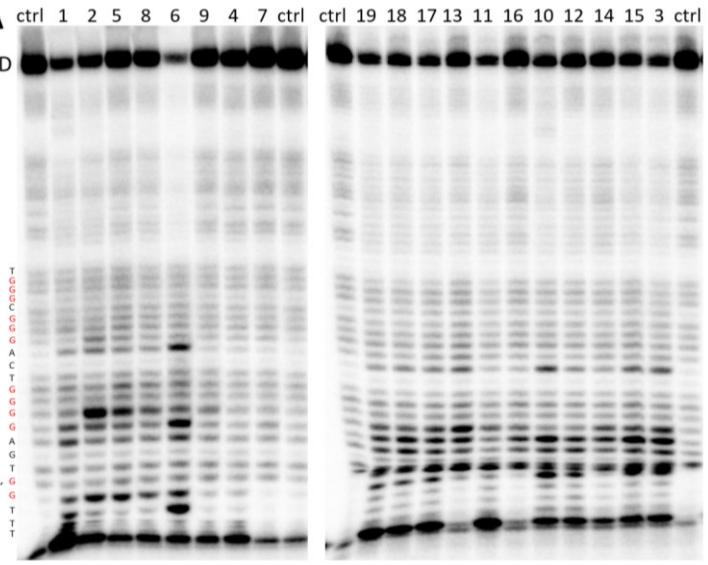

B

	D	C	B	A
ctrl	18928035	2749029	4289700	2069533
1	6862456	4225062	8425673	5368738
2	8247352	5606750	11455225	5765155
5	15608278	5248048	10277443	5521941
8	12826794	4260635	8246324	4508157
6	4462646	6011127	11702152	9114581
9	16130817	3295804	6363665	2961838
4	15516102	2881991	4476400	2678852
7	22016570	2939745	4717447	2013196
ctrl	21936296	2631897	3867370	1739809
ctrl	18069417	2392837	3178729	1839184
19	6009731	3284815	6585634	5129526
18	7293912	3475595	7801825	4840905
17	5879355	3900491	7639103	5795372
13	10414888	4880397	9006611	5030272
11	5311721	2899070	5428375	4768835
16	13776341	3374470	6778513	3180583
10	7779292	4458404	8054971	6472843
12	11435966	3479578	6732664	4865141
14	9907291	3628729	7153935	3617639
15	7484151	4519815	9762307	6260686
3	5935729	4372455	9433310	5902747
ctrl	18313152	2420011	3463566	1982952

C

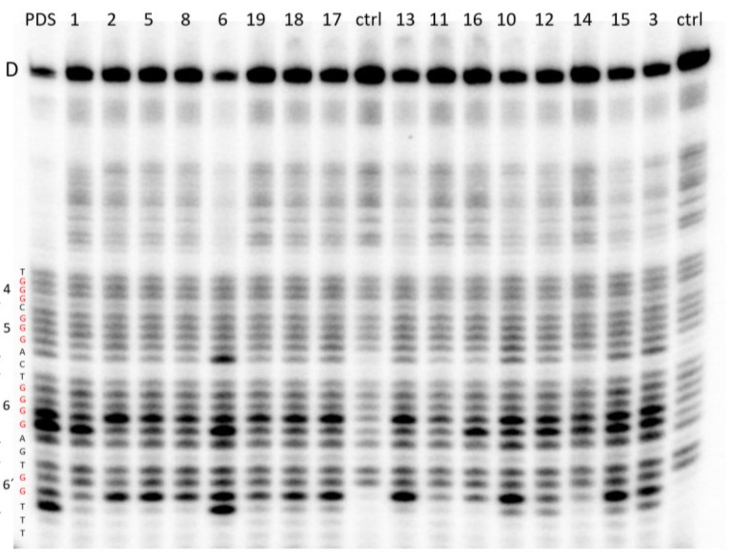

D

	D	C	B	A
PDS	4013259	6791905	17286606	9737861
1	10114788	5823687	13697850	6171996
2	10336782	6802639	12934453	7029007
5	11564459	6205948	12432192	8299423
8	10583889	5975290	11890509	7456786
6	4716714	8494200	16054622	12474560
19	13605045	6098757	11209303	6646798
18	10994508	6466926	12256080	7692739
17	10032551	6898988	12430781	8067530
ctrl	18437287	4268692	6281573	3604764
13	8044681	6628513	12718577	9206810
11	13103955	5373169	9371239	4972857
16	11692718	5918431	11756537	5629060
10	7794279	7838376	14488330	9934385
12	9255650	6411986	13463489	6976648
14	13431713	6458087	12672740	5239863
15	7010537	7483727	16166787	10582066
3	7629524	7190827	15287545	8914848
ctrl	19445475	4278450	6474985	3561534

E

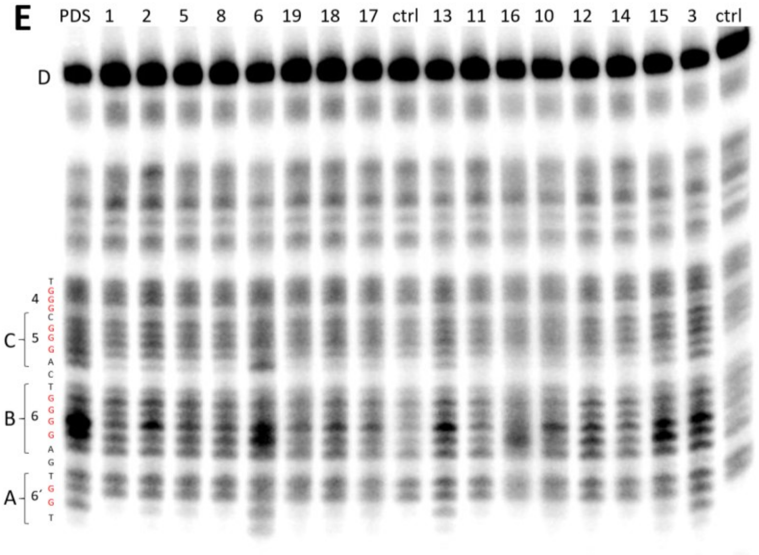

F

	D	C	B	A
PDS	10395976	8793586	15177445	5221091
1	20490856	6013423	9787961	3838013
2	19601695	7150295	10733738	4440611
5	18244992	6264740	10248276	4443768
8	17779838	6011287	9935836	4345995
6	13110472	8238914	13324783	6365788
19	19450010	6048610	9355748	4375928
18	19695161	6517671	10161465	4599369
17	16605486	5812993	8722802	4091224
ctrl	17889109	4696764	6694547	3588289
13	13570174	6421794	10893606	4891467
11	17637124	5154164	7879806	3784814
16	12698644	5483547	10452168	3622831
10	14119239	6174278	10733753	4506942
12	15806283	5749258	10185489	4307860
14	17691866	5228733	8362282	3864685
15	14284253	6658261	12346739	4696888
3	12897569	7484784	12154621	5607465
ctrl	19131543	4703769	6614323	3621019

Supplementary Figure S2. A Taq polymerase stop assay for all the tested compounds at three different concentrations (A – 5 µM, C – 2.5 µM, E – 1 µM) and the respective Image Quant quantification of the selected regions. PDS (pyridostatin) was used as a positive control in the same concentration as the tested compounds, ctrl (buffer only). The brackets labelled with letters A, B and C mark the regions selected for quantification by ImageQuant TL, letter D marks the final product of the reaction, which was also quantified and used for subsequent normalization.

Figure 5 in the main text is derived from part C of this supplementary figure by the excision of selected lanes (lane PDS, ctrl, 3, 6, 12, 14 and 15).

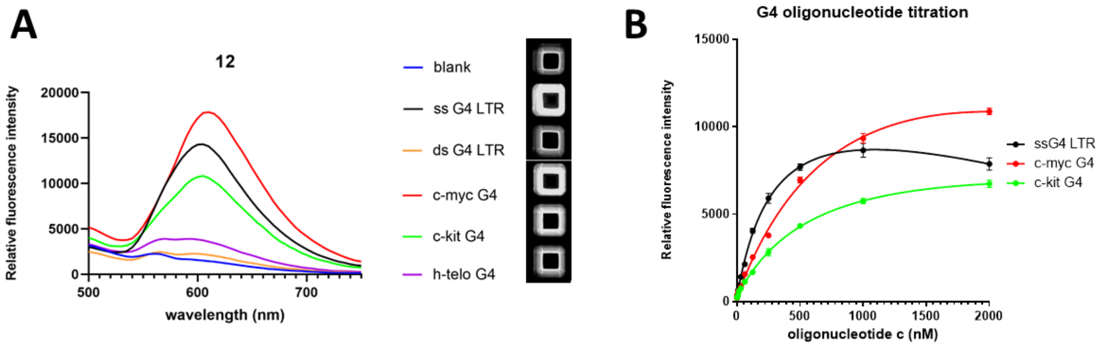

Supplementary Figure S3. Light-up and light-up titration experiments. (A) Emission spectra of compound 12 after incubation with different oligonucleotides or alone after excitation at 280 nm. The inset on the right side represent a fluorescent image of the wells with compound 12 and a given oligonucleotide after UV-light excitation taken with the Bio-Rad ChemiDocTM imaging system. (B) Emission at 600 nm of compound 12 after incubation with serial dilutions of oligonucleotides (ss G4 LTR wild type, G4 c-myc and G4 c-kit1) spanning final concentrations 2 to 0.002 µM after excitation at 280 nm.

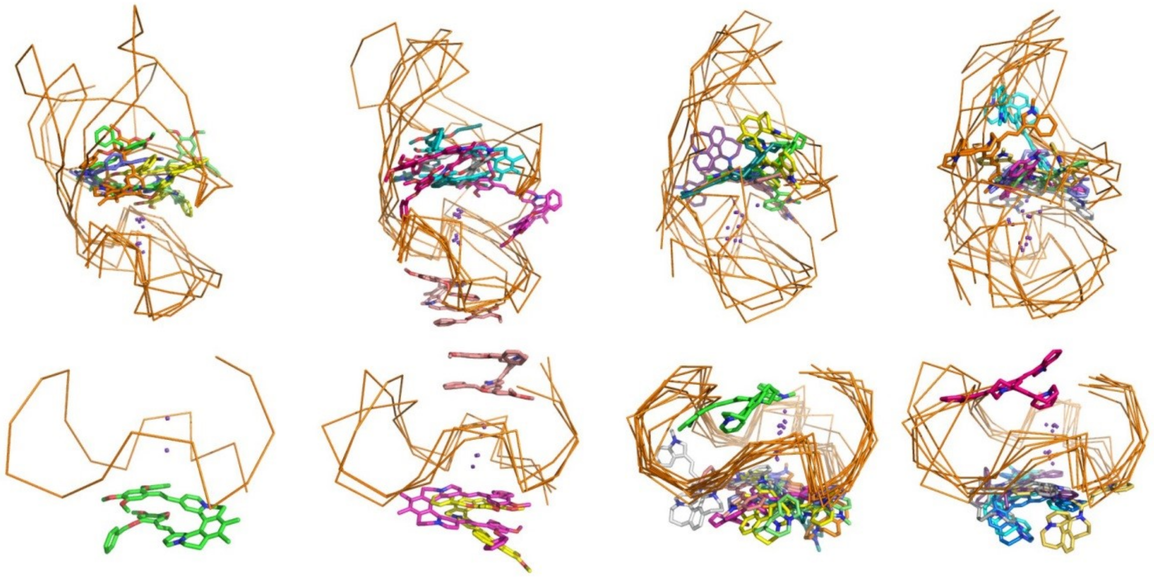

Supplementary Figure S4. The stable poses of **6** (coloured sticks; P-enantiomer – leftmost, M-enantiomer – middle left) and **12** (coloured sticks; P-enantiomer – middle right, M-enantiomer – rightmost) in LTR-III (orange wireframe; top) and LTR-IV (orange wireframe; bottom). Figure rendered with PyMol, ver. 1.7.6.

ORGANIC SYNTHESIS METHODS

The methods of compound synthesis and the MS and NMR spectra documenting the synthesis and purification process are listed in the order in which the compounds are labelled (1 to 19).

1 - PD314

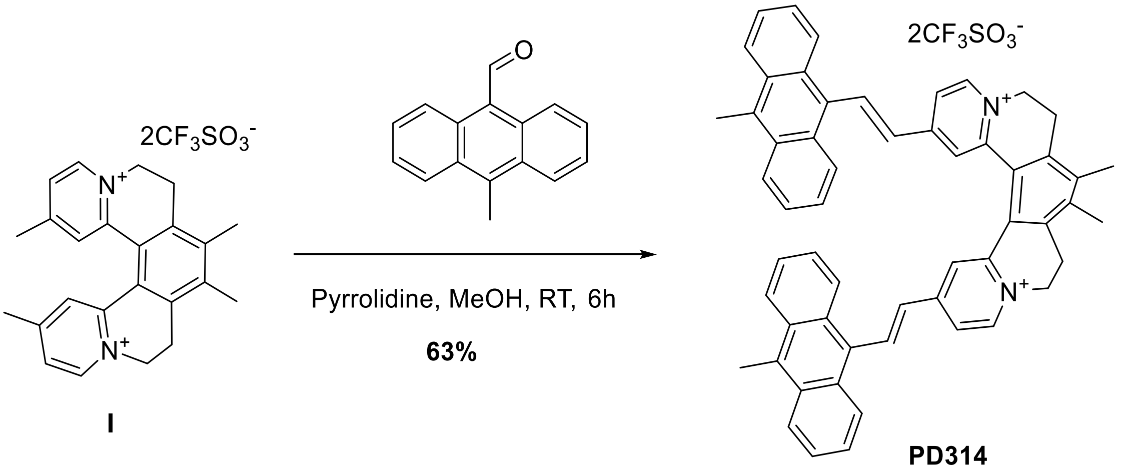

Helquat **I** (30 mg, 0.047 mmol), 10-methylanthracene-9-carbaldehyde (208 mg, 1.4 mmol, 30.0 equiv.), pyrrolidine (0.07 mL, 0.70 mmol, 15.0 equiv.) and MeOH (2.0 mL) were placed in a 10-mL flask and stirred under argon at room temperature for six hours while being protected from ambient light using an aluminium-foil cover. The reaction progress was checked by TLC (mobile-phase Stoddart's magic mixture). The crude product was transferred to 50-mL centrifuge tubes and precipitated from the reaction mixture by the addition of Et_2O (30 mL to each tube). The resulting suspensions were centrifuged and supernatants were removed. Residues were dissolved in a minimum amount of MeOH (1.0 mL), after which Et_2O (20 mL) was added to each tube. Precipitates were centrifuged. This reprecipitation was repeated three more times. Centrifuged solids were collected in a glass vial using Et_2O. The resulting suspension was centrifuged and Et_2O was removed. The solids were dried under vacuum to obtain pure **PD314** as a red solid in 63% yield (31 mg, 0.03 mmol).

^{1}H NMR (401 MHz, acetonitrile-d_3) δ 8.82–8.78 (m, 2H), 8.53 (d, J = 16.5 Hz, 2H), 8.36 (dt, J = 8.9, 1.0 Hz, 4H), 8.24–8.18 (m, 4H), 8.08 (dt, J = 8.8, 1.0 Hz, 4H), 7.49 (ddd, J = 9.0, 6.5, 1.2 Hz, 4H), 7.34 (ddd, J = 8.9, 6.5, 1.2 Hz, 4H), 7.09 (d, J = 16.5 Hz, 2H), 4.95–4.86 (m, 2H), 4.73 (td, J = 14.1, 13.7, 3.8 Hz, 2H), 3.49 (ddd, J = 17.4, 3.9, 1.8 Hz, 2H), 3.19–3.09 (m, 2H), 3.08 (s, 6H), 2.46 (s, 6H).

^{13}C NMR (101 MHz, CD_3CN) δ 152.89, 148.48, 146.27, 141.70, 140.37, 139.74, 134.36, 132.46, 130.69, 130.06, 129.12, 127.47, 127.03, 126.49, 126.47, 126.44, 125.26, 122.91, 54.75, 26.44, 17.08, 14.82.

MS (ESI+) m/z (%): 373 (100%, Z = 2)

HRMS (ESI+) m/z: [(M-2TfO$^-$)$^+$] ($C_{56}H_{46}N_2$, Z = 2) calc.: 373.18250, found: 373.18271.

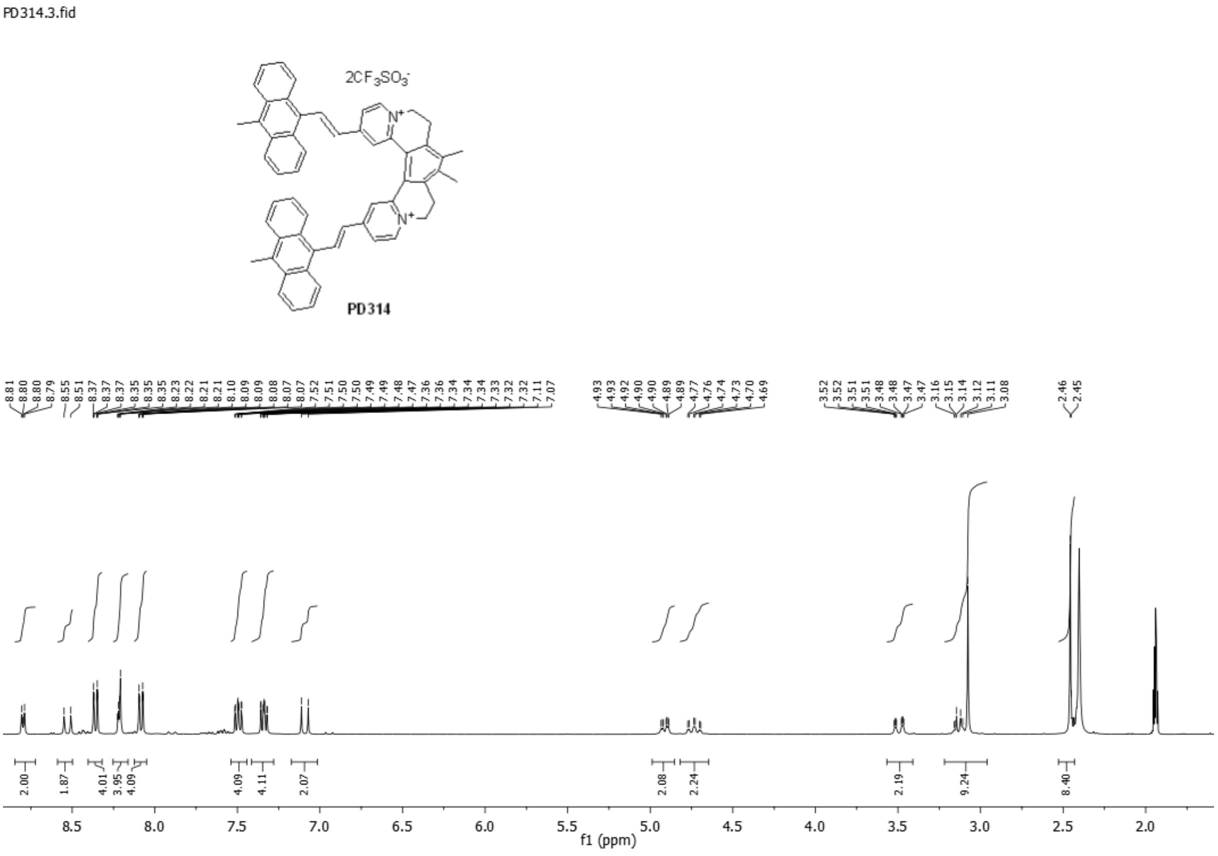
PD314.3.fid
2CF3SO3-
PD314
8.81 8.80 8.80 8.79 8.55 8.51 8.37 8.37 8.37 8.35 8.35 8.35 8.23 8.22 8.21 8.21 8.10 8.09 8.09 8.08 8.07 8.07 7.52 7.51 7.50 7.50 7.49 7.49 7.48 7.47 7.36 7.36 7.34 7.34 7.34 7.33 7.32 7.32 7.11 7.07
4.93 4.93 4.92 4.90 4.90 4.89 4.89 4.77 4.76 4.74 4.73 4.70 4.69
3.52 3.52 3.51 3.48 3.48 3.47 3.16 3.15 3.14 3.12 3.11 3.08
2.46 2.45
2.00 1.87 4.01 3.95 4.09 4.09 4.11 2.07 2.08 2.24 2.19 9.24 8.40
f1 (ppm)

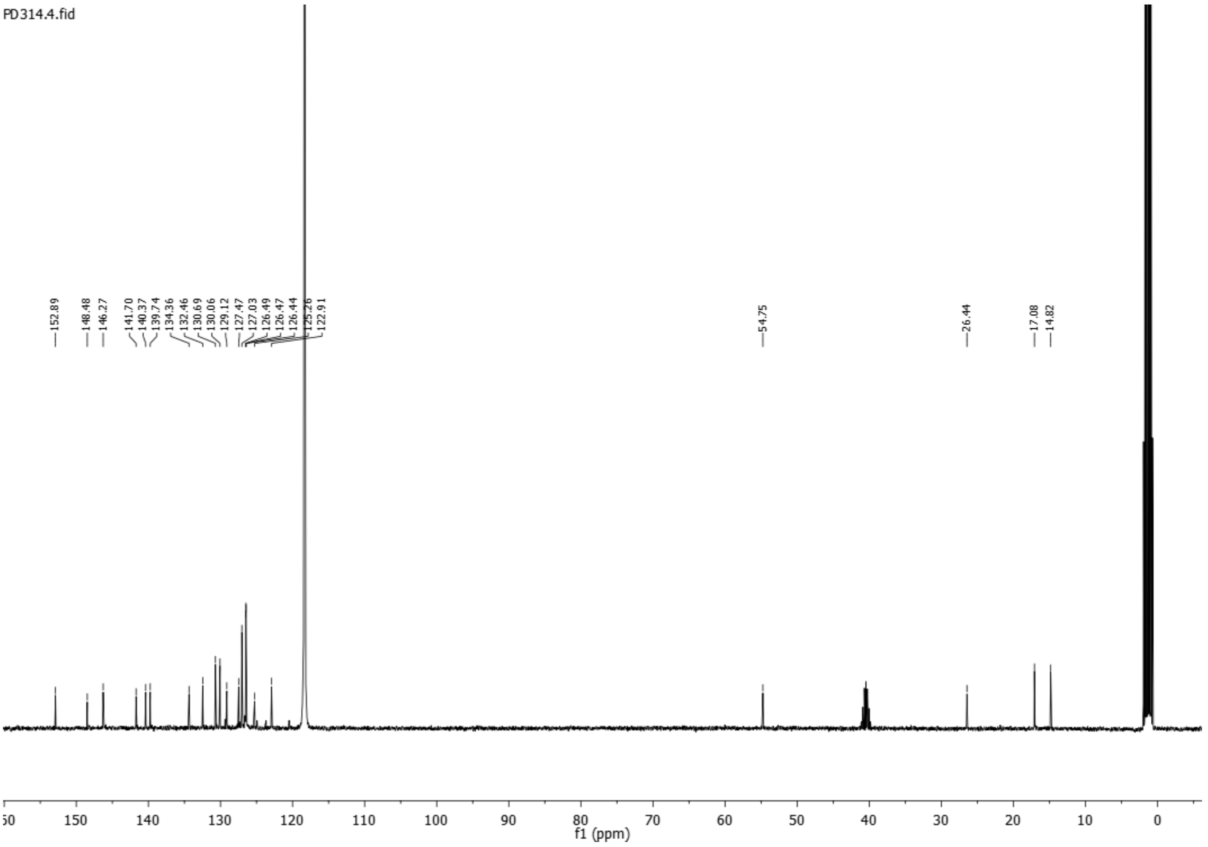
PD314.4.fid
152.89 148.48 146.27 141.70 140.37 139.74 134.36 132.46 130.69 130.06 129.12 127.47 127.03 126.49 126.47 126.44 125.26 122.91
54.75
26.44
17.08 14.82
f1 (ppm)

2 - PR638

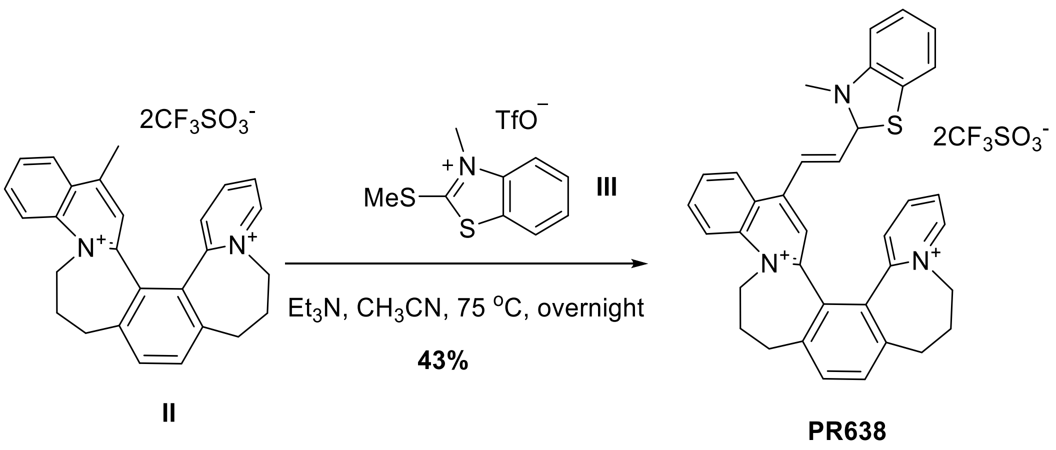

Helquat **II** (30 mg, 0.047 mmol), **III** (62 mg, 0.178 mmol, 8.0 equiv.), Et_3N (0.025 mL, 0.18 mmol) and CH_3CN (1.5 mL) were placed in a 10-mL flask and stirred under argon at 75 °C overnight while being protected from ambient light using an aluminium-foil cover. The reaction progress was checked by TLC (mobile-phase Stoddart's magic mixture). The crude product was transferred to 50-mL centrifuge tubes (three tubes with 0.5 mL of reaction crude each) and precipitated from the reaction mixture by the addition of Et_2O (25 mL to each tube). The resulting suspensions were centrifuged and supernatants were removed. Residues were dissolved in a minimum amount of CH_3CN (0.5 mL added to each tube), after which Et_2O (25 mL) was added to each tube. Precipitates were centrifuged. This reprecipitation was repeated twice. Centrifuged solids were collected in a glass vial using Et_2O. The resulting suspension was centrifuged and Et_2O was removed. The solids were dried under vacuum to obtain pure **PR638** as a violet solid in 43% yield (16 mg, 0.019 mmol).

^{1}H NMR (401 MHz, acetonitrile-d_3) δ 8.99 (dd, *J* = 6.2, 1.5 Hz, 1H), 8.52 (dd, *J* = 8.7, 1.4 Hz, 1H), 8.22–8.13 (m, 2H), 8.01 (ddd, *J* = 8.7, 7.0, 1.4 Hz, 1H), 7.95 (dt, *J* = 8.0, 0.9 Hz, 1H), 7.85 (ddd, *J* = 7.7, 6.1, 1.5 Hz, 1H), 7.77–7.70 (m, 3H), 7.66–7.57 (m, 3H), 7.46 (ddd, *J* = 8.2, 7.1, 1.3 Hz, 1H), 6.71 (s, 1H), 6.45 (s, 1H), 5.06 (ddd, *J* = 23.2, 14.2, 6.0 Hz, 2H), 4.90 (td, *J* = 12.7, 5.2 Hz, 1H), 4.28 (ddd, *J* = 15.3, 13.2, 5.0 Hz, 1H), 3.86 (s, 3H), 3.04 (ddd, *J* = 17.2, 13.4, 5.9 Hz, 2H), 2.84–2.44 (m, 3H), 2.29 (dt, *J* = 13.8, 6.6 Hz, 2H).

^{13}C NMR (101 MHz, CD_3CN) δ 160.63, 154.41, 151.58, 148.57, 146.37, 146.20, 141.58, 140.22, 139.80, 139.33, 134.88, 134.75, 134.28, 133.75, 133.34, 130.51, 129.65, 129.01, 128.15, 126.42, 125.91, 124.83, 124.33, 123.68, 120.53, 118.87, 114.04, 113.82, 89.82, 59.19, 49.23, 34.82, 32.41, 30.32, 30.01.

MS (ESI+) m/z (%): 269 (100%, Z = 2)

HRMS (ESI+) m/z: [$(M-2TfO^-)^+$] ($C_{36}H_{33}N_3S$, Z = 2) calc.: 269.14645, found: 269.14673.

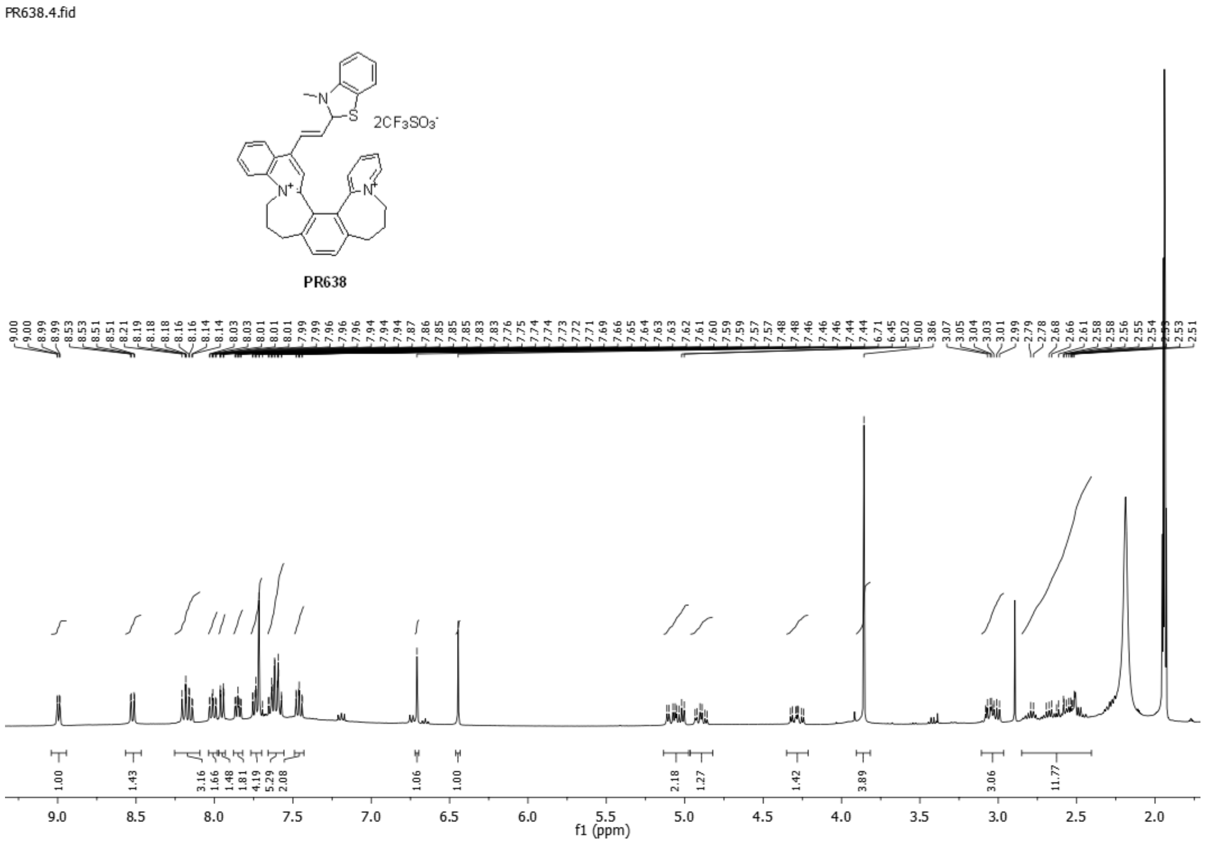
PR638.4.fid
2CF3SO3-
PR638
f1 (ppm)

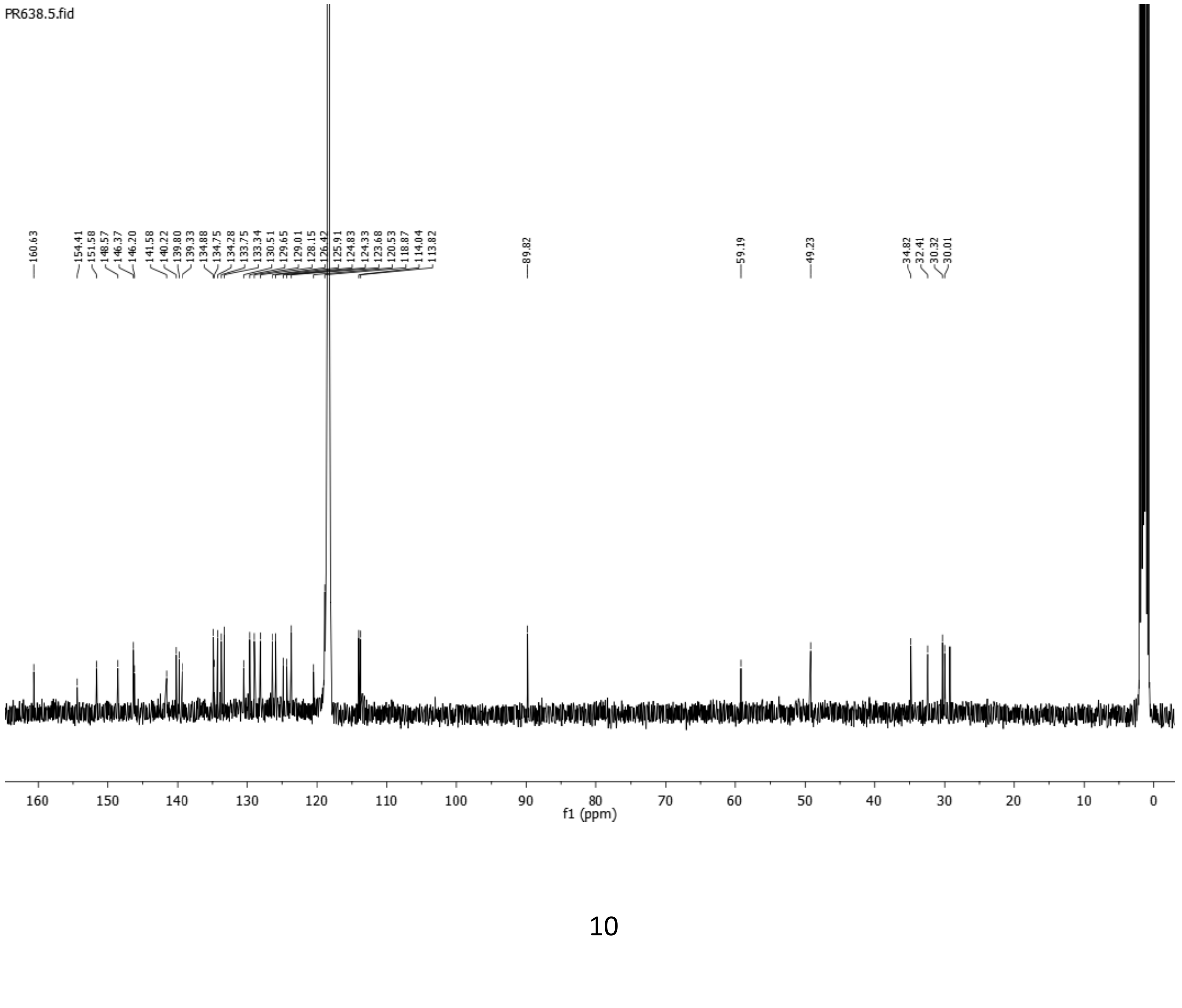
PR638.5.fid
f1 (ppm)

3 - PR673

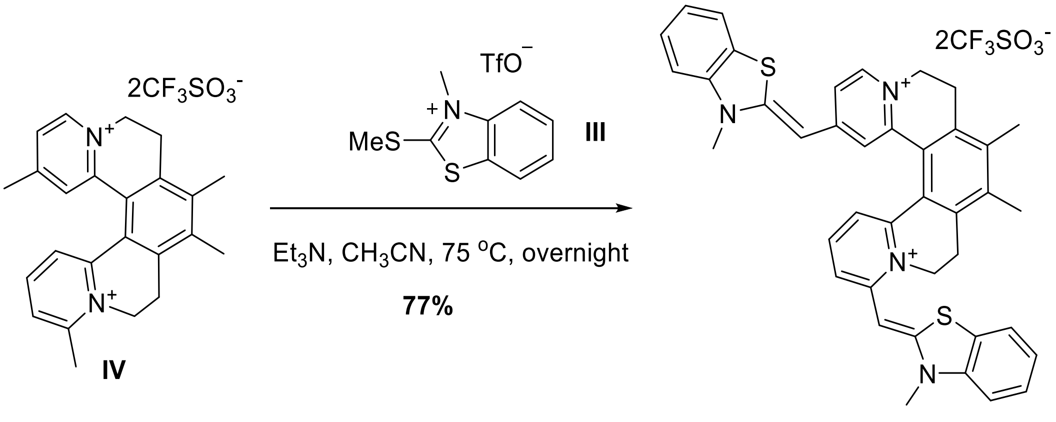

PR673

Helquat **IV** (30 mg, 0.047 mmol), **III** (131 mg, 0.38 mmol, 8.0 equiv.), Et_3N (0.052 mL, 0.038 mmol) and CH_3CN (1.5 mL) were placed in a 10-mL flask and stirred under argon at 75 °C overnight while being protected from ambient light using an aluminium-foil cover. The reaction progress was checked by TLC (mobile-phase Stoddart's magic mixture). The crude product was transferred to 50-mL centrifuge tubes (three tubes with 0.5 mL of reaction crude each) and precipitated from the reaction mixture by the addition of Et_2O (25 mL to each tube). The resulting suspensions were centrifuged and supernatants were removed. Residues were dissolved in a minimum amount of CH_3CN (0.5 mL added to each tube), after which Et_2O (25 mL) was added to each tube. Precipitates were centrifuged. This reprecipitation was repeated twice. Centrifuged solids were collected in a glass vial using Et_2O. The resulting suspension was centrifuged and Et_2O was removed. The solids were dried under vacuum to obtain pure **PR673** as a light-red solid in 77% yield (34 mg, 0.036 mmol).

^{1}H NMR (401 MHz, acetonitrile-*d*$_3$) δ 8.04 (d, *J* = 7.0 Hz, 1H), 7.77 (dd, *J* = 8.7, 1.2 Hz, 1H), 7.67–7.50 (m, 3H), 7.49–7.44 (m, 2H), 7.25 (dtd, *J* = 12.9, 7.5, 1.2 Hz, 4H), 7.20–7.14 (m, 1H), 6.97 (d, *J* = 2.3 Hz, 1H), 6.93–6.84 (m, 1H), 6.11 (s, 1H), 5.91 (s, 1H), 5.13 (dd, *J* = 12.5, 4.6 Hz, 1H), 4.56 (dd, *J* = 12.9, 4.3 Hz, 1H), 4.34 (dtd, *J* = 40.7, 13.9, 3.5 Hz, 2H), 3.86 (s, 3H), 3.54 (s, 3H), 3.50–3.31 (m, 2H), 3.08–2.90 (m, 2H), 2.42 (d, *J* = 9.9 Hz, 6H).

^{13}C NMR (101 MHz, CD_3CN) δ 159.90, 157.98, 154.50, 150.91, 148.06, 146.10, 142.25, 142.02, 141.62, 140.48, 139.81, 139.77, 139.39, 139.32, 129.90, 128.75, 128.69, 126.86, 126.12, 124.83, 124.27, 124.19, 123.82, 123.38, 123.18, 122.77, 121.63, 120.55, 113.52, 112.67, 112.59, 90.60, 83.64, 52.66, 48.77, 34.16, 33.67, 27.20, 26.90, 16.63, 16.59.

MS (ESI+) m/z (%): 318 (100%, Z = 2)

HRMS (ESI+) m/z: [(M-2TfO$^-$)$^+$] ($C_{40}H_{36}N_4S_2$, Z = 2) calc.: 318.11852, found: 318.11880.

PR673.4.fid

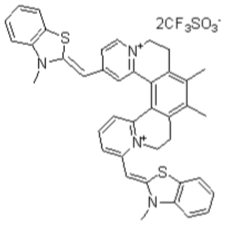

PR673

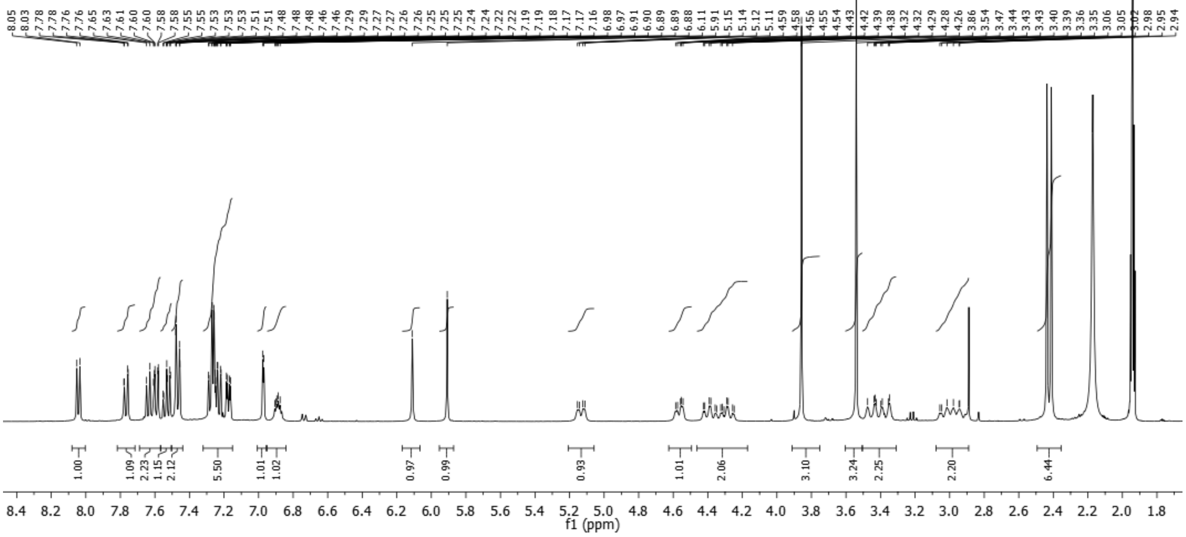

PR673.5.fid

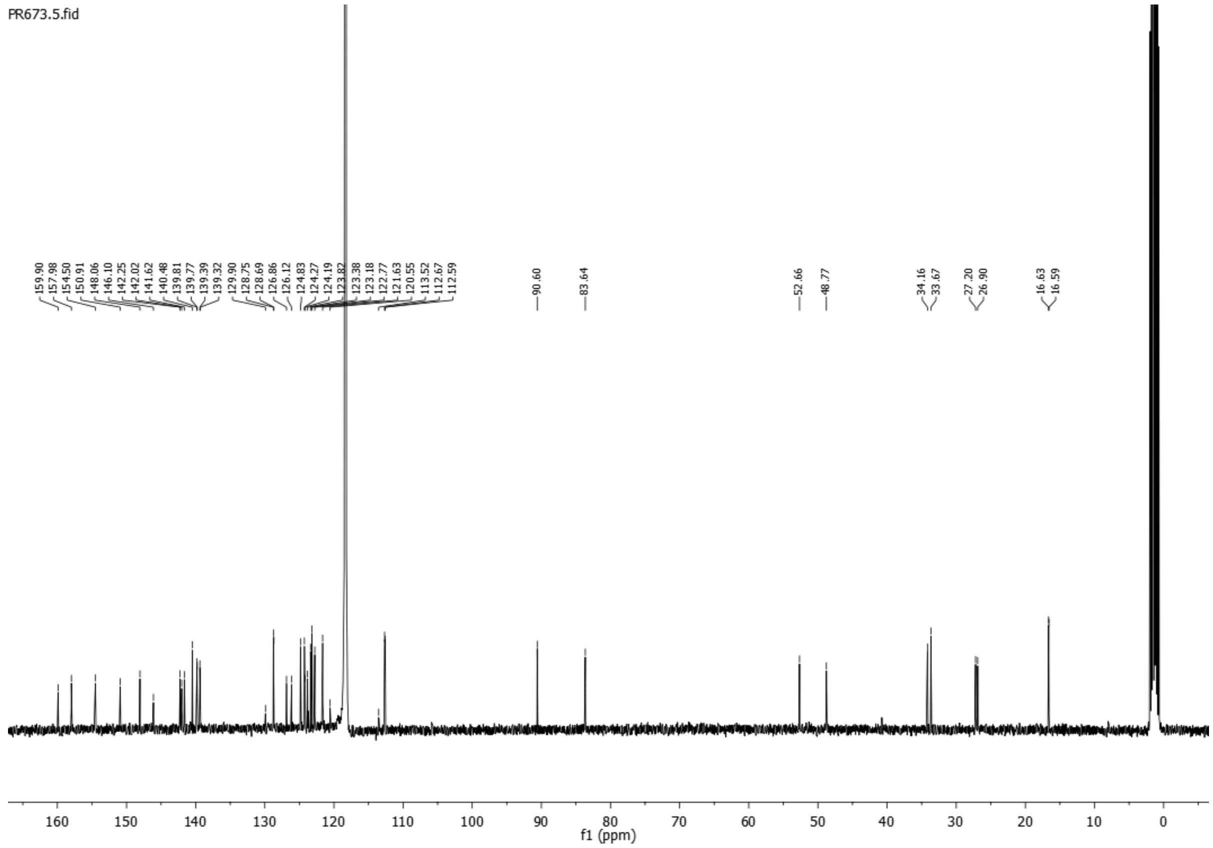

4 - VDJ930

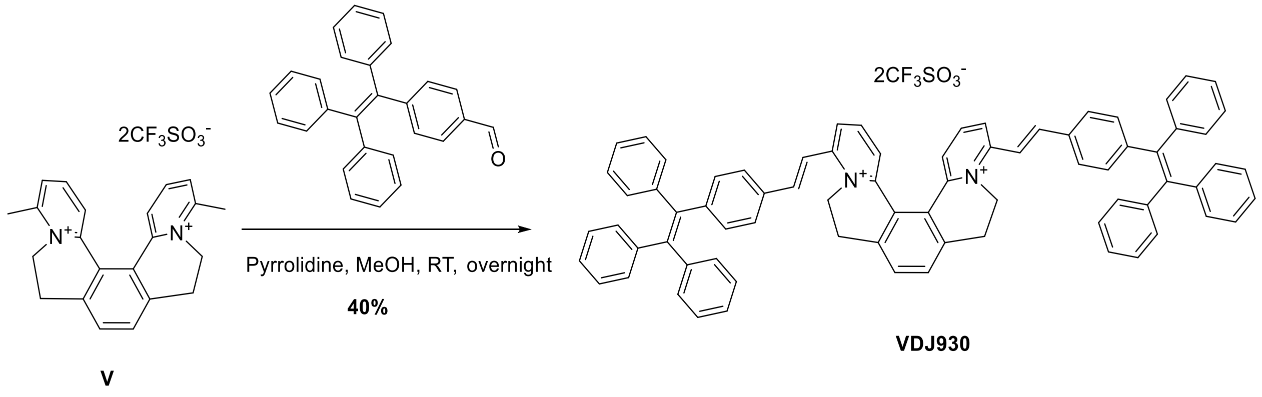

Helquat **V** (25 mg, 0.041 mmol), 4-(1,2,2-tryphenylvinyl)benzaldehyde (147 mg, 0.41 mmol, 10.0 equiv.), pyrrolidine (0.017 mL, 0.205 mmol, 5.0 equiv.) and MeOH (2.5 mL) were placed in a 10-mL flask and stirred under argon at room temperature overnight while being protected from ambient light using an aluminium-foil cover. The reaction progress was checked by TLC (mobile-phase Stoddart's magic mixture). The crude product was transferred to 50-mL centrifuge tubes and precipitated from the reaction mixture by the addition of Et_2O (20 mL to each tube). The resulting suspensions were centrifuged and supernatants were removed. Residues were dissolved in a minimum amount of MeOH (6.0 mL), after which Et_2O (40 mL) was added to each tube. Precipitates were centrifuged. This reprecipitation was repeated three more times. Centrifuged solids were collected in a glass vial using Et_2O. The resulting suspension was centrifuged and Et_2O was removed. The solids were dried under vacuum to obtain pure **VDJ930** as an orange solid in 40% yield (21 mg, 0.016 mmol).

^{1}H NMR (401 MHz, acetonitrile-d_3) δ 8.13–7.96 (m, 4H), 7.70 (d, *J* = 9.2 Hz, 4H), 7.63–7.46 (m, 8H), 7.26–7.03 (m, 34H), 5.19 (d, *J* = 13.3 Hz, 2H), 4.45 (t, *J* = 13.8 Hz, 2H), 3.35–3.04 (m, 4H).

^{13}C NMR (101 MHz, CD_3CN) δ 155.55, 147.94, 147.80, 144.50, 144.48, 144.38, 144.25, 143.93, 143.34, 141.28, 141.26, 133.87, 132.78, 132.62, 131.95, 131.92, 131.82, 129.59, 128.93, 128.92, 128.02, 127.87, 127.80, 127.78, 126.71, 118.74, 50.78, 28.04.

MS (ESI+) m/z (%): 499 (100%, Z = 2)

HRMS (ESI+) m/z: [(M-2TfO$^-$)$^+$] ($C_{76}H_{58}N_2$, Z = 2) calc.: 499.22945, found: 499.22951.

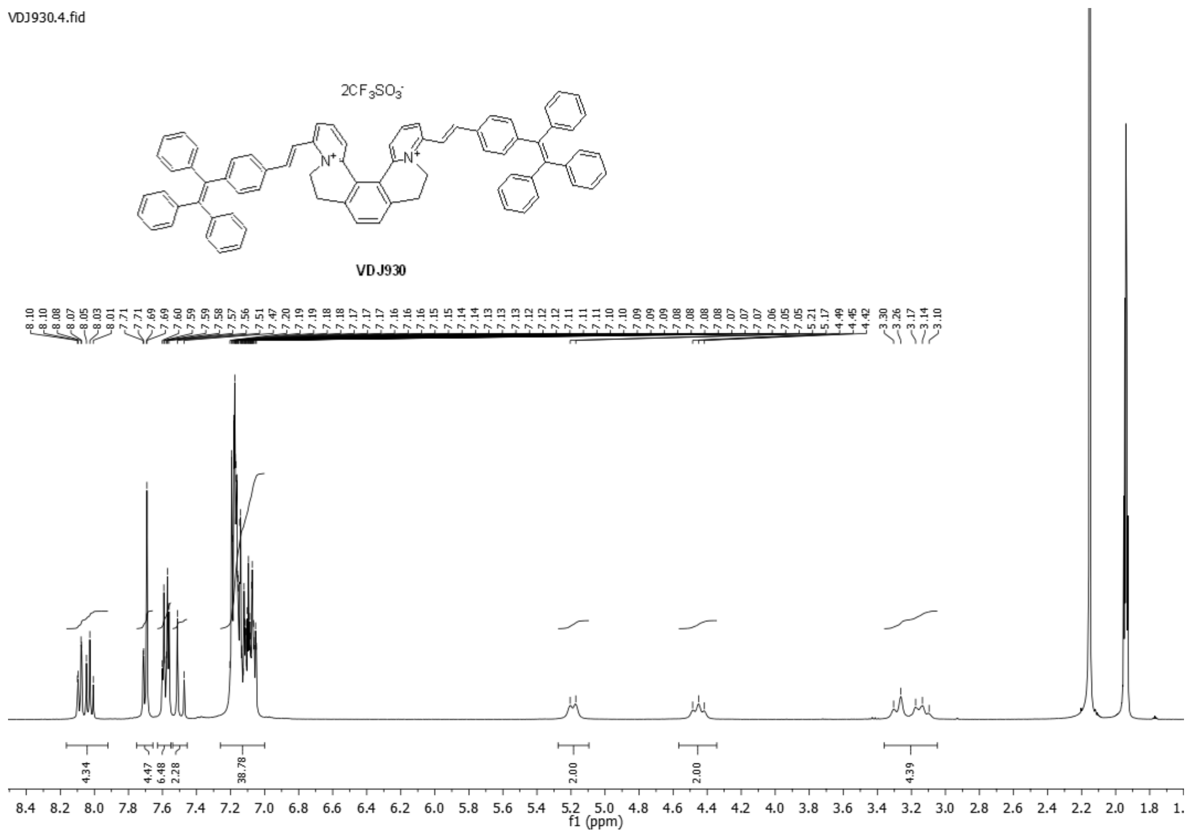
VDJ930.4.fid
2CF3SO3-
VDJ930
8.10 8.10 8.08 8.07 8.05 8.03 8.01 7.71 7.71 7.69 7.60 7.59 7.59 7.58 7.57 7.56 7.51 7.47 7.20 7.19 7.19 7.18 7.18 7.17 7.17 7.17 7.16 7.16 7.15 7.15 7.14 7.14 7.13 7.13 7.13 7.12 7.12 7.12 7.11 7.11 7.11 7.10 7.10 7.09 7.09 7.09 7.08 7.08 7.08 7.07 7.07 7.07 7.06 7.05 7.05 5.21 5.17 4.49 4.45 4.42 3.30 3.26 3.17 3.14 3.10
4.34 4.47 6.48 2.28 38.78 2.00 2.00 4.39
f1 (ppm)

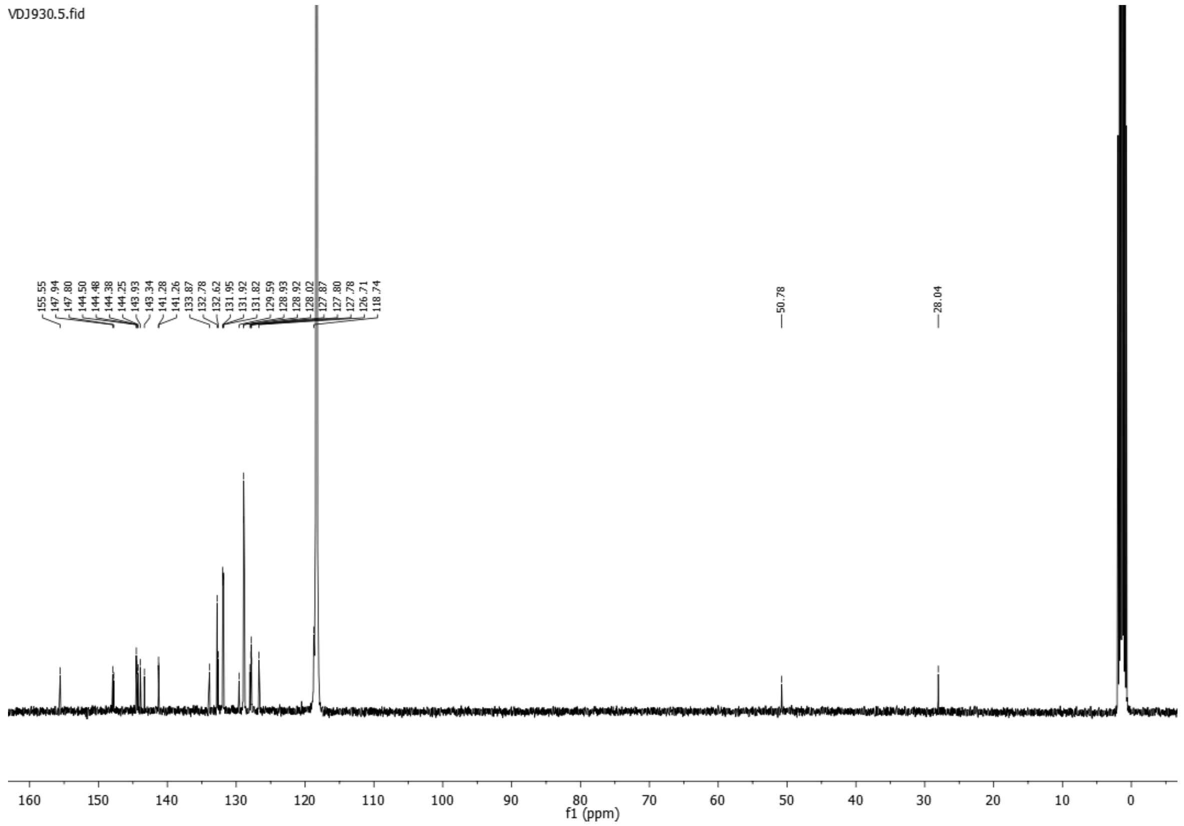
VDJ930.5.fid
155.55 147.94 147.80 144.50 144.48 144.38 144.25 143.93 143.34 141.28 141.26 133.87 132.78 132.62 131.95 131.92 131.82 129.59 128.93 128.92 128.02 127.87 127.80 127.78 126.71 118.74
50.78
28.04
f1 (ppm)

5 - PD576

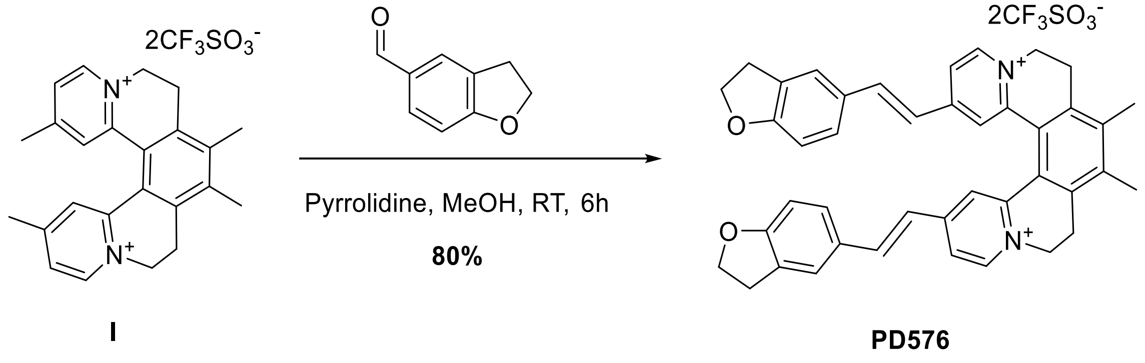

Helquat **I** (30 mg, 0.047 mmol), 2,3-dihydrobenzofuran-5-carbaldehyde (208 mg, 1.4 mmol, 30.0 equiv.), pyrrolidine (0.07 mL, 0.70 mmol, 15.0 equiv.) and MeOH (2.0 mL) were placed in a 10-mL flask and stirred under argon at room temperature for six hours while being protected from ambient light using an aluminium-foil cover. The reaction progress was checked by TLC (mobile-phase Stoddart's magic mixture). The crude product was transferred to 50-mL centrifuge tubes and precipitated from the reaction mixture by the addition of Et_2O (30 mL to each tube). The resulting suspensions were centrifuged and supernatants were removed. Residues were dissolved in a minimum amount of MeOH (1.0 mL), after which Et_2O (20 mL) was added to each tube. Precipitates were centrifuged. This reprecipitation was repeated three more times. Centrifuged solids were collected in a glass vial using Et_2O. The resulting suspension was centrifuged and Et_2O was removed. The solids were dried under vacuum to obtain pure **PD576** as a yellow solid in 80% yield (33 mg, 0.037mmol).

^{1}H NMR (401 MHz, acetonitrile-d_3) δ 8.57 (d, J = 6.6 Hz, 2H), 7.83 (dd, J = 6.7, 2.0 Hz, 2H), 7.77 (d, J = 1.9 Hz, 2H), 7.41 (d, J = 1.8 Hz, 2H), 7.33 (d, J = 16.2 Hz, 2H), 7.24 (dd, J = 8.3, 1.9 Hz, 2H), 6.90 (d, J = 16.2 Hz, 2H), 6.72 (d, J = 8.3 Hz, 2H), 4.83 (ddd, J = 13.3, 5.0, 1.7 Hz, 2H), 4.70 (td, J = 14.0, 3.8 Hz, 2H), 4.57 (t, J = 8.8 Hz, 4H), 3.43 (ddd, J = 17.4, 3.9, 1.8 Hz, 2H), 3.16 (dd, J = 9.7, 7.8 Hz, 4H), 3.05 (ddd, J = 17.8, 14.7, 5.0 Hz, 2H), 2.41 (s, 6H).

^{13}C NMR (101 MHz, CD_3CN) δ 163.77, 154.29, 147.97, 145.29, 142.35, 141.32, 140.14, 130.92, 130.14, 128.77, 127.25, 125.54, 125.30, 121.27, 120.32, 110.46, 73.12, 54.38, 29.65, 26.59, 16.96.

MS (ESI+) m/z (%): 301 (100%, Z = 2)

HRMS (ESI+) m/z: [$(M-2TfO^-)^+$] ($C_{42}H_{38}N_2O_2$, Z = 2) calc.: 301.14633, found: 301.14612.

PD576.1.fid

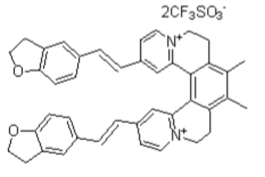

PD576

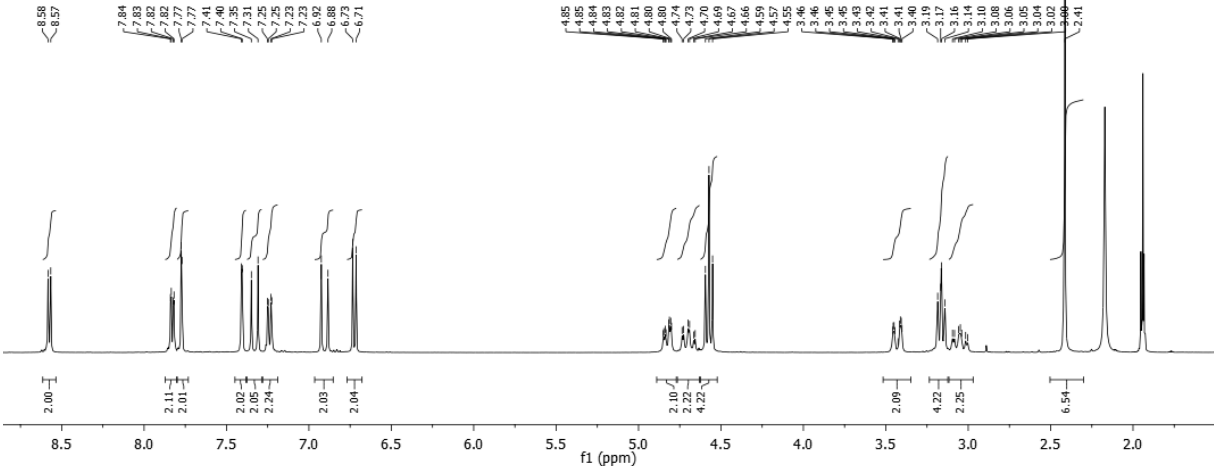

PD576.2.fid

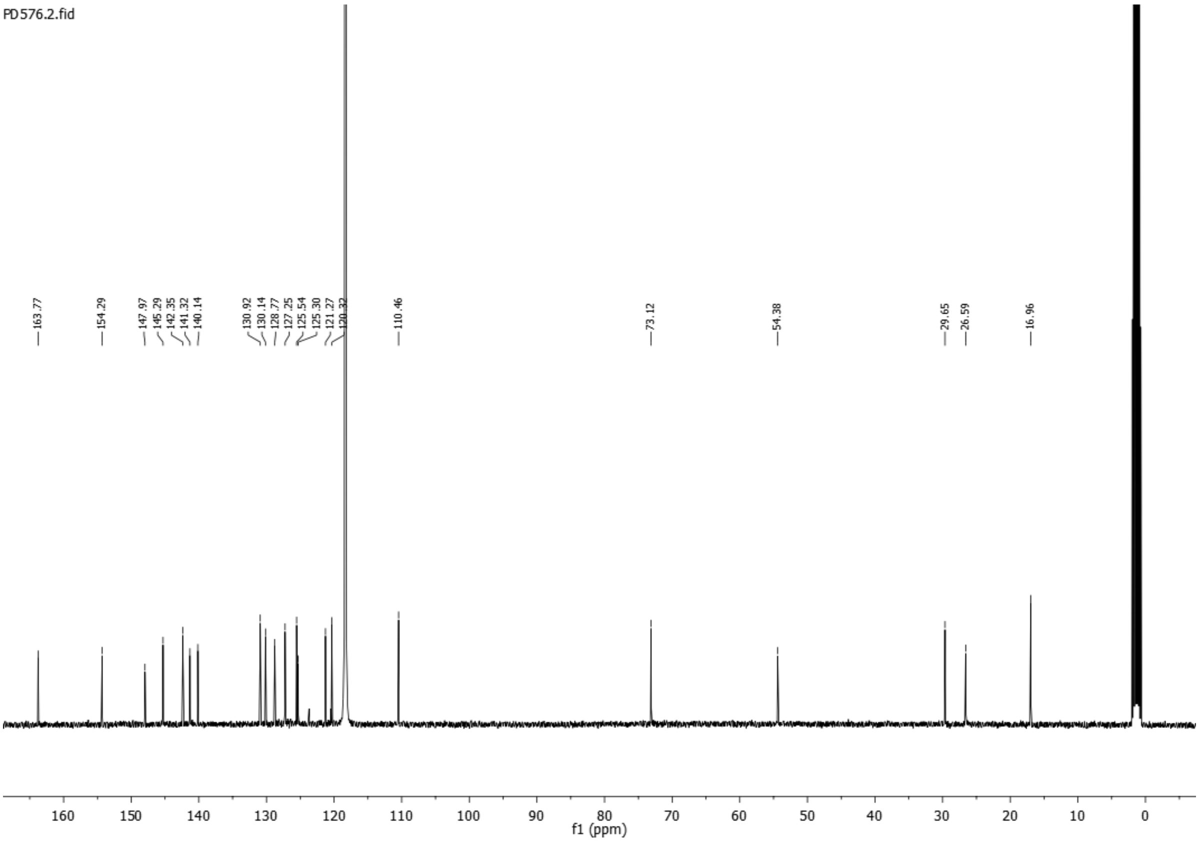

6 - PD275

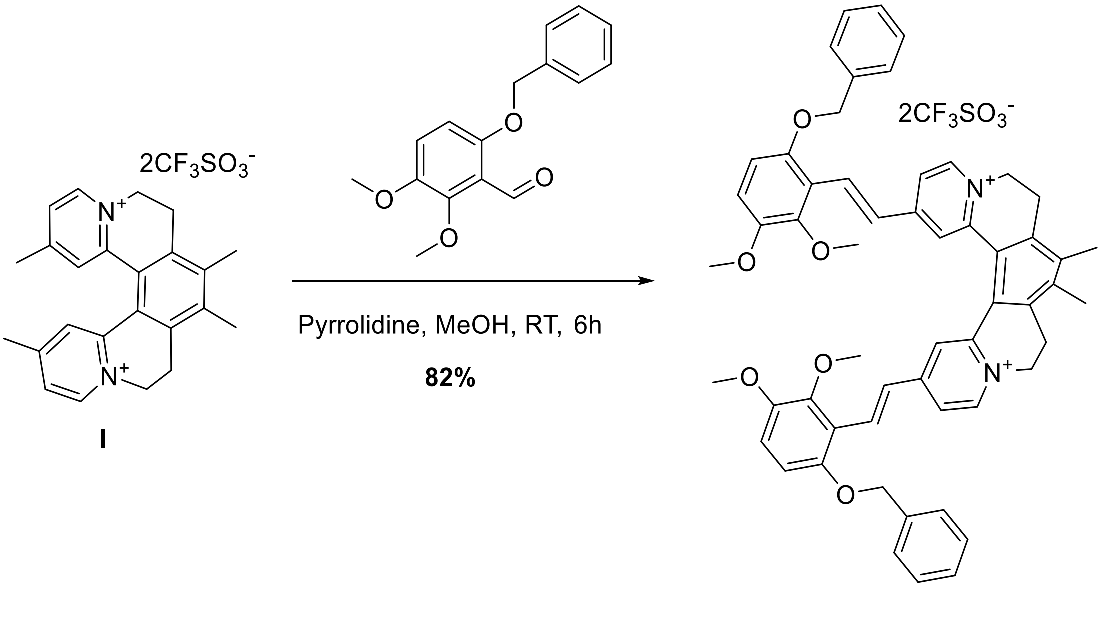

Helquat **I** (30 mg, 0.047 mmol), 6-(benzyloxy)-2,3-dimethoxybenzaldehyde (382 mg, 1.4 mmol, 30.0 equiv.), pyrrolidine (0.07 mL, 0.70 mmol, 15.0 equiv.) and MeOH (2.0 mL) were placed in a 10-mL flask and stirred 6h under argon at room temperature while being protected from ambient light using an aluminium-foil cover. The reaction progress was checked by TLC (mobile-phase Stoddart's magic mixture). Crude product was transferred to 50 mL centrifuge tubes and precipitated from The reaction mixture by addition of Et_2O (30 mL to each tube). The resulting suspensions were centrifuged and supernatants were removed. Residues were dissolved in a minimum amount of MeOH (1.0 mL), after which Et_2O (20 mL) was added to each tube. Precipitates were centrifuged. This reprecipitation was repeated three more times. Centrifuged solids were collected in a glass vial using Et_2O. The resulting suspension was centrifuged and Et_2O was removed. The solids were dried under vacuum to obtain pure **PD275** as a red solid in 82% yield (44 mg, 0.039 mmol).

^{1}H NMR (401 MHz, acetonitrile-d_3) δ 8.40 (d, J = 6.7 Hz, 2H), 7.81 (d, J = 1.9 Hz, 2H), 7.66 (dd, J = 6.7, 2.0 Hz, 2H), 7.59 (d, J = 16.3 Hz, 2H), 7.41–7.30 (m, 10H), 7.02–6.95 (m, 4H), 6.63 (s, 2H), 5.13 (d, J = 6.2 Hz, 4H), 4.81–4.60 (m, 4H), 3.77 (s, 6H), 3.71 (s, 6H), 3.47–3.38 (m, 2H), 3.03 (td, J = 17.6, 16.1, 5.0 Hz, 2H), 2.41 (s, 6H).

^{13}C NMR (101 MHz, CD3CN) δ 154.42, 154.04, 154.00, 147.86, 144.94, 144.89, 141.20, 139.97, 138.02, 136.70, 129.62, 129.09, 128.74, 127.13, 125.37, 121.24, 121.18, 116.89, 111.33, 100.40, 72.22, 56.90, 56.69, 54.25, 26.54, 16.96.

MS (ESI+) m/z (%): 425 (100%, Z = 2).

HRMS (ESI+) m/z: [(M-2TfO$^-$)$^+$] ($C_{56}H_{54}N_2O_6$, Z = 2) calc.: 425.19855, found: 425.19843.

PD275.4.fid

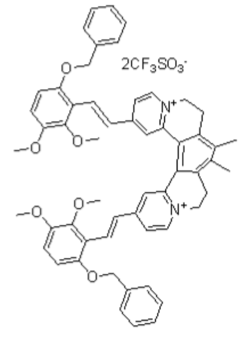

PD275

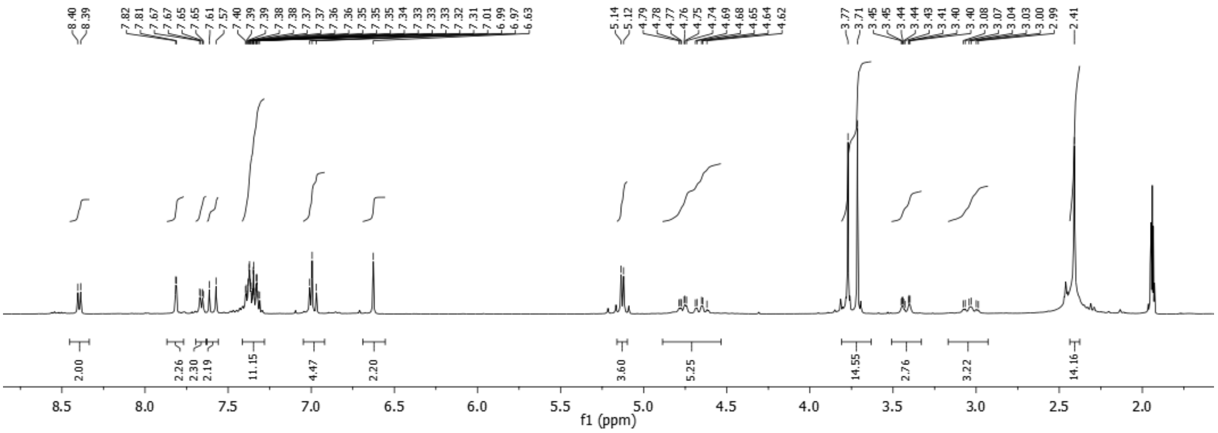

PD275.5.fid

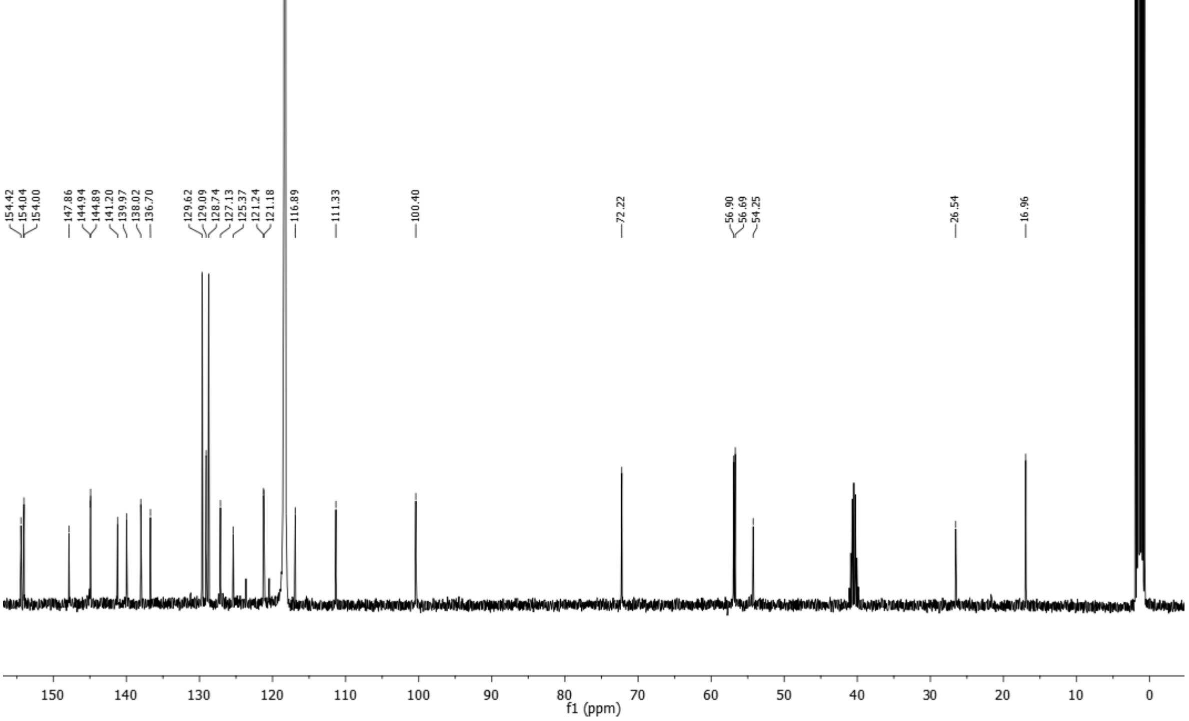

7 - LS1014

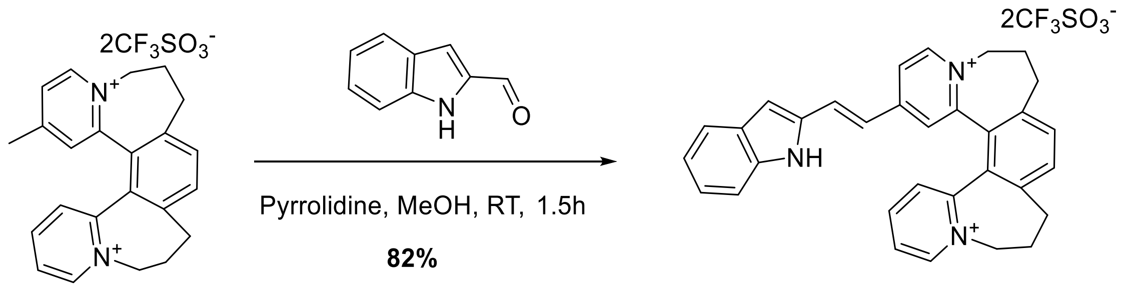

VI **LS1014**

Helquat **VI** (23 mg, 0.037 mmol), 1-H-indole-2-carbaldehyde (80 mg, 0.55 mmol, 15.0 equiv.), pyrrolidine (0.024 mL, 0.29 mmol, 8.0 equiv.) and MeOH (1.5 mL) were placed in a 10-mL flask and stirred overnight under argon at room temperature while being protected from ambient light using an aluminium-foil cover. The reaction progress was checked by TLC (mobile-phase Stoddart's magic mixture). Crude product was transferred to 50 mL centrifuge tubes and precipitated from The reaction mixture by addition of Et_2O (20 mL to each tube). The resulting suspensions were centrifuged and supernatants were removed. Residues were dissolved in a minimum amount of MeOH (1.0 mL), after which Et_2O (20 mL) was added to each tube. Precipitates were centrifuged. This reprecipitation was repeated three more times. Centrifuged solids were collected in a glass vial using Et_2O. The resulting suspension was centrifuged and Et_2O was removed. The solids were dried under vacuum to obtain pure **LS1014** as an orange solid in 82% yield (23 mg, 0.03 mmol).

^{1}H NMR (401 MHz, acetonitrile-d_3) δ 10.45 (s, 1H), 8.91 (dd, *J* = 6.2, 1.5 Hz, 1H), 8.59 (d, *J* = 6.7 Hz, 1H), 8.20 (td, *J* = 7.9, 1.5 Hz, 1H), 8.00 (dd, *J* = 6.8, 2.1 Hz, 1H), 7.92 (ddd, *J* = 7.7, 6.1, 1.5 Hz, 1H), 7.69–7.62 (m, 3H), 7.59 (d, *J* = 8.0 Hz, 1H), 7.36 (dt, *J* = 8.3, 0.9 Hz, 2H), 7.23 (ddd, *J* = 8.2, 7.0, 1.1 Hz, 1H), 7.13 (d, *J* = 2.1 Hz, 1H), 7.10–7.03 (m, 2H), 6.87 (dd, *J* = 1.9, 0.9 Hz, 1H), 4.87 (dd, *J* = 13.8, 6.3 Hz, 1H), 4.60 (dt, *J* = 12.5, 6.2 Hz, 2H), 4.38 (td, *J* = 13.3, 5.7 Hz, 1H), 2.97 (dd, *J* = 13.8, 6.2 Hz, 2H), 2.63 (dq, *J* = 20.1, 6.6 Hz, 2H), 2.52–2.28 (m, 4H).

^{13}C NMR (101 MHz, CD_3CN) δ 154.93, 152.82, 152.23, 147.82, 146.18, 145.87, 140.13, 140.02, 139.97, 135.81, 134.30, 134.17, 132.73, 132.34, 131.42, 130.99, 129.34, 128.79, 128.42, 126.08, 122.47, 121.92, 121.43, 121.06, 112.54, 111.20, 59.66, 57.63, 32.47, 32.14, 29.30, 29.27.

MS (ESI+) m/z (%): 603 (M-TfO$^-$, 100%)

HRMS (ESI+) m/z: [(M-2TfO$^-$)$^+$] ($C_{32}H_{29}N_3$, Z = 2) calc.: 227.61753, found: 227.61762.

LS1014.4.fid

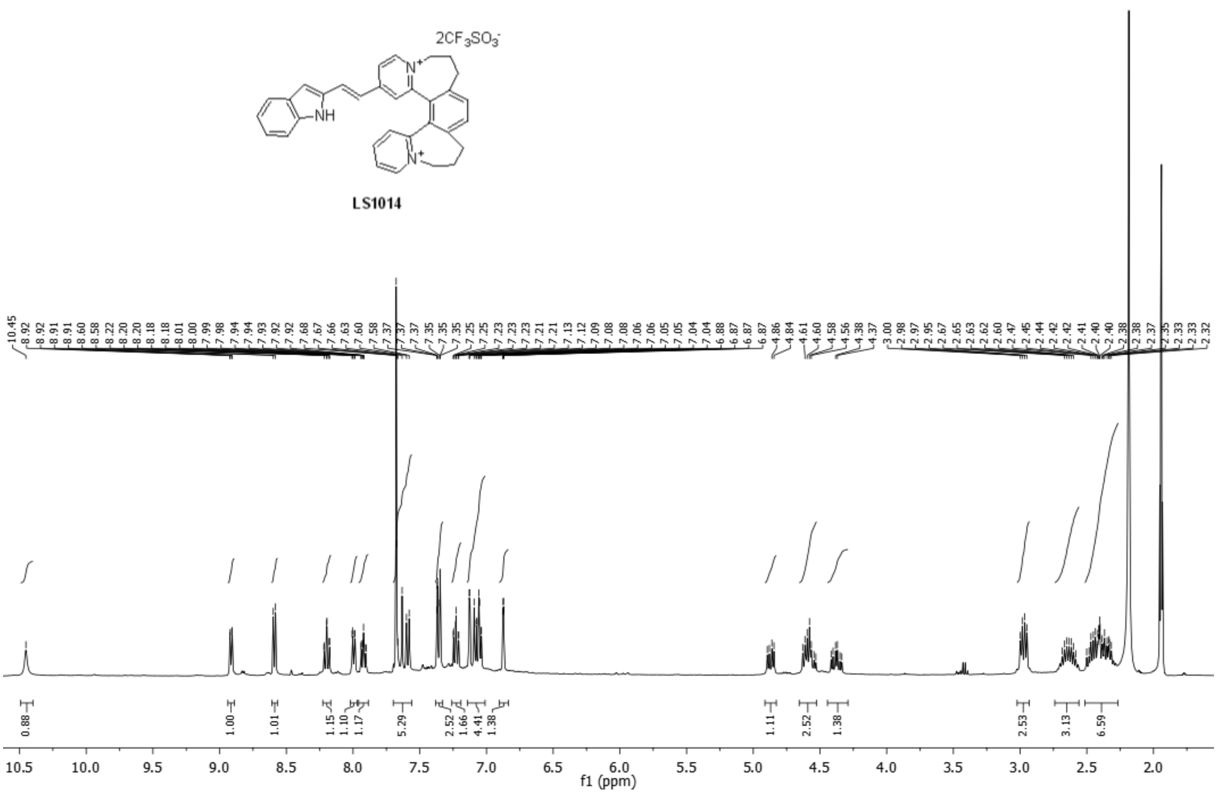

LS1014.5.fid

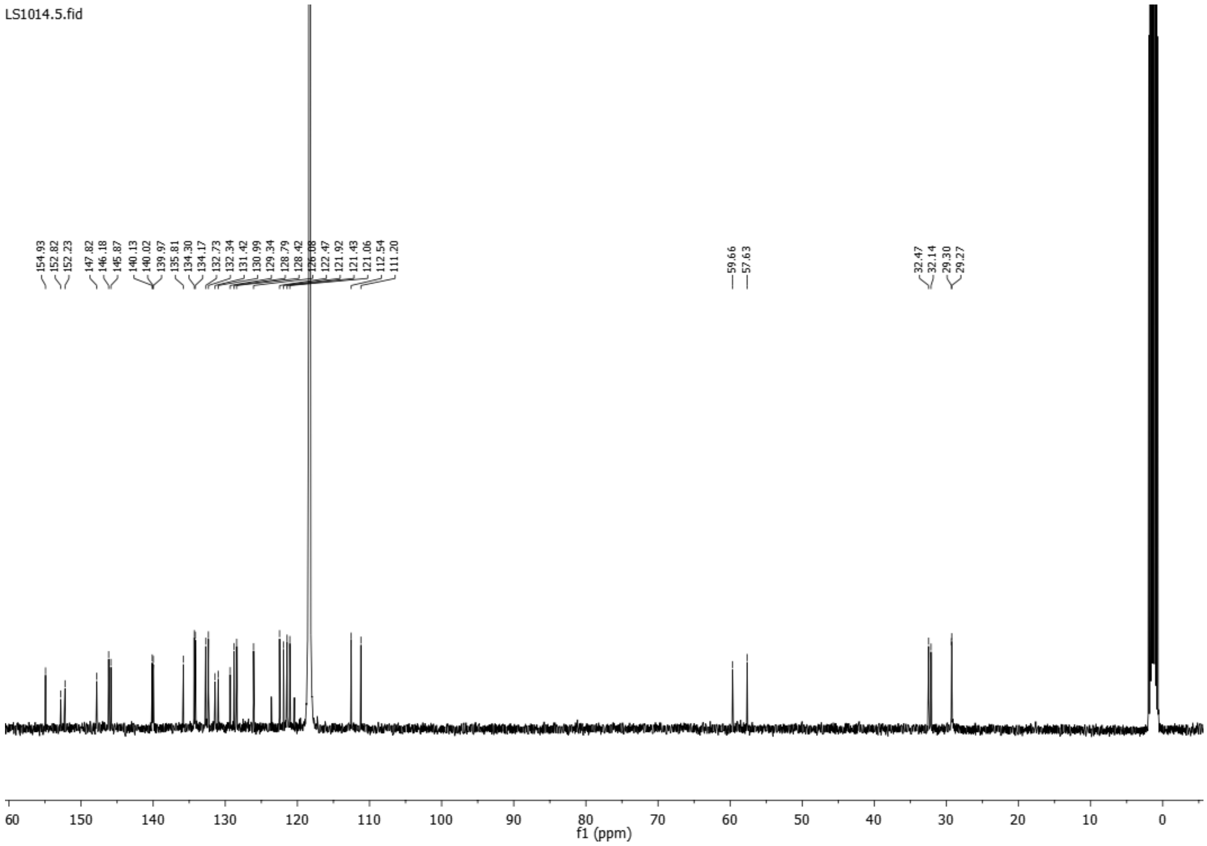

8 - PD211

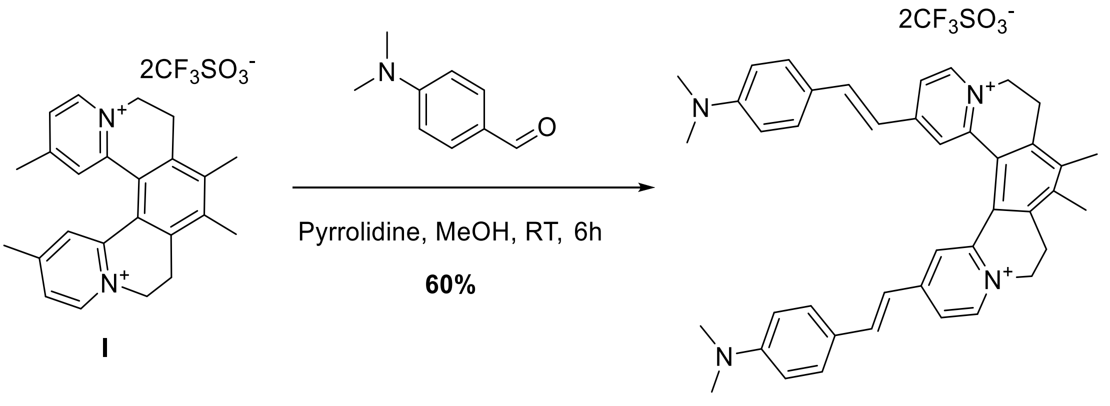

Helquat **I** (30 mg, 0.047 mmol), 4-(dimethylamino)benzaldehyde (189 mL, 1.4 mmol, 30.0 equiv.), pyrrolidine (0.07 mL, 0.70 mmol, 15.0 equiv.) and MeOH (2.0 mL) were placed in a 10-mL flask and stirred under argon at room temperature for six hours while being protected from ambient light using an aluminium-foil cover. The reaction progress was checked by TLC (mobile-phase Stoddart's magic mixture). Crude product was transferred to 50 mL centrifuge tubes and precipitated from The reaction mixture by addition of Et_2O (30 mL to each tube). The resulting suspensions were centrifuged and supernatants were removed. Residues were dissolved in a minimum amount of MeOH (1.0 mL), after which Et_2O (20 mL) was added to each tube. Precipitates were centrifuged. This reprecipitation was repeated three more times. Centrifuged solids were collected in a glass vial using Et_2O. The resulting suspension was centrifuged and Et_2O was removed. The solids were dried under vacuum to obtain pure **PD211** as a purple solid in 60% yield (25 mg, 0.03 mmol).

^{1}H NMR (401 MHz, acetonitrile-*d*$_3$) δ 8.47 (d, *J* = 6.7 Hz, 2H), 7.74 (dd, *J* = 6.7, 2.1 Hz, 2H), 7.66 (d, *J* = 2.0 Hz, 2H), 7.40–7.28 (m, 5H), 6.79 (d, *J* = 16.0 Hz, 2H), 6.72–6.66 (m, 4H), 4.78 (dd, *J* = 13.5, 4.3 Hz, 2H), 4.62 (td, *J* = 13.9, 3.6 Hz, 2H), 3.48–3.37 (m, 2H), 2.99 (s, 15H), 2.42 (s, 6H).

^{13}C NMR (101 MHz, CD_3CN) δ 154.63, 153.44, 147.64, 144.76, 143.12, 141.07, 140.00, 131.12, 126.46, 125.42, 123.34, 120.60, 117.51, 112.92, 54.08, 40.29, 26.69, 16.92.

MS (ESI+) m/z (%): 302 (100%, Z = 2).

HRMS (ESI+) m/z: [(M-2TfO$^-$)$^+$] ($C_{42}H_{44}N_4$, Z = 2) calc.: 302.17775, found: 302.117802.

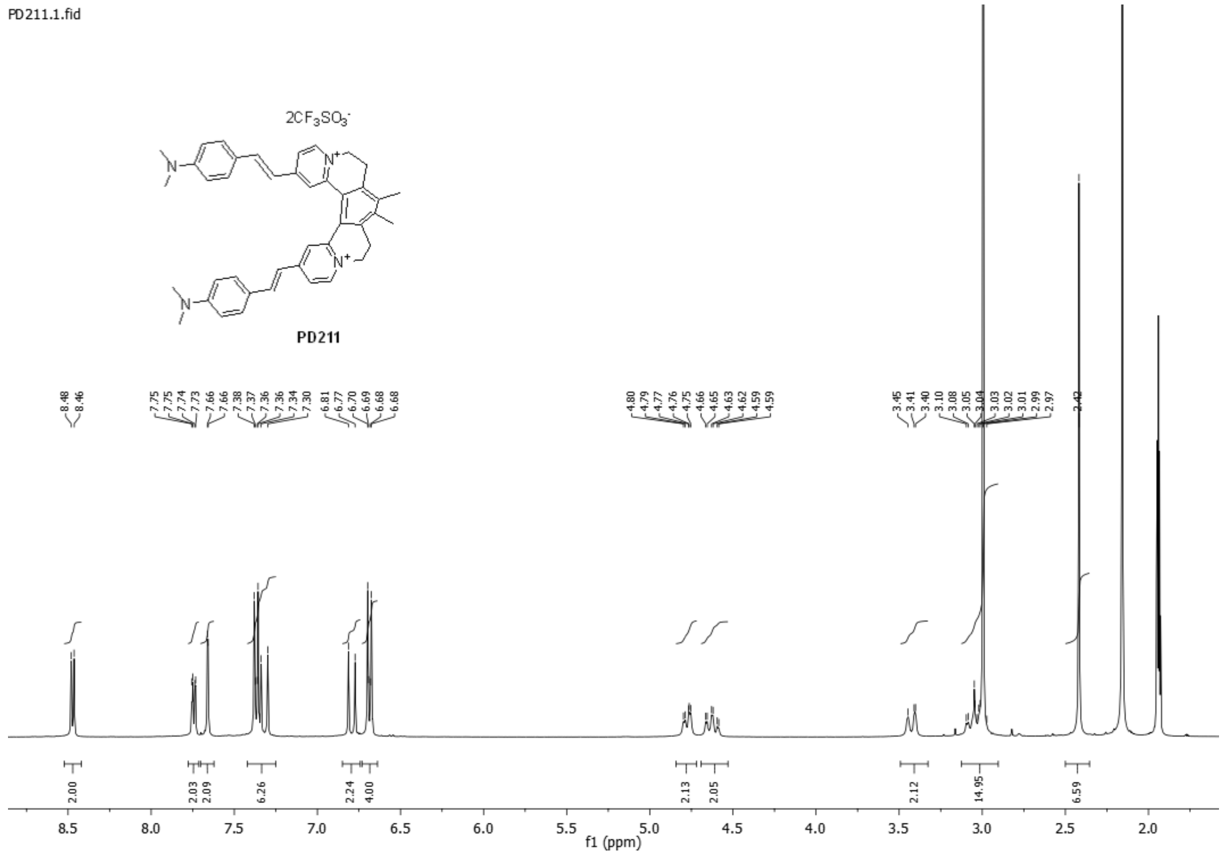
PD211.1.fid
2CF3SO3-
PD211
8.48 8.46
7.75 7.75 7.74 7.73 7.66 7.66 7.38 7.37 7.36 7.36 7.34 7.30 6.81 6.77 6.70 6.69 6.68 6.68
4.80 4.79 4.77 4.76 4.75 4.66 4.65 4.63 4.62 4.59 4.59
3.45 3.41 3.40 3.10 3.08 3.05 3.04 3.03 3.02 3.01 2.99 2.97
2.42
2.00 2.03 2.09 6.26 2.24 4.00 2.13 2.05 2.12 14.95 6.59
8.5 8.0 7.5 7.0 6.5 6.0 5.5 5.0 4.5 4.0 3.5 3.0 2.5 2.0
f1 (ppm)

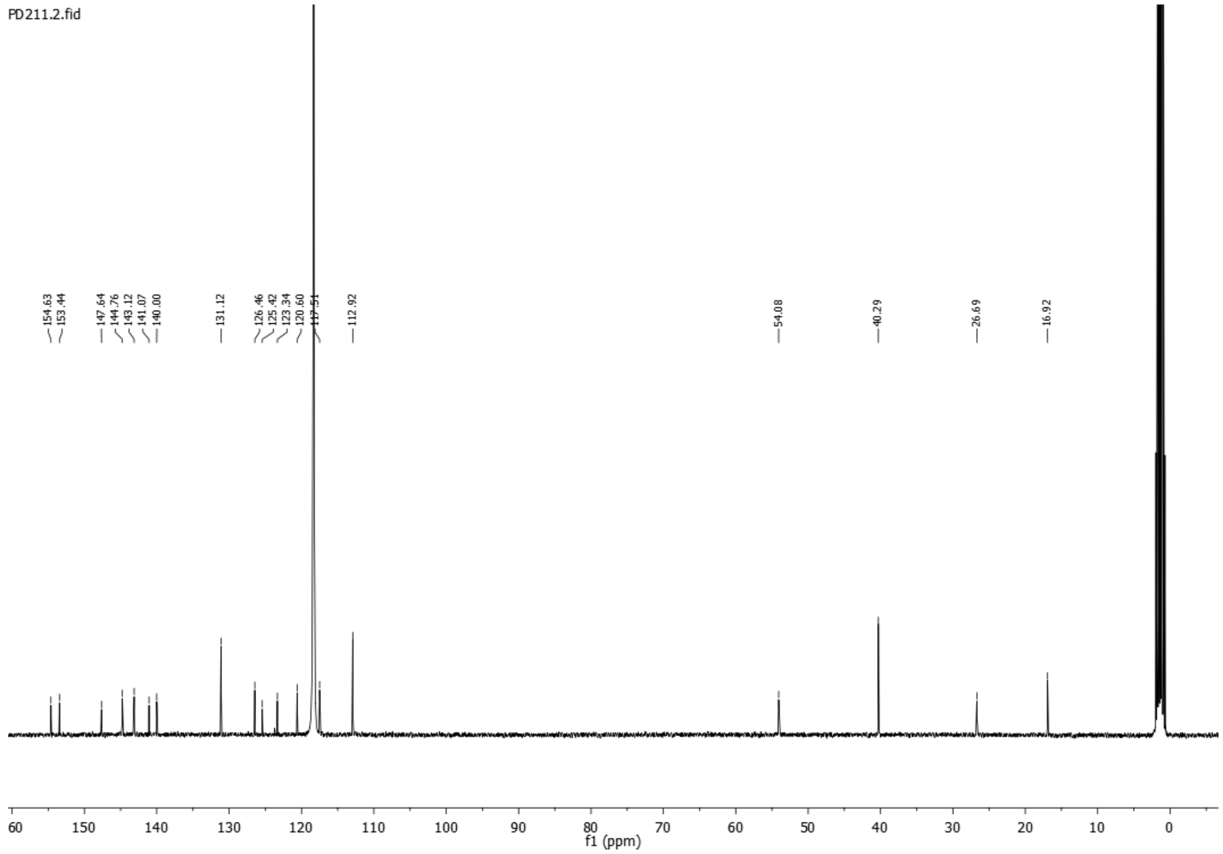
PD211.2.fid
154.63 153.44 147.64 144.76 143.12 141.07 140.00 131.12 126.46 125.42 123.34 120.60 117.51 112.92
54.08 40.29 26.69 16.92
60 150 140 130 120 110 100 90 80 70 60 50 40 30 20 10 0
f1 (ppm)

9 - VDJ820-2

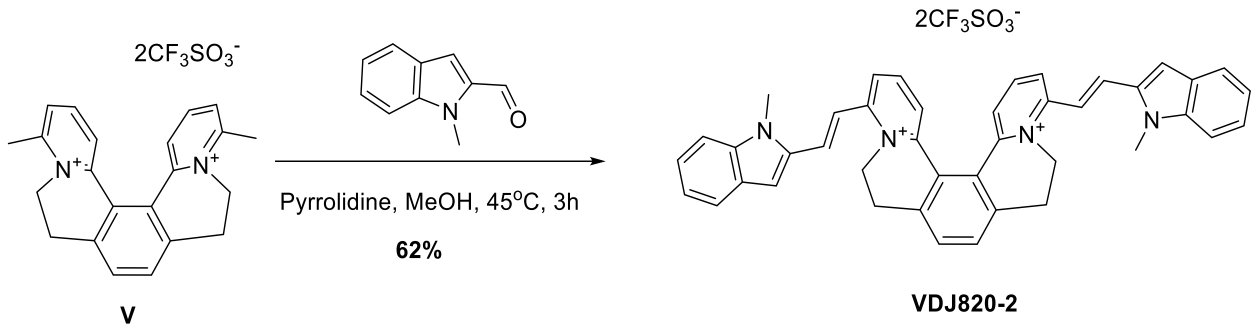

Helquat **V** (20 mg, 0.033 mmol), 1-methyl-1H-indole-2-carbaldehyde (70 mg, 0.33 mmol, 10.0 equiv.), pyrrolidine (0.014 mL, 0.165 mmol, 5.0 equiv.) and MeOH (2.0 mL) were placed in a 10-mL flask and stirred under argon at 45°C for three hours while being protected from ambient light using an aluminium-foil cover. The reaction progress was checked by TLC (mobile-phase Stoddart's magic mixture). The crude product was transferred to 50-mL centrifuge tubes and precipitated from the reaction mixture by the addition of Et_2O (16 mL to each tube). The resulting suspensions were centrifuged and supernatants were removed. Residues were dissolved in a minimum amount of MeOH (2.0 mL), after which Et_2O (20 mL) was added to each tube. Precipitates were centrifuged. This reprecipitation was repeated three more times. Centrifuged solids were collected in a glass vial using Et_2O. The resulting suspension was centrifuged and Et_2O was removed. The solids were dried under vacuum to obtain pure **VDJ820-2** as an orange solid in 62% yield (18 mg, 0.020 mmol).

^{1}H NMR (401 MHz, DMSO-d_6) δ 8.58 (d, J = 8.2 Hz, 2H), 8.22–8.00 (m, 6H), 7.91–7.77 (m, 4H), 7.68 (d, J = 8.0 Hz, 2H), 7.58 (d, J = 9.8 Hz, 4H), 7.29 (t, J = 7.7 Hz, 2H), 7.13 (t, J = 7.5 Hz, 2H), 5.46–5.35 (m, 2H), 4.53 (td, J = 13.8, 3.5 Hz, 2H), 3.99 (s, 6H), 3.43–3.35 (m, 2H), 3.29–3.17 (m, 2H).

^{13}C NMR (101 MHz, DMSO) δ 153.06, 146.58, 141.84, 139.90, 138.97, 136.01, 131.59, 131.08, 127.91, 127.16, 127.11, 124.96, 123.77, 121.17, 120.52, 118.18, 110.55, 104.03, 64.92, 49.21, 29.94.

MS (ESI+) m/z (%): 298 (100%, Z = 2)

HRMS (ESI+) m/z: [(M-2TfO$^-$)$^+$] ($C_{42}H_{36}N_4$, Z = 2) calc.: 298.14645, found: 298.14673.

VDJ820-2.4.fid

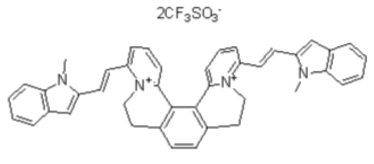

VDJ820-2

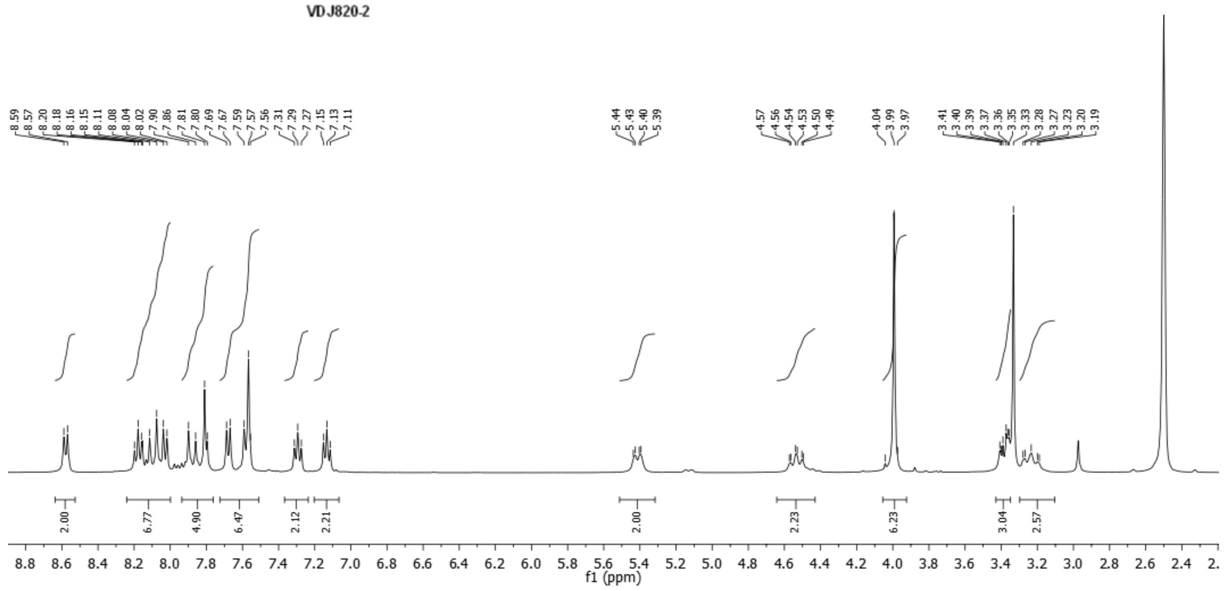

VDJ820-2.5.fid

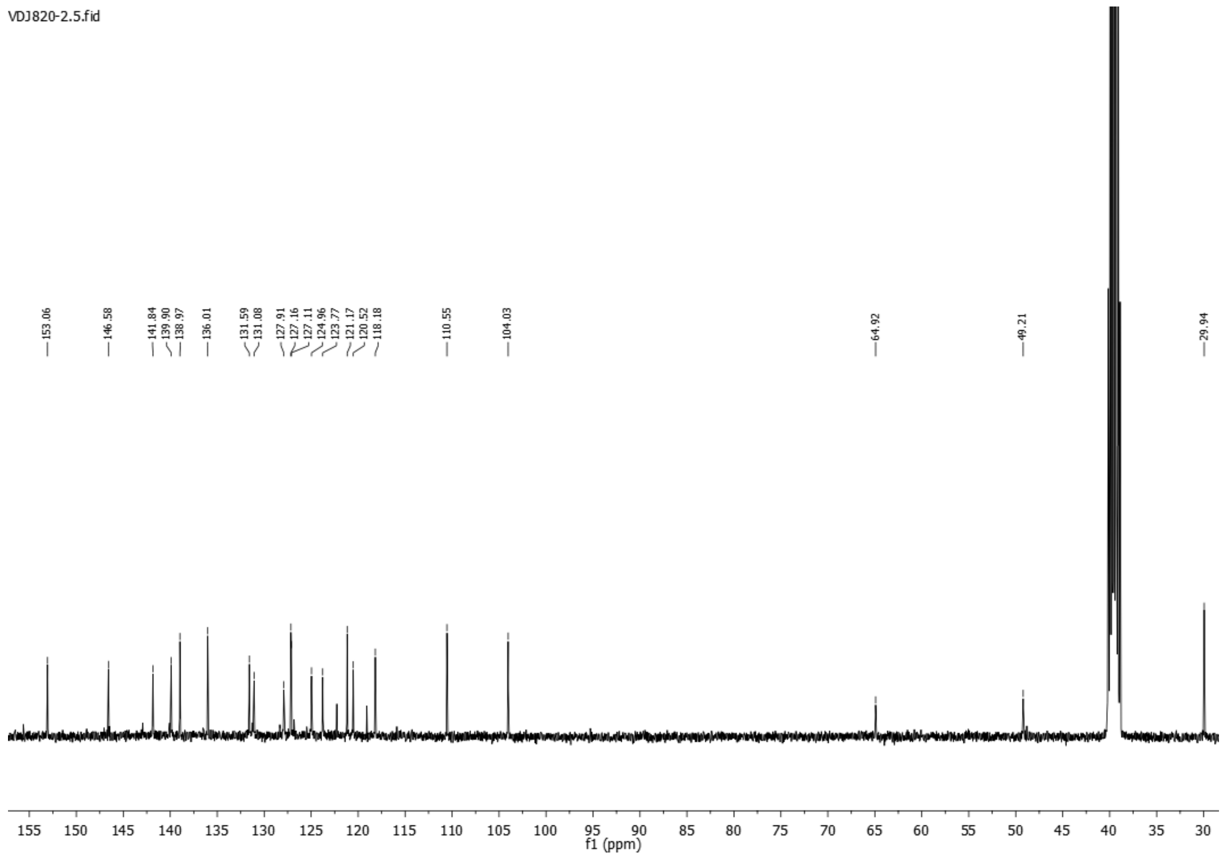

10 - PR606

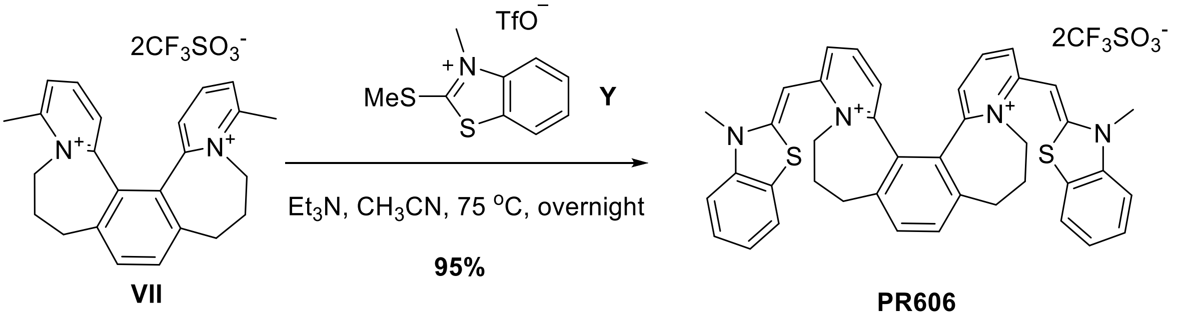

Helquat **VII** (30 mg, 0.047 mmol), **Y** (131 mg, 0.38 mmol, 8.0 equiv.), Et_3N (0.052 mL, 0.038 mmol) and CH_3CN (1.5 mL) were placed in a 10-mL flask and stirred under argon at 75 °C overnight while being protected from ambient light using an aluminium-foil cover. The reaction progress was checked by TLC (mobile-phase Stoddart's magic mixture). The crude product was transferred to 50-mL centrifuge tubes (3 tubes with 0.5 mL of reaction crude each) and precipitated from the reaction mixture by the addition of Et_2O (25 mL to each tube). The resulting suspensions were centrifuged and supernatants were removed. Residues were dissolved in a minimum amount of CH_3CN (0.5 mL added to each tube), after which Et_2O (25 mL) was added to each tube. Precipitates were centrifuged. This reprecipitation was repeated twice. Centrifuged solids were collected in a glass vial using Et_2O. The resulting suspension was centrifuged and Et_2O was removed. The solids were dried under vacuum to obtain pure **PR606** as a light-red solid in 95% yield (42 mg, 0.045 mmol).

^1H NMR (401 MHz, acetonitrile-*d*$_3$) δ 7.99 (dd, *J* = 8.8, 1.2 Hz, 2H), 7.81–7.69 (m, 4H), 7.61 (s, 2H), 7.54 (ddd, *J* = 8.4, 7.3, 1.2 Hz, 2H), 7.45 (dt, *J* = 8.4, 0.9 Hz, 2H), 7.32 (ddd, *J* = 8.3, 7.3, 1.1 Hz, 2H), 6.60 (dd, *J* = 7.5, 1.2 Hz, 2H), 5.85 (s, 2H), 4.78 (dd, *J* = 14.7, 5.6 Hz, 2H), 4.23–4.05 (m, 2H), 3.76 (s, 6H), 3.08–2.93 (m, 2H), 2.68 (dt, *J* = 13.6, 7.0 Hz, 2H), 2.44 (td, *J* = 13.5, 7.1 Hz, 2H), 2.26 (ddd, *J* = 13.1, 7.3, 5.4 Hz, 3H).

^{13}C NMR (101 MHz, CD_3CN) δ 160.36, 154.29, 152.67, 142.27, 141.13, 139.32, 133.09, 132.97, 128.83, 124.96, 123.91, 123.31, 122.73, 122.24, 118.09, 112.80, 83.19, 51.95, 34.14, 29.71, 29.66.

MS (ESI+) m/z (%): 318 (100%, Z = 2)

HRMS (ESI+) m/z: [$(M-2TfO^-)^+$] ($C_{40}H_{36}N_4S_2$, Z = 2) calc.: 318.11852, found: 318.11877.

PR606.2.fid

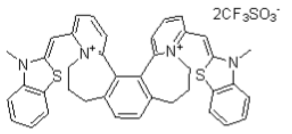

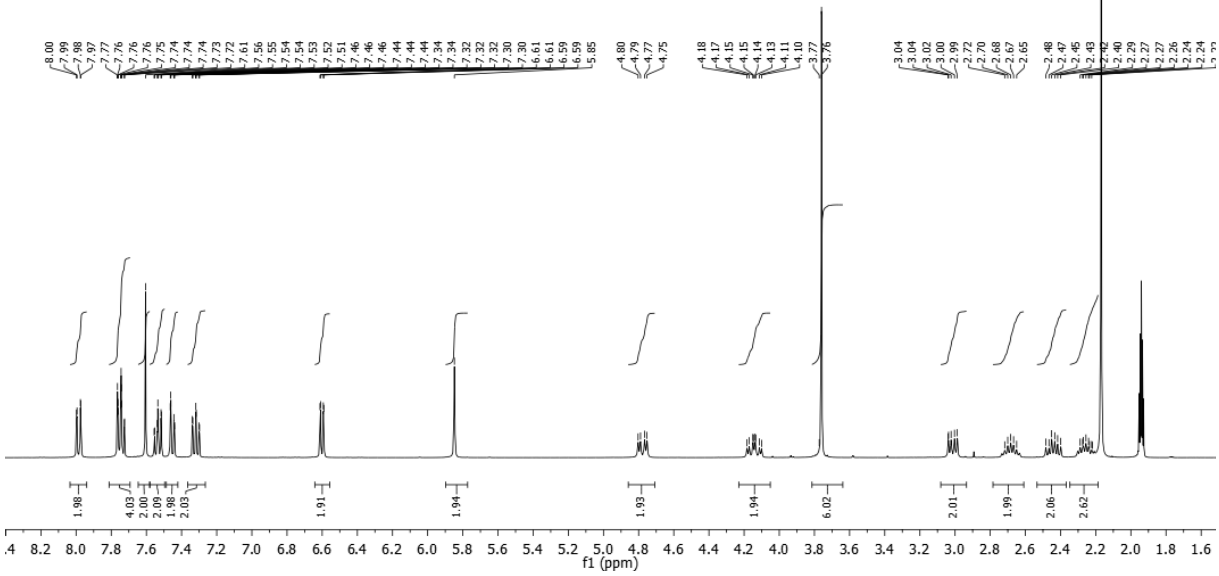

PR606.3.fid

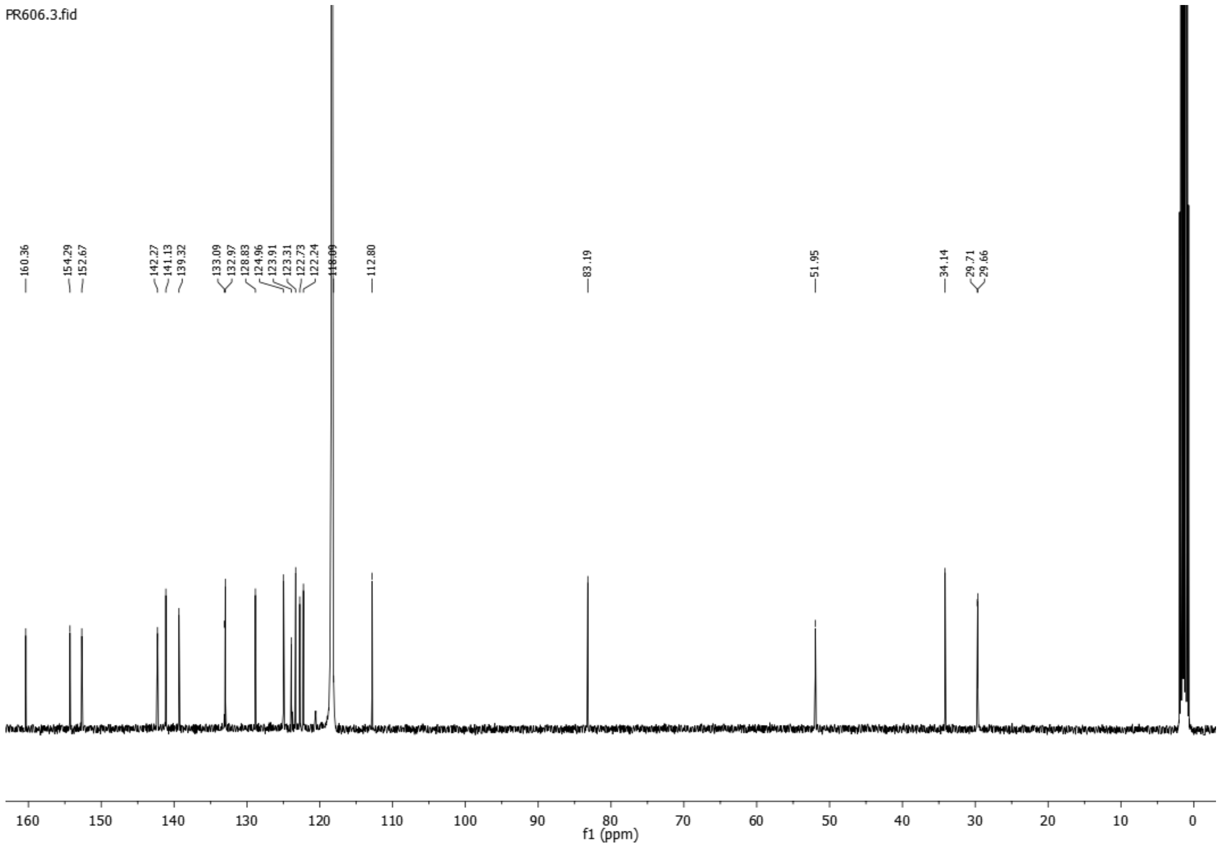

11 - PD313

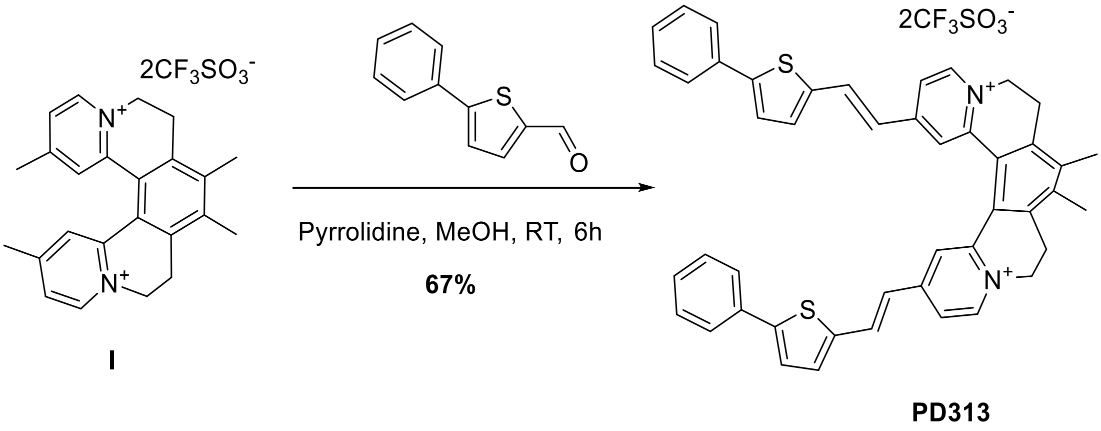

Helquat **I** (30 mg, 0.047 mmol), 5-phenylthiophene-2-carbaldehyde (264 mg, 1.4 mmol, 30.0 equiv.), pyrrolidine (0.07 mL, 0.70 mmol, 15.0 equiv.) and MeOH (2.0 mL) were placed in a 10-mL flask and stirred under argon at room temperature for six hours while being protected from ambient light using an aluminium-foil cover. The reaction progress was checked by TLC (mobile-phase Stoddart's magic mixture). The crude product was transferred to 50-mL centrifuge tubes and precipitated from the reaction mixture by the addition of Et_2O (30 mL to each tube). The resulting suspensions were centrifuged and supernatants were removed. Residues were dissolved in a minimum amount of MeOH (1.0 mL), after which Et_2O (20 mL) was added to each tube. Precipitates were centrifuged. This reprecipitation was repeated three more times. Centrifuged solids were collected in a glass vial using Et_2O. The resulting suspension was centrifuged and Et_2O was removed. The solids were dried under vacuum to obtain pure **PD313** as an orange solid in 67% yield (31 mg, 0.032 mmol).

^{1}H NMR (401 MHz, acetonitrile-*d*$_3$) δ 8.62 (d, *J* = 6.7 Hz, 2H), 7.88 (dd, *J* = 6.6, 2.0 Hz, 2H), 7.74–7.58 (m, 8H), 7.48–7.38 (m, 8H), 7.31 (d, *J* = 3.9 Hz, 2H), 6.84 (d, *J* = 16.0 Hz, 2H), 4.91–4.84 (m, 2H), 4.69 (td, *J* = 14.0, 13.6, 3.8 Hz, 2H), 3.51–3.43 (m, 2H), 3.08 (td, *J* = 17.6, 16.2, 4.8 Hz, 2H), 2.44 (s, 6H).

^{13}C NMR (101 MHz, CD_3CN) δ 153.52, 149.09, 148.08, 145.38, 141.48, 140.57, 140.29, 134.73, 134.50, 134.10, 130.25, 129.84, 127.43, 126.77, 125.89, 125.22, 122.05, 121.31, 54.48, 26.60, 16.97.

MS (ESI+) m/z (%): 341 (100%, M-2TfO$^-$,Z = 2), 681 (15%, M-2TfO$^-$), 831 (10%, M-TfO$^-$)

HRMS (ESI+) m/z: [(M-2TfO$^-$)$^+$] ($C_{46}H_{38}N_2S_2$, Z = 2) calc.: 341.12321, found: 341.12372.

PD313.3.fid

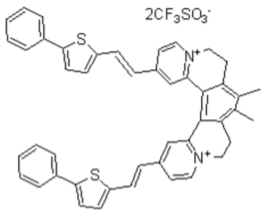

PD313

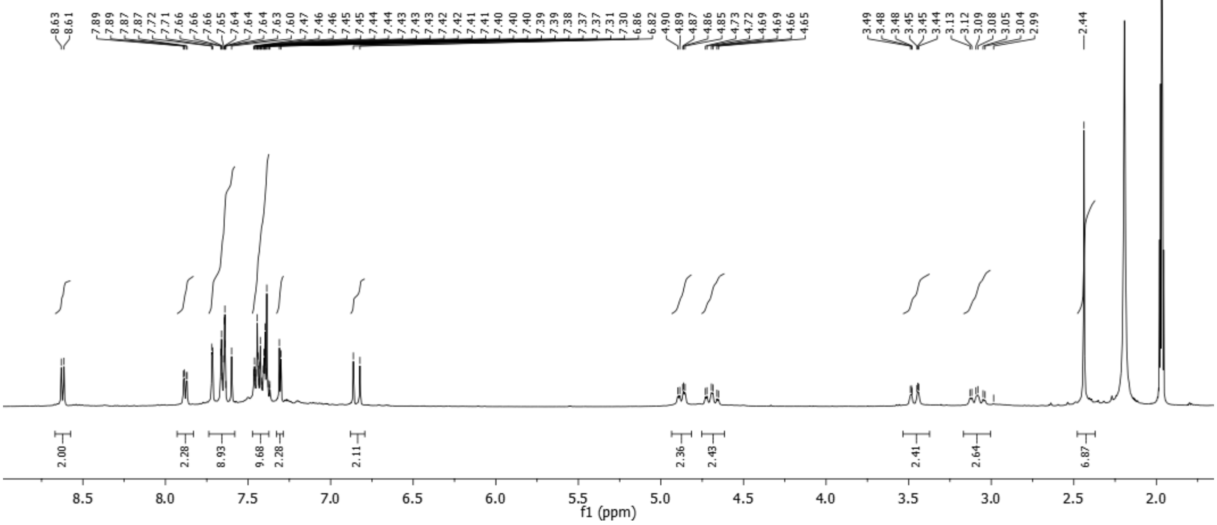

PD313.4.fid

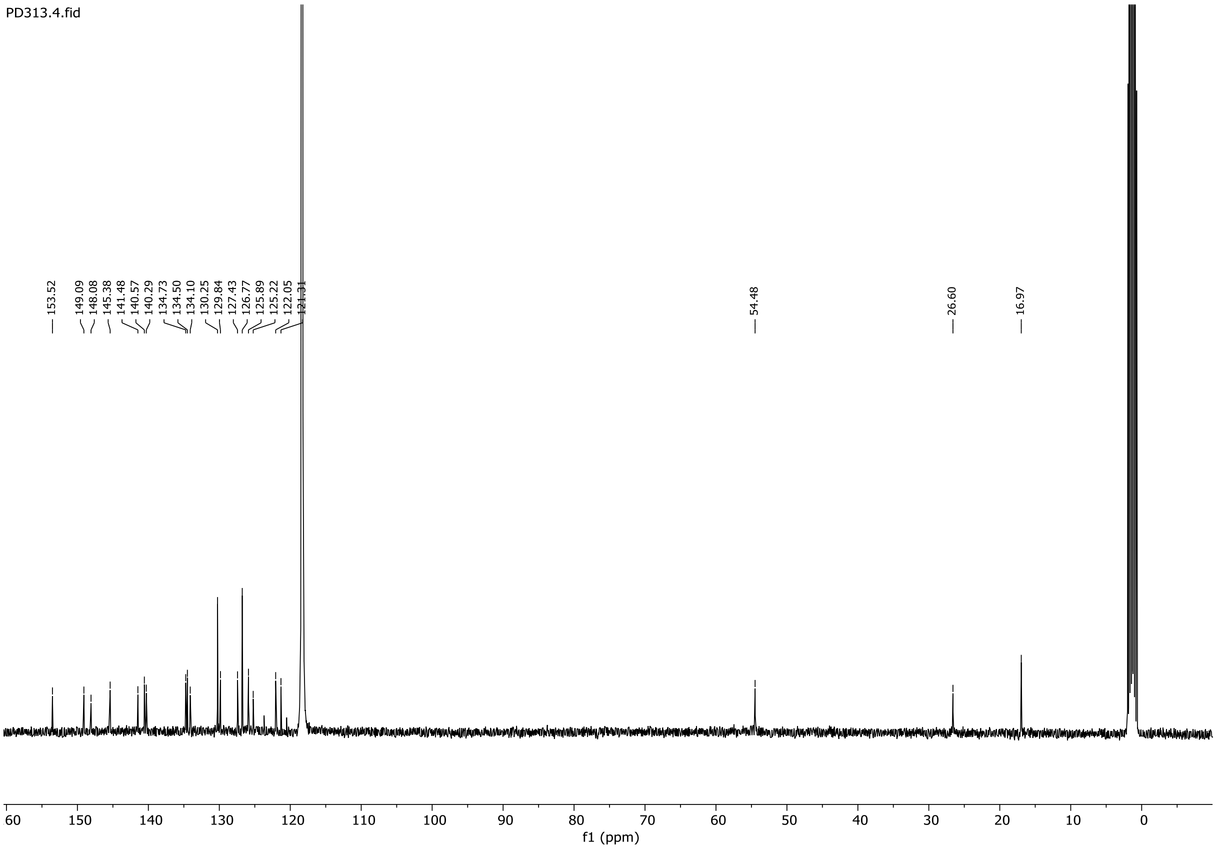

12 - HT276

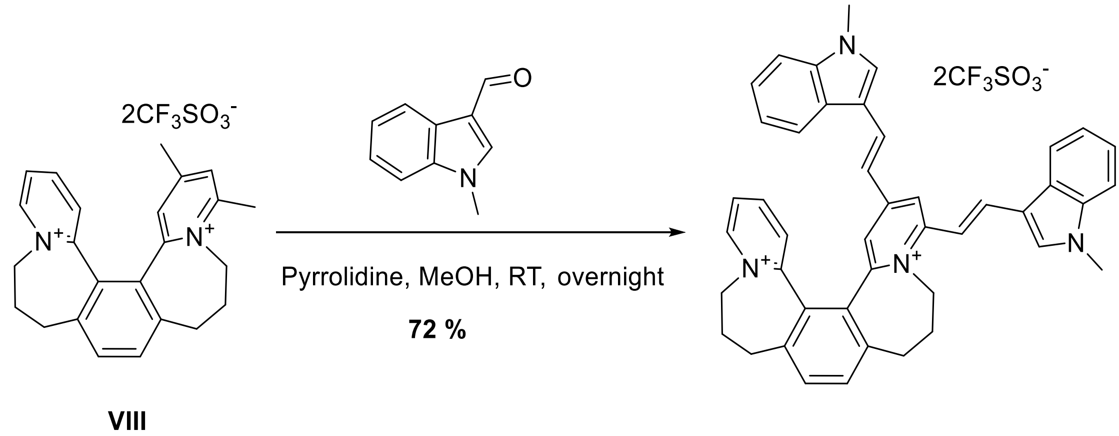

VIII

HT276

Helquat **VIII** (20 mg, 0.031 mmol), 1-methylindole-3-carboxaldehyde (79 mg, 0.5 mmol, 16.0 equiv.), pyrrolidine (0.021 mL, 0.25 mmol, 8.0 equiv.) and MeOH (1.0 mL) were placed in a 10-mL flask and stirred under argon at room temperature overnight while being protected from ambient light using an aluminium-foil cover. The reaction progress was checked by TLC (mobile-phase Stoddart's magic mixture). The crude product was transferred to 50-mL centrifuge tubes and precipitated from the reaction mixture by the addition of Et_2O (20 mL to each tube). The resulting suspensions were centrifuged and supernatants were removed. Residues were dissolved in a minimum amount of MeOH (0.5 mL), after which Et_2O (20 mL) was added to each tube. Precipitates were centrifuged. This reprecipitation was repeated twice. Centrifuged solids were collected in a glass vial using Et_2O. The resulting suspension was centrifuged and Et_2O was removed. The solids were dried under vacuum to obtain pure **HT276** as a red solid in 72% yield (21 mg, 0.023 mmol).

^{1}H NMR (401 MHz, CD_3CN) δ 8.96 (dd, *J* = 6.2, 1.4 Hz, 1H), 8.41 (d, *J* = 2.1 Hz, 1H), 8.28 (td, *J* = 7.9, 1.4 Hz, 1H), 8.05–7.90 (m, 2H), 7.79 (d, *J* = 16.1 Hz, 1H), 7.74–7.65 (m, 3H), 7.61 (dt, *J* = 8.1, 1.0 Hz, 1H), 7.55–7.46 (m, 3H), 7.43 (dd, *J* = 8.4, 1.0 Hz, 1H), 7.39–7.23 (m, 4H), 7.20–7.04 (m, 5H), 6.89 (d, *J* = 2.0 Hz, 1H), 4.93 (ddd, *J* = 25.8, 14.2, 6.0 Hz, 2H), 4.67 (td, *J* = 13.2, 5.4 Hz, 1H), 4.36–4.16 (m, 1H), 3.99 (s, 3H), 3.88 (s, 3H), 3.11–2.93 (m, 2H), 2.72 (dt, *J* = 12.2, 6.4 Hz, 2H), 2.60–2.26 (m, 5H).

^{13}C NMR (101 MHz, CD_3CN) δ 153.95, 153.29, 152.37, 152.30, 147.31, 146.27, 140.39, 140.34, 140.12, 139.88, 137.23, 136.83, 134.14, 133.93, 133.08, 132.71, 132.51, 130.76, 130.36, 128.60, 128.55, 126.35, 125.00, 124.97, 123.60, 122.31, 122.22, 121.59, 121.52, 121.44, 120.42, 105.10, 104.65, 59.37, 52.63, 32.44, 31.11, 30.79, 30.69, 29.38, 29.23.

MS (ESI+) m/z (%): 312 (100%, Z = 2)

HRMS (ESI+) m/z: $[(M\text{-}2TfO^-)^+]$ ($C_{44}H_{40}N_4$, Z = 2) calc.: 312.16210, found: 312.16200.

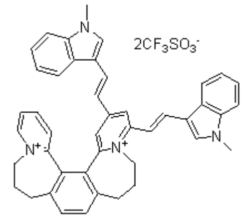

HT276

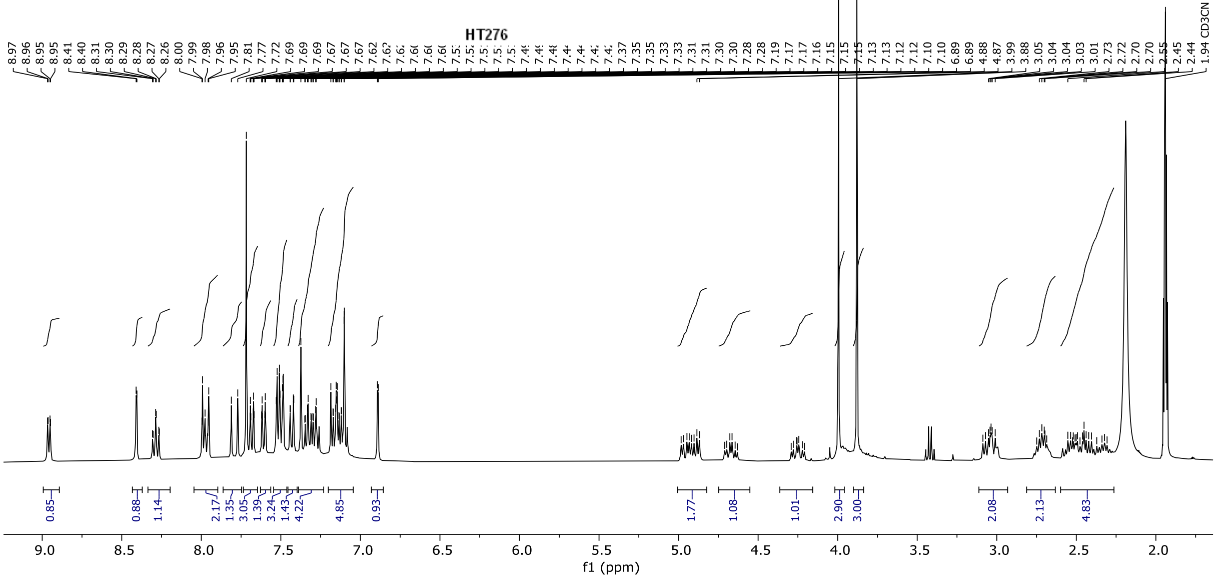

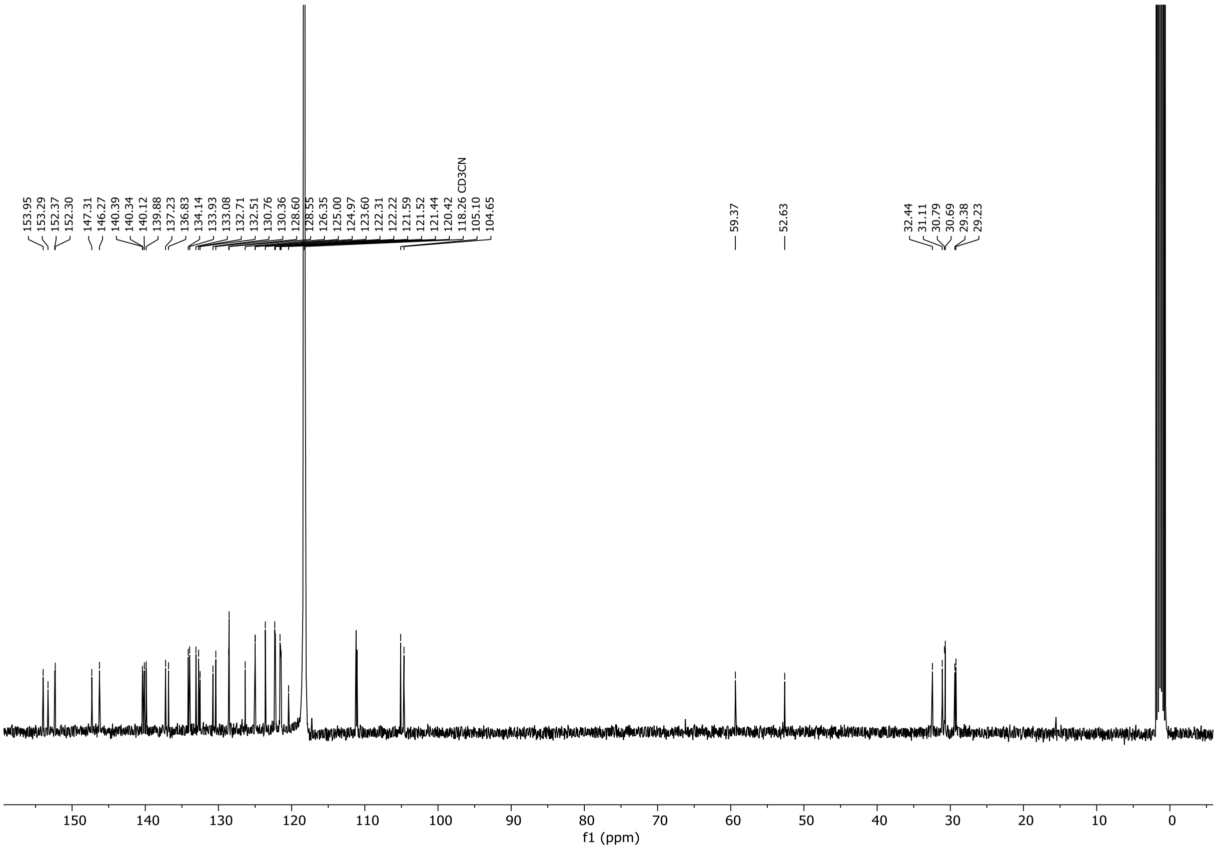

13 - PD283

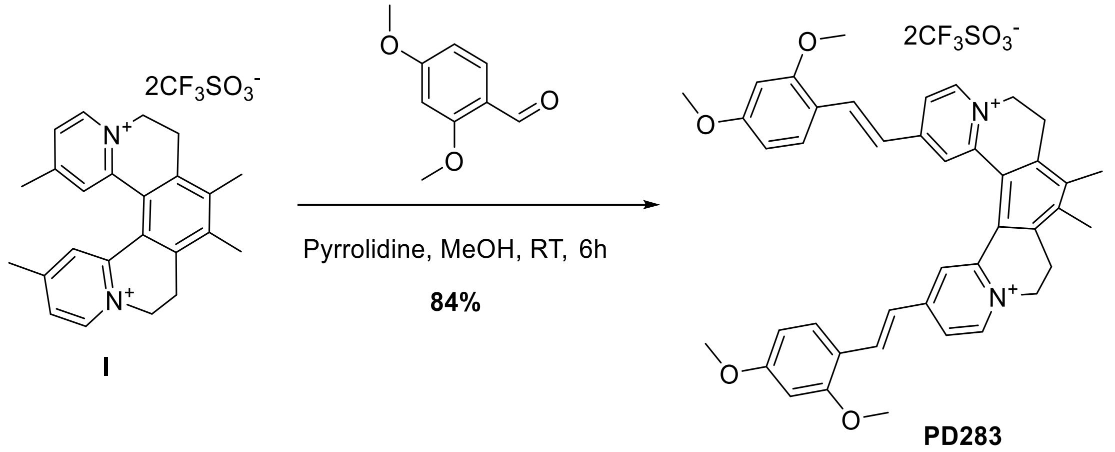

Helquat **I** (30 mg, 0.047 mmol), 2,4-dimethoxybenzaldehyde (233 mg, 1.4 mmol, 30.0 equiv.), pyrrolidine (0.07 mL, 0.70 mmol, 15.0 equiv.) and MeOH (2.0 mL) were placed in a 10-mL flask and stirred under argon at room temperature for six hours while being protected from ambient light using an aluminium-foil cover. The reaction progress was checked by TLC (mobile-phase Stoddart's magic mixture). The crude product was transferred to 50-mL centrifuge tubes and precipitated from the reaction mixture by the addition of Et_2O (30 mL to each tube). The resulting suspensions were centrifuged and supernatants were removed. Residues were dissolved in a minimum amount of MeOH (1.0 mL), after which Et_2O (20 mL) was added to each tube. Precipitates were centrifuged. This reprecipitation was repeated three more times. Centrifuged solids were collected in a glass vial using Et_2O. The resulting suspension was centrifuged and Et_2O was removed. The solids were dried under vacuum to obtain pure **PD283** as a yellow solid in 84% yield (37 mg, 0.039 mmol).

^{1}H NMR (401 MHz, acetonitrile-d_3) δ 8.61 (d, *J* = 6.6 Hz, 2H), 7.88–7.81 (m, 4H), 7.54 (d, *J* = 16.4 Hz, 2H), 7.49–7.41 (m, 2H), 7.05 (d, *J* = 16.3 Hz, 2H), 6.58–6.52 (m, 4H), 4.85 (ddd, *J* = 13.4, 5.0, 1.8 Hz, 2H), 4.70 (td, *J* = 13.9, 13.2, 3.7 Hz, 2H), 3.86 (d, *J* = 12.6 Hz, 12H), 3.47 (ddd, *J* = 17.2, 3.8, 1.8 Hz, 2H), 3.09 (ddd, *J* = 17.4, 14.6, 4.9 Hz, 2H), 2.45 (s, 6H).

^{13}C NMR (101 MHz, CD_3CN) δ 164.58, 161.02, 154.82, 147.87, 145.07, 141.32, 140.07, 137.37, 130.97, 126.93, 125.33, 121.68, 121.22, 117.58, 107.36, 99.25, 56.59, 56.39, 54.31, 26.56, 16.97.

MS (ESI+) m/z (%): 319 (100%, Z = 2).

HRMS (ESI+) m/z: [$(M\text{-}2TfO^-)^+$] ($C_{42}H_{42}N_2O_4$, Z = 2) calc.: 319.15668, found: 319.15656.

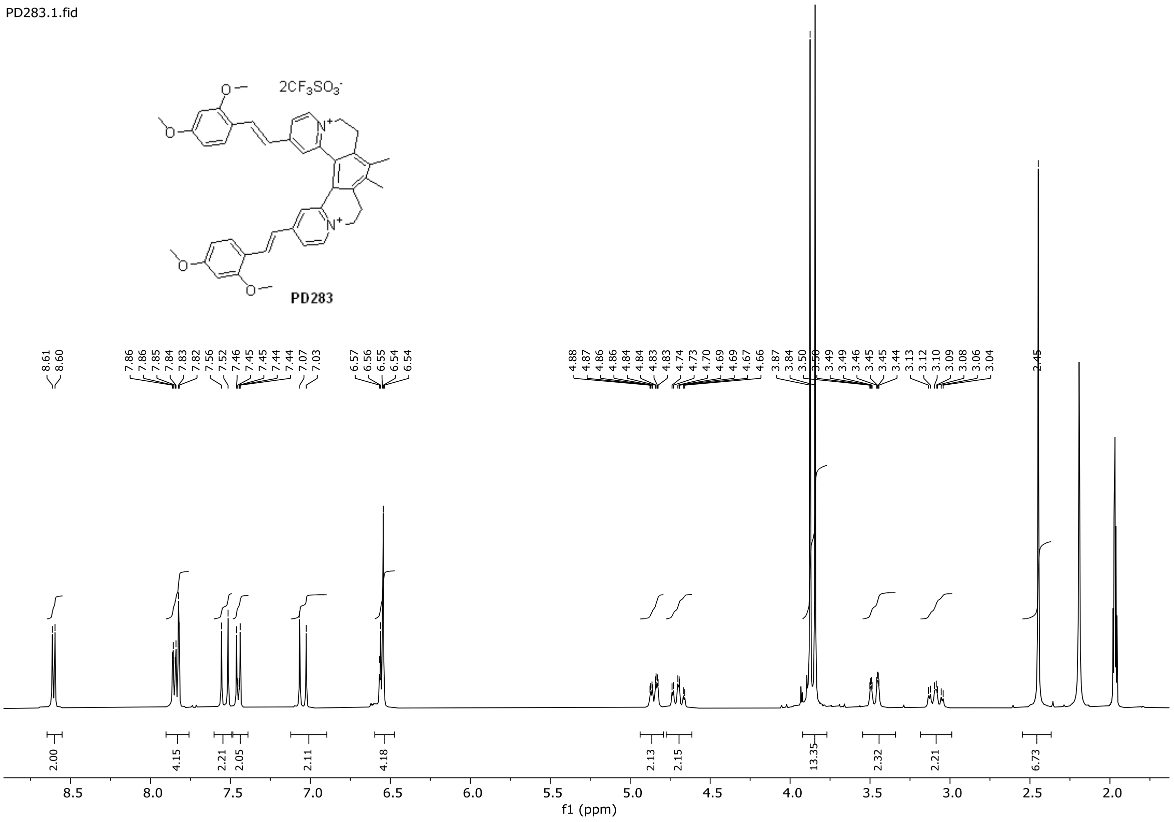
PD283.1.fid
2CF3SO3-
PD283
8.61 8.60
7.86 7.86 7.85 7.84 7.83 7.82 7.56 7.52 7.46 7.45 7.45 7.44 7.44 7.07 7.03
6.57 6.56 6.55 6.54 6.54
4.88 4.87 4.86 4.86 4.84 4.84 4.83 4.83 4.74 4.73 4.70 4.69 4.69 4.67 4.66
3.87 3.84 3.50 3.49 3.49 3.46 3.45 3.45 3.44
3.13 3.12 3.10 3.09 3.08 3.06 3.04
2.45
2.00 4.15 2.21 2.05 2.11 4.18 2.13 2.15 13.35 2.32 2.21 6.73
8.5 8.0 7.5 7.0 6.5 6.0 5.5 5.0 4.5 4.0 3.5 3.0 2.5 2.0
f1 (ppm)

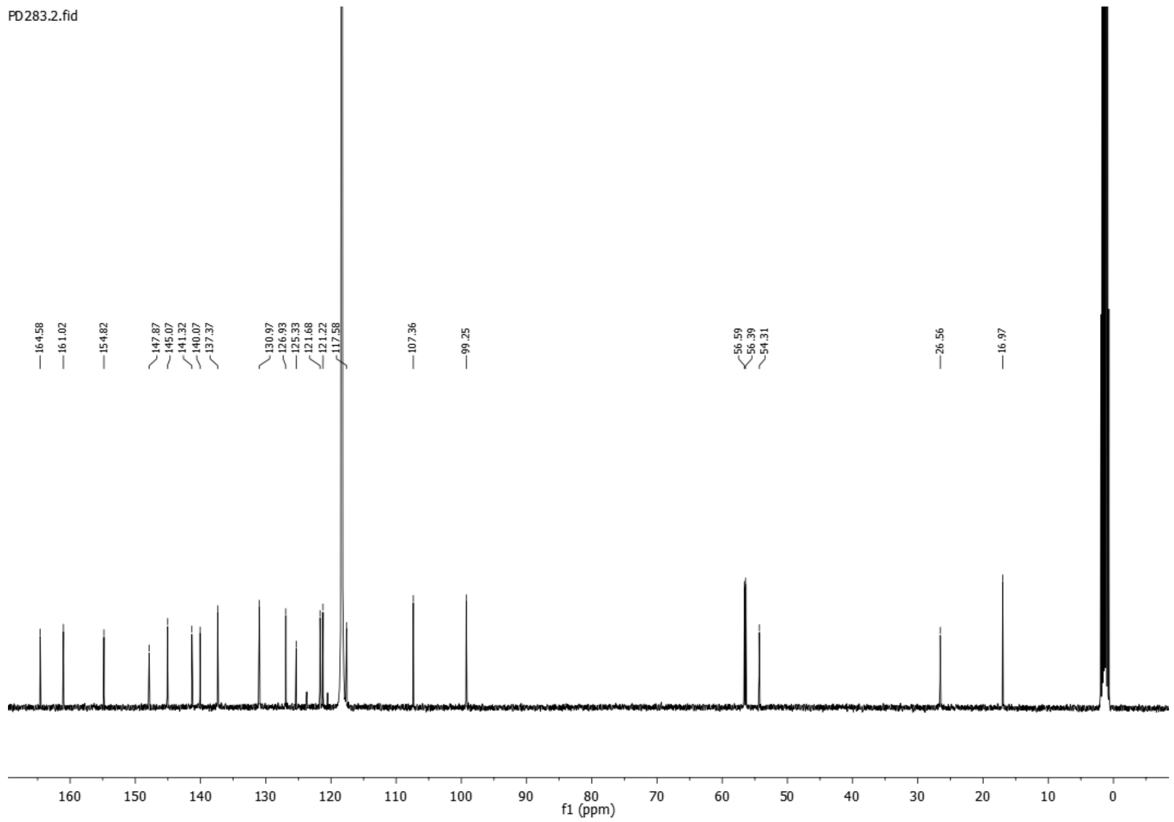
PD283.2.fid
164.58 161.02 154.82 147.87 145.07 141.32 140.07 137.37 130.97 126.93 125.33 121.68 121.22 117.58 107.36 99.25 56.59 56.39 54.31 26.56 16.97
160 150 140 130 120 110 100 90 80 70 60 50 40 30 20 10 0
f1 (ppm)

14 - HT311

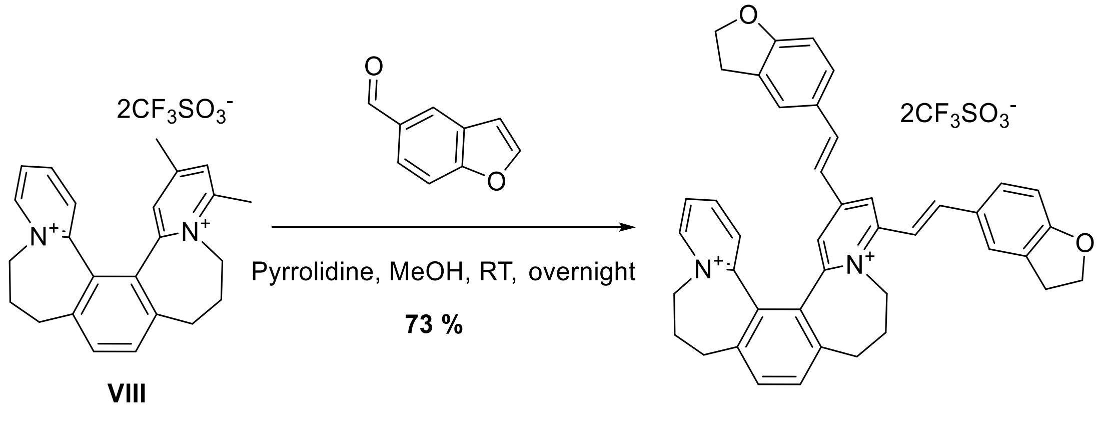

Helquat **VIII** (20 mg, 0.031 mmol), 2,3-dihydrobenzofuran-5-carboxaldehyde (74 mg, 0.5 mmol, 16.0 equiv.), pyrrolidine (0.021 mL, 0.25 mmol, 8.0 equiv.) and MeOH (1.0 mL) were placed in a 10-mL flask and stirred under argon at room temperature overnight while being protected from ambient light using an aluminium-foil cover. The reaction progress was checked by TLC (mobile-phase Stoddart's magic mixture). The crude product was transferred to 50-mL centrifuge tubes and precipitated from the reaction mixture by the addition of Et_2O (20 mL to each tube). The resulting suspensions were centrifuged and supernatants were removed. Residues were dissolved in a minimum amount of MeOH (0.5 mL), after which Et_2O (20 mL) was added to each tube. Precipitates were centrifuged. This reprecipitation was repeated twice. Centrifuged solids were collected in a glass vial using Et_2O. The resulting suspension was centrifuged and Et_2O was removed. The solids were dried under vacuum to obtain pure **HT311** as a yellow solid in 73% yield (20 mg, 0.022 mmol).

^{1}H NMR (401 MHz, acetonitrile-*d*$_3$) δ 8.95 (dd, *J* = 6.2, 1.4 Hz, 1H), 8.37 (d, *J* = 2.0 Hz, 1H), 8.26 (td, *J* = 7.9, 1.4 Hz, 1H), 7.97 (ddd, *J* = 7.8, 6.1, 1.5 Hz, 1H), 7.85 (d, *J* = 15.5 Hz, 1H), 7.79–7.67 (m, 4H), 7.62 (dd, *J* = 8.3, 1.0 Hz, 1H), 7.57–7.41 (m, 4H), 7.39–7.27 (m, 2H), 7.23–7.14 (m, 2H), 6.97 (d, *J* = 1.9 Hz, 1H), 4.92 (ddd, *J* = 24.1, 14.1, 6.0 Hz, 2H), 4.65 (td, *J* = 13.2, 5.5 Hz, 1H), 4.28 (td, *J* = 13.9, 5.2 Hz, 1H), 3.04 (td, *J* = 14.7, 6.6 Hz, 2H), 2.70 (ddt, *J* = 17.3, 13.3, 6.9 Hz, 2H), 2.59–2.32 (m, 4H).

^{13}C NMR (101 MHz, CD_3CN) δ 156.73, 156.68, 154.02, 153.81, 153.66, 153.11, 153.05, 152.26, 147.51, 146.41, 140.23, 140.05, 134.26, 134.22, 132.79, 132.29, 131.65, 130.87, 129.58, 129.56, 129.02, 128.61, 128.33, 128.18, 127.33, 124.87, 124.80, 123.82, 123.66, 123.44, 123.30, 122.27, 120.48, 118.80, 113.54, 113.47, 112.30, 112.16, 59.58, 53.12, 32.52, 31.23, 29.36, 29.32.

MS (ESI+) m/z (%): 301 (100%, Z = 2)

HRMS (ESI+) m/z: $[(M\text{-}2TfO^-)^+]$ ($C_{42}H_{39}N_2O_2$, Z = 2) calc.: 301.14612, found: 301.14636.

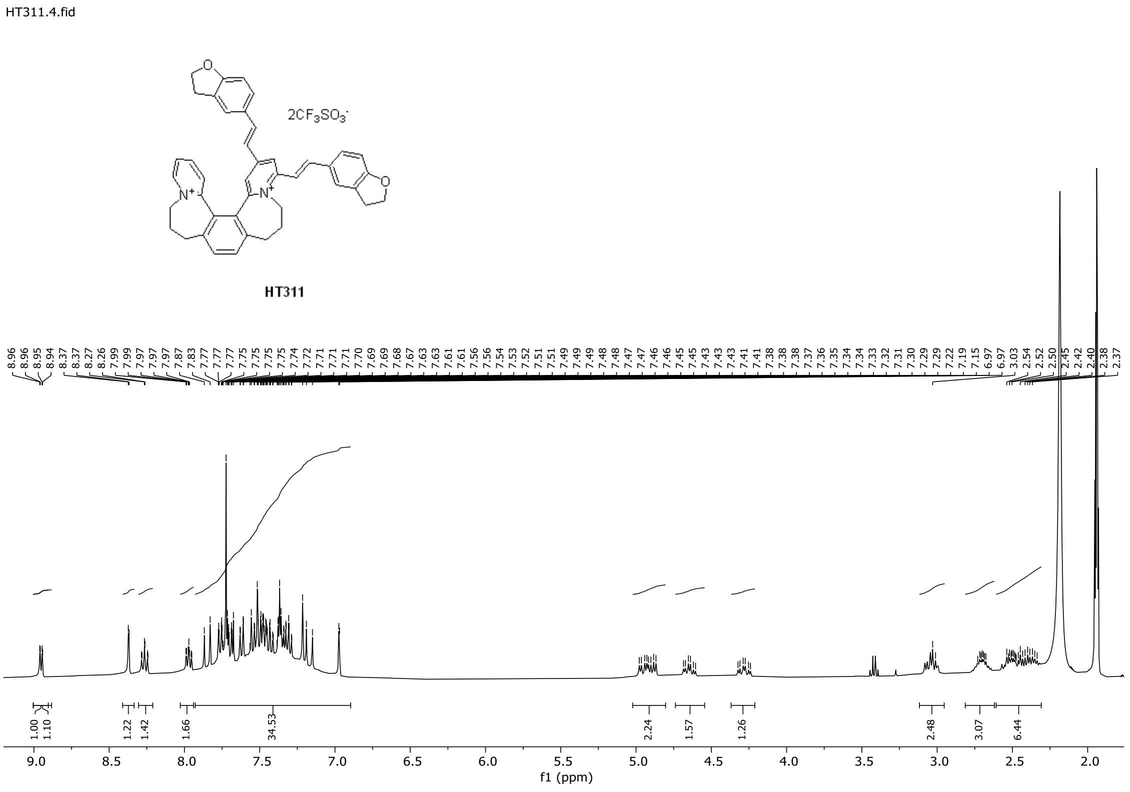
HT311.4.fid
$2CF_3SO_3^-$
HT311
8.96 8.96 8.95 8.94 8.37 8.37 8.27 8.26 7.99 7.99 7.97 7.97 7.97 7.87 7.83 7.77 7.77 7.77 7.75 7.75 7.75 7.75 7.74 7.72 7.71 7.71 7.71 7.70 7.69 7.68 7.67 7.63 7.63 7.61 7.61 7.56 7.56 7.54 7.53 7.52 7.51 7.51 7.49 7.49 7.49 7.48 7.48 7.47 7.47 7.46 7.46 7.45 7.45 7.43 7.43 7.43 7.41 7.41 7.38 7.38 7.38 7.37 7.36 7.35 7.34 7.34 7.33 7.32 7.31 7.30 7.29 7.29 7.22 7.19 7.15 6.97 6.97 3.03 2.54 2.52 2.50 2.45 2.42 2.40 2.38 2.37
1.00 1.10 1.22 1.42 1.66 34.53 2.24 1.57 1.26 2.48 3.07 6.44
9.0 8.5 8.0 7.5 7.0 6.5 6.0 5.5 5.0 4.5 4.0 3.5 3.0 2.5 2.0
f1 (ppm)

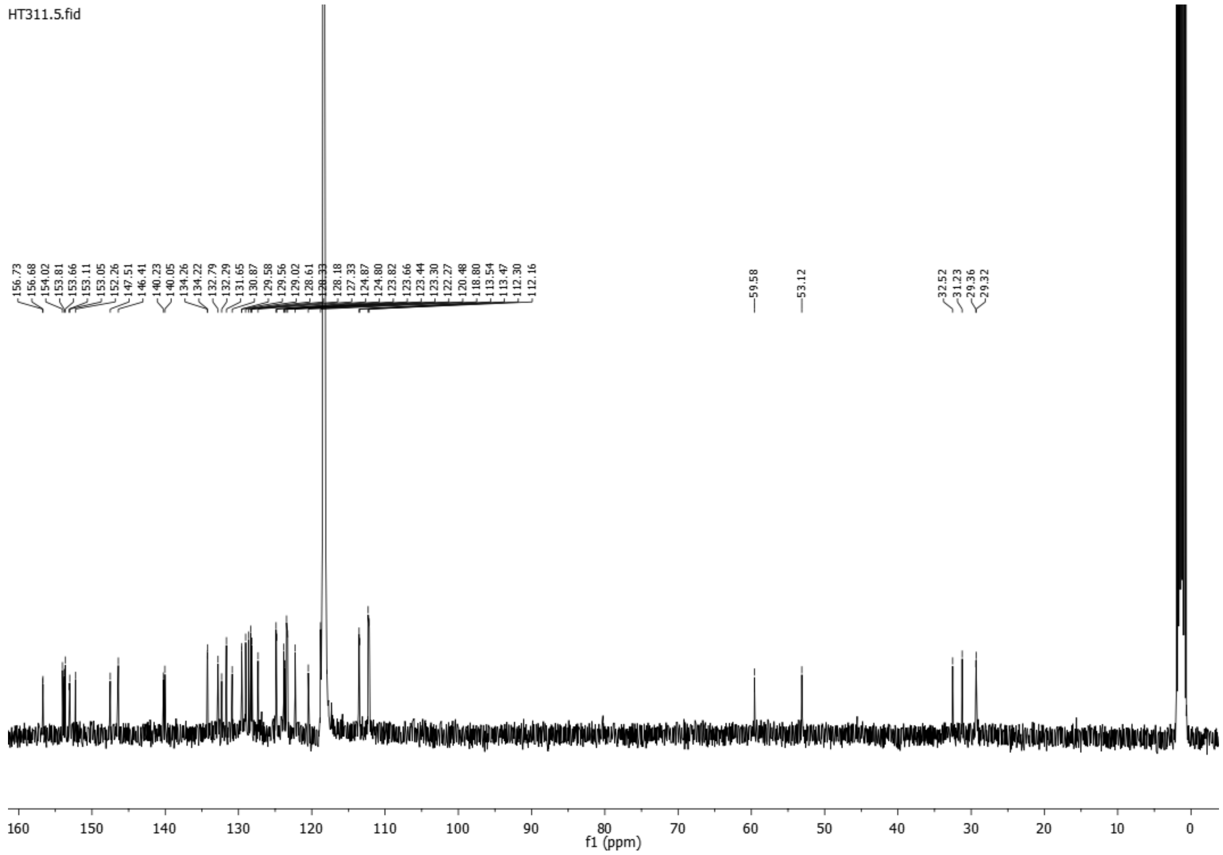
HT311.5.fid
156.73 156.68 154.02 153.81 153.66 153.11 153.05 152.26 147.51 146.41 140.23 140.05 134.26 134.22 132.79 132.29 131.65 130.87 129.58 129.56 129.02 128.61 128.33 128.18 127.33 124.87 124.80 123.82 123.66 123.44 123.30 122.27 120.48 118.80 113.54 113.47 112.30 112.16
59.58
53.12
32.52 31.23 29.36 29.32
160 150 140 130 120 110 100 90 80 70 60 50 40 30 20 10 0
f1 (ppm)

15 - PD290

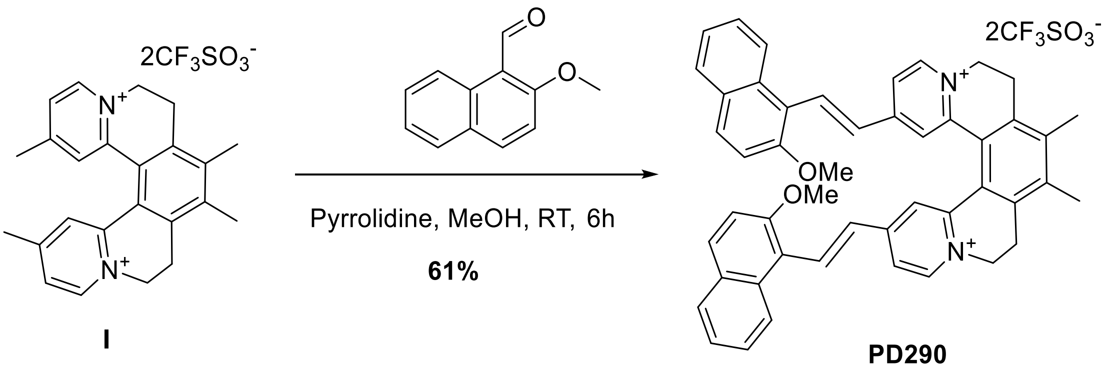

Helquat **I** (30 mg, 0.047 mmol), 2-methoxy-1-naphthaldehyde (262 mg, 1.4 mmol, 30.0 equiv.), pyrrolidine (0.07 mL, 0.70 mmol, 15.0 equiv.) and MeOH (2.0 mL) were placed in a 10-mL flask and stirred under argon at room temperature for six hours while being protected from ambient light using an aluminium-foil cover. The reaction progress was checked by TLC (mobile-phase Stoddart's magic mixture). The crude product was transferred to 50-mL centrifuge tubes and precipitated from the reaction mixture by the addition of Et_2O (30 mL to each tube). The resulting suspensions were centrifuged and supernatants were removed. Residues were dissolved in a minimum amount of MeOH (1.0 mL), after which Et_2O (20 mL) was added to each tube. Precipitates were centrifuged. This reprecipitation was repeated three more times. Centrifuged solids were collected in a glass vial using Et_2O. The resulting suspension was centrifuged and Et_2O was removed. The solids were dried under vacuum to obtain pure **PD290** as a red solid in 61% yield (28 mg, 0.029 mmol).

^{1}H NMR (401 MHz, acetonitrile-d_3) δ 8.70 (d, J = 6.6 Hz, 2H), 8.06 (dd, J = 6.6, 2.0 Hz, 2H), 8.00 (d, J = 2.0 Hz, 2H), 7.98–7.90 (m, 4H), 7.83 (ddd, J = 8.8, 5.4, 1.2 Hz, 4H), 7.52–7.33 (m, 8H), 4.93–4.84 (m, 2H), 4.76–4.64 (m, 2H), 3.90 (s, 6H), 3.49 (ddd, J = 17.4, 3.8, 1.8 Hz, 2H), 3.12 (td, J = 17.1, 16.1, 4.9 Hz, 2H), 2.46 (s, 6H).

^{13}C NMR (101 MHz, CD_3CN) δ 158.25, 154.79, 148.25, 145.58, 141.64, 140.34, 135.61, 133.51, 133.25, 129.88, 128.75, 128.55, 127.30, 125.31, 125.03, 123.65, 122.35, 118.07, 117.37, 114.32, 57.05, 54.59, 26.55, 17.08.

MS (ESI+) m/z (%): 339 (100%, Z = 2)

HRMS (ESI+) m/z: $[(M\text{-}2TfO^-)^+]$ ($C_{48}H_{42}N_2O_2$, Z = 2) calc.: 339.16177, found: 339.16191.

PD290.1.fid

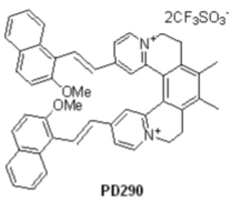

PD290

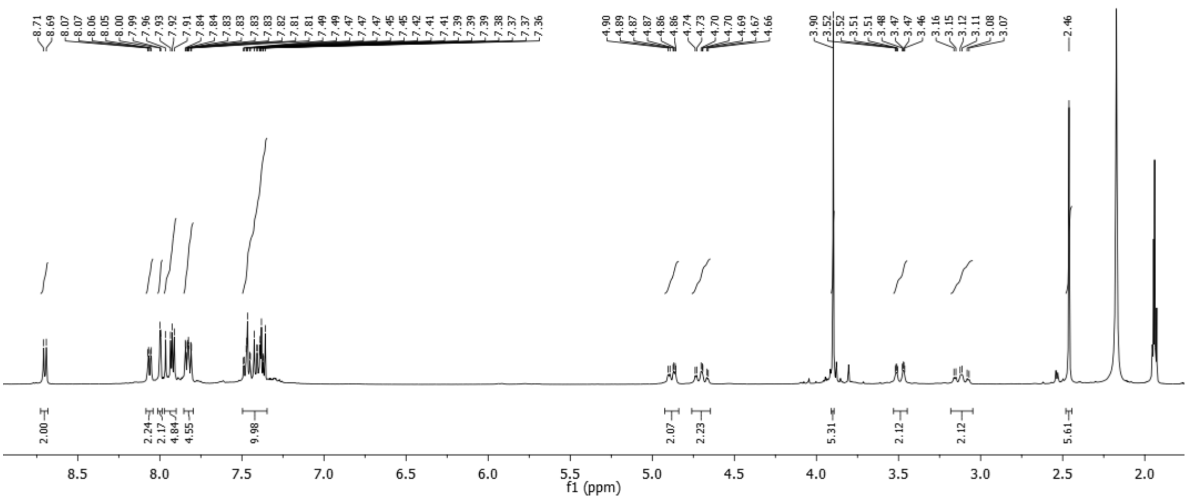

PD290.2.fid

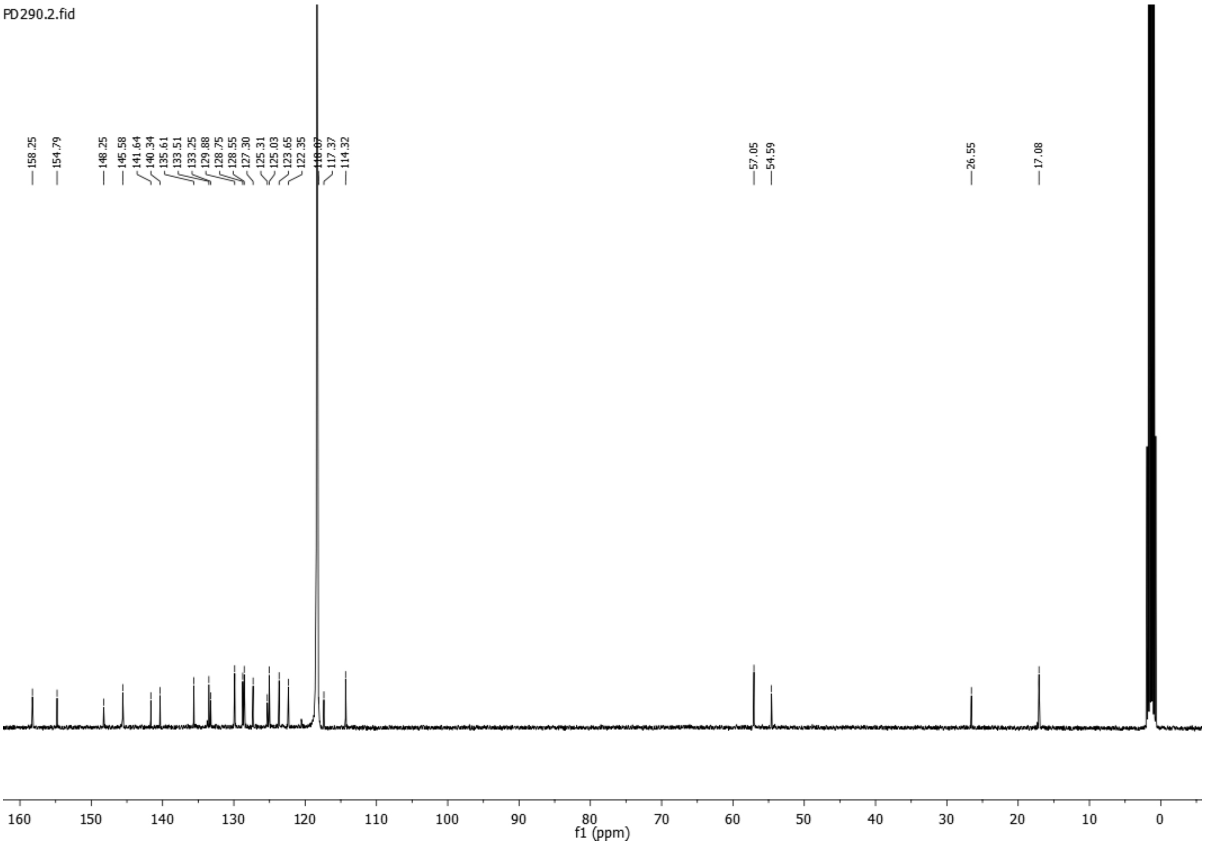

16 - VDJ845-10

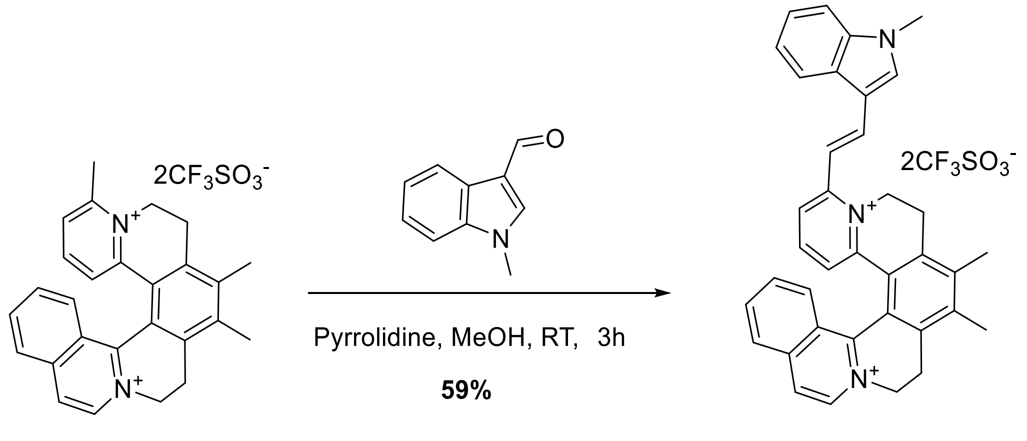

IX

VDJ845-10

Helquat **IX** (30 mg, 0.044 mmol), 1-methyl-1H-indole-3-carbaldehyde (70 mg, 0.44 mmol, 10.0 equiv.), pyrrolidine (0.02 mL, 0.22 mmol, 5.0 equiv.) and MeOH (3.0 mL) were placed in a 10-mL flask and stirred under argon at room temperature for three hours while being protected from ambient light using an aluminium-foil cover. The reaction progress was checked by TLC (mobile-phase Stoddart's magic mixture). The crude product was transferred to 50-mL centrifuge tubes and precipitated from the reaction mixture by the addition of Et_2O (16 mL to each tube). The resulting suspensions were centrifuged and supernatants were removed. Residues were dissolved in a minimum amount of MeOH (2.0 mL), after which Et_2O (20 mL) was added to each tube. Precipitates were centrifuged. This reprecipitation was repeated three more times. Centrifuged solids were collected in a glass vial using Et_2O. The resulting suspension was centrifuged and Et_2O was removed. The solids were dried under vacuum to obtain pure **VDJ845-10** as a red solid in 59% yield (21 mg, 0.026 mmol).

^{1}H NMR (401 MHz, acetonitrile-d_3) δ 8.61 (d, *J* = 6.7 Hz, 1H), 8.31 (d, *J* = 6.7 Hz, 1H), 8.12 (dd, *J* = 8.3, 1.1 Hz, 1H), 7.95–7.85 (m, 2H), 7.83–7.77 (m, 1H), 7.75–7.62 (m, 4H), 7.55–7.46 (m, 2H), 7.38–7.29 (m, 2H), 7.16 (ddd, *J* = 8.0, 7.0, 1.0 Hz, 1H), 6.87 (dd, *J* = 8.0, 1.3 Hz, 1H), 5.36 (ddd, *J* = 13.9, 4.9, 1.9 Hz, 1H), 5.02 (ddd, *J* = 13.9, 4.7, 1.9 Hz, 1H), 4.76 (dtd, *J* = 32.5, 14.3, 3.6 Hz, 2H), 3.92 (s, 3H), 3.57 (dddd, *J* = 19.3, 17.1, 3.7, 1.9 Hz, 2H), 3.25–2.99 (m, 2H), 2.54 (d, *J* = 5.7 Hz, 6H).

^{13}C NMR (101 MHz, CD_3CN) δ 154.43, 151.62, 148.05, 143.09, 142.29, 142.14, 140.99, 140.46, 139.82, 138.46, 136.93, 136.68, 136.30, 133.32, 132.70, 129.12, 128.63, 128.59, 128.51, 128.42, 126.86, 125.79, 125.26, 125.21, 123.63, 123.38, 122.41, 121.65, 120.44, 117.96, 111.21, 105.50, 55.98, 50.25, 30.73, 26.66, 25.86, 17.05, 16.95.

MS (ESI) m/z (%): 260 (100%, Z = 2)

VDJ845-10.1.fid

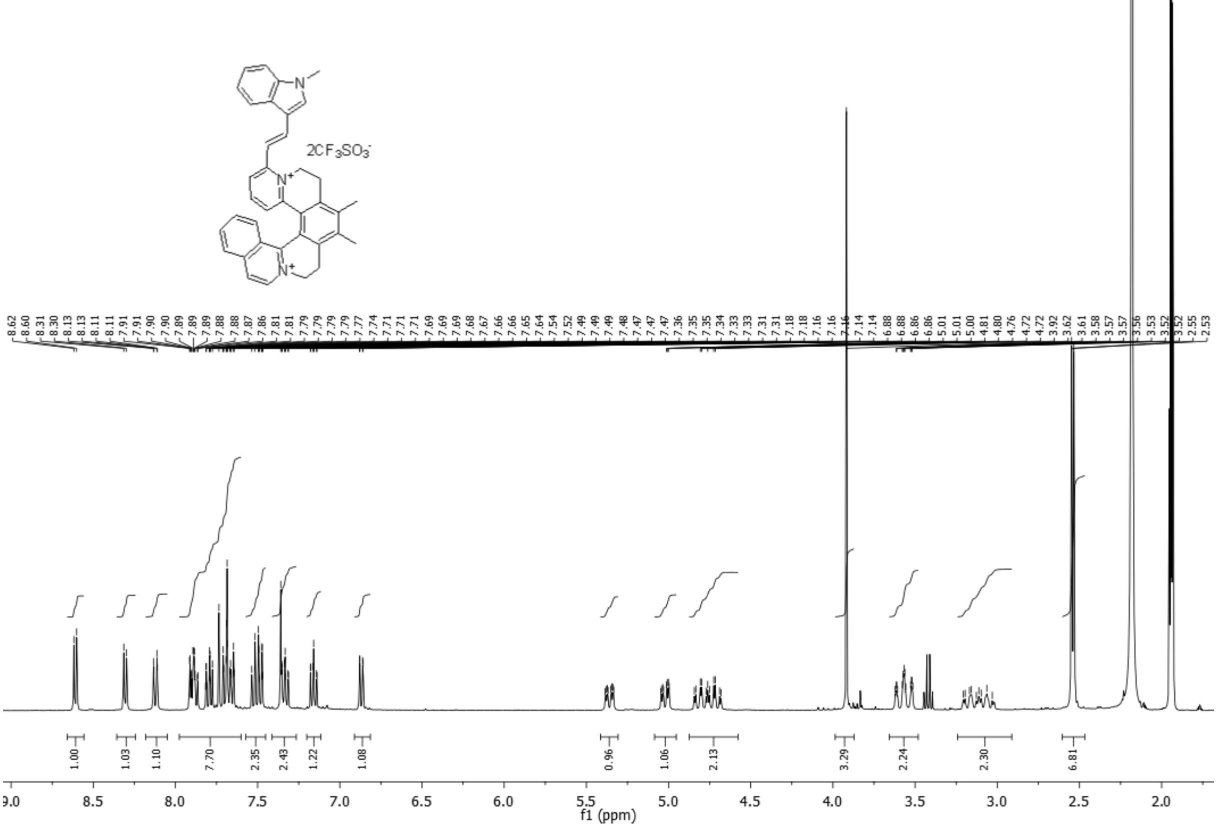

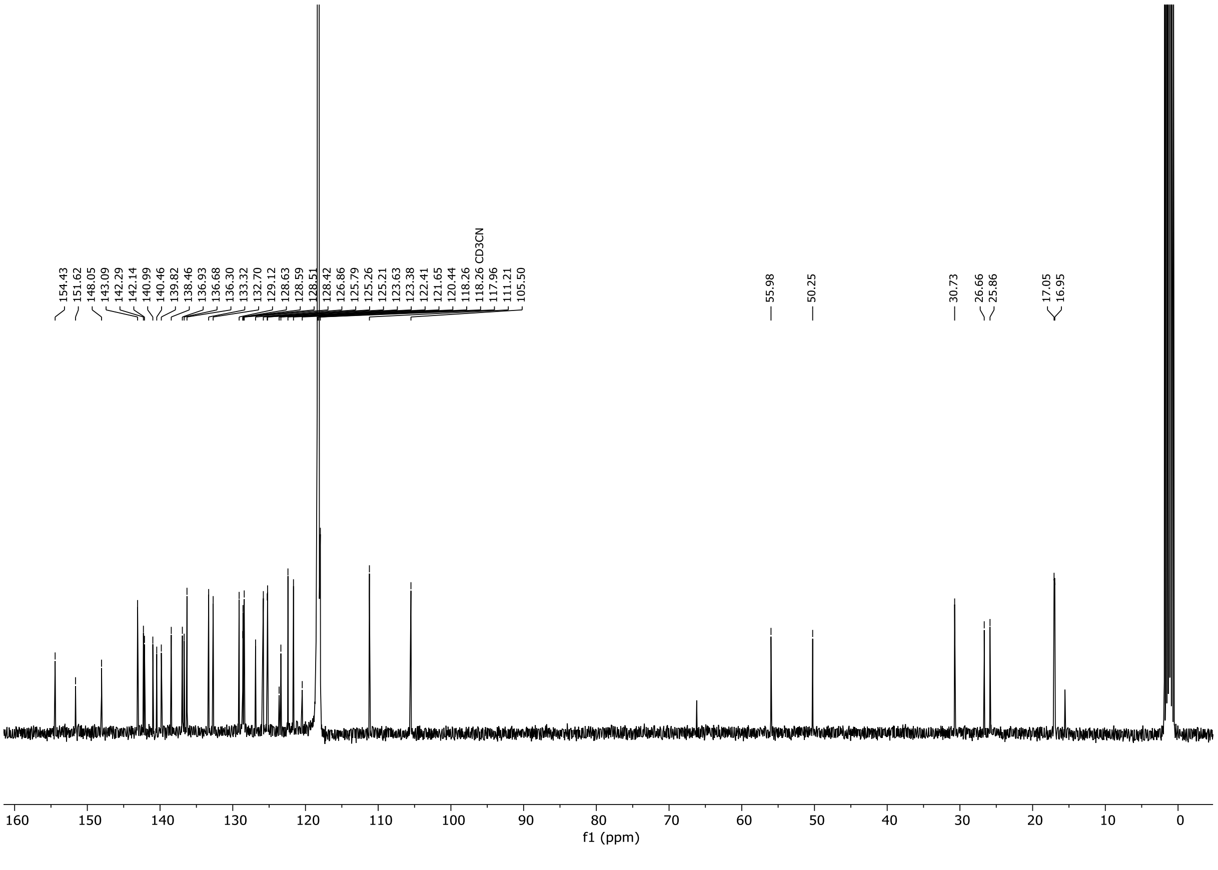

17 - PD263

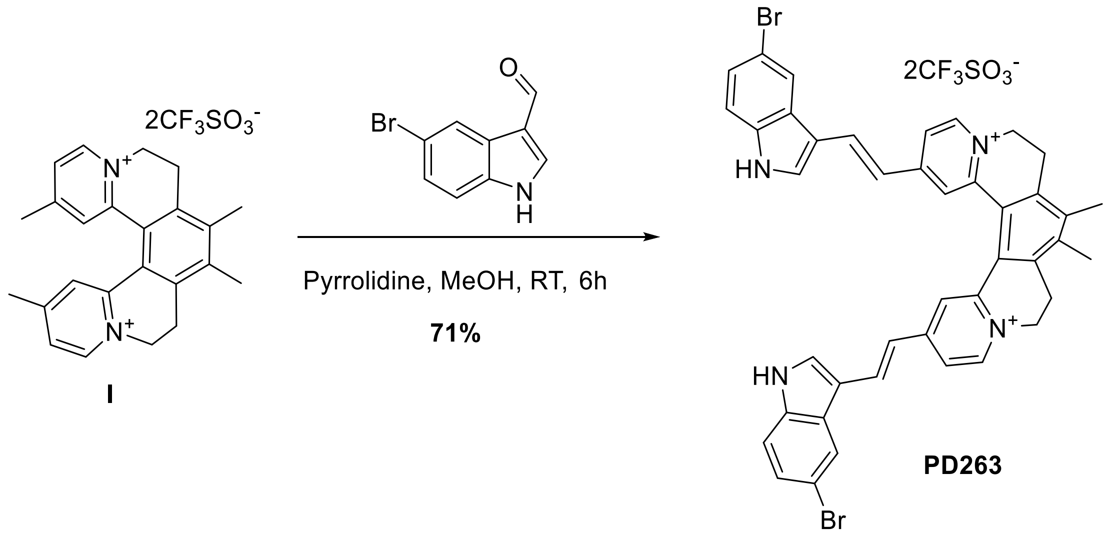

Helquat **I** (30 mg, 0.047 mmol), 5-bromo-1H-indole-3-carbaldehyde (315 mg, 1.4 mmol, 30.0 equiv.), pyrrolidine (0.07 mL, 0.70 mmol, 15.0 equiv.) and MeOH (2.0 mL) were placed in a 10-mL flask and stirred under argon at room temperature for six hours while being protected from ambient light using an aluminium-foil cover. The reaction progress was checked by TLC (mobile-phase Stoddart's magic mixture). The crude product was transferred to 50-mL centrifuge tubes and precipitated from the reaction mixture by the addition of Et_2O (30 mL to each tube). The resulting suspensions were centrifuged and supernatants were removed. Residues were dissolved in a minimum amount of MeOH (1.0 mL), after which Et_2O (20 mL) was added to each tube. Precipitates were centrifuged. This reprecipitation was repeated three more times. Centrifuged solids were collected in a glass vial using Et_2O. The resulting suspension was centrifuged and Et_2O was removed. The solids were dried under vacuum to obtain pure **PD263** as a red solid in 71% yield (35 mg, 0.03 mmol).

^{1}H NMR (401 MHz, DMSO-d_6) δ 8.99 (d, *J* = 6.7 Hz, 2H), 8.21 (d, *J* = 1.9 Hz, 2H), 8.08 (dd, *J* = 6.7, 2.0 Hz, 2H), 7.99 (d, *J* = 1.9 Hz, 2H), 7.81–7.72 (m, 4H), 7.41 (d, *J* = 8.5 Hz, 2H), 7.31 (dd, *J* = 8.6, 1.9 Hz, 2H), 6.95 (d, *J* = 16.2 Hz, 2H), 4.97 (dd, *J* = 13.5, 4.2 Hz, 2H), 4.75 (td, *J* = 13.8, 3.6 Hz, 2H), 3.51 (d, *J* = 16.7 Hz, 2H), 3.19–3.05 (m, 2H), 2.43 (s, 6H).

^{13}C NMR (101 MHz, DMSO) δ 153.03, 146.21, 143.94, 139.09, 138.56, 135.93, 134.63, 131.70, 126.75, 125.33, 124.72, 124.51, 121.89, 119.80, 117.59, 114.57, 113.78, 112.78, 52.53, 25.39, 16.38.

MS (ESI+) m/z (%): 376 (100%, Z = 2), 377 (48%, Z = 2)

HRMS (ESI+) m/z: [(M-2TfO$^-$)$^+$] ($C_{42}H_{34}N_4Br_2$, Z = 2) calc.: 373.18250, found: 373.18271.

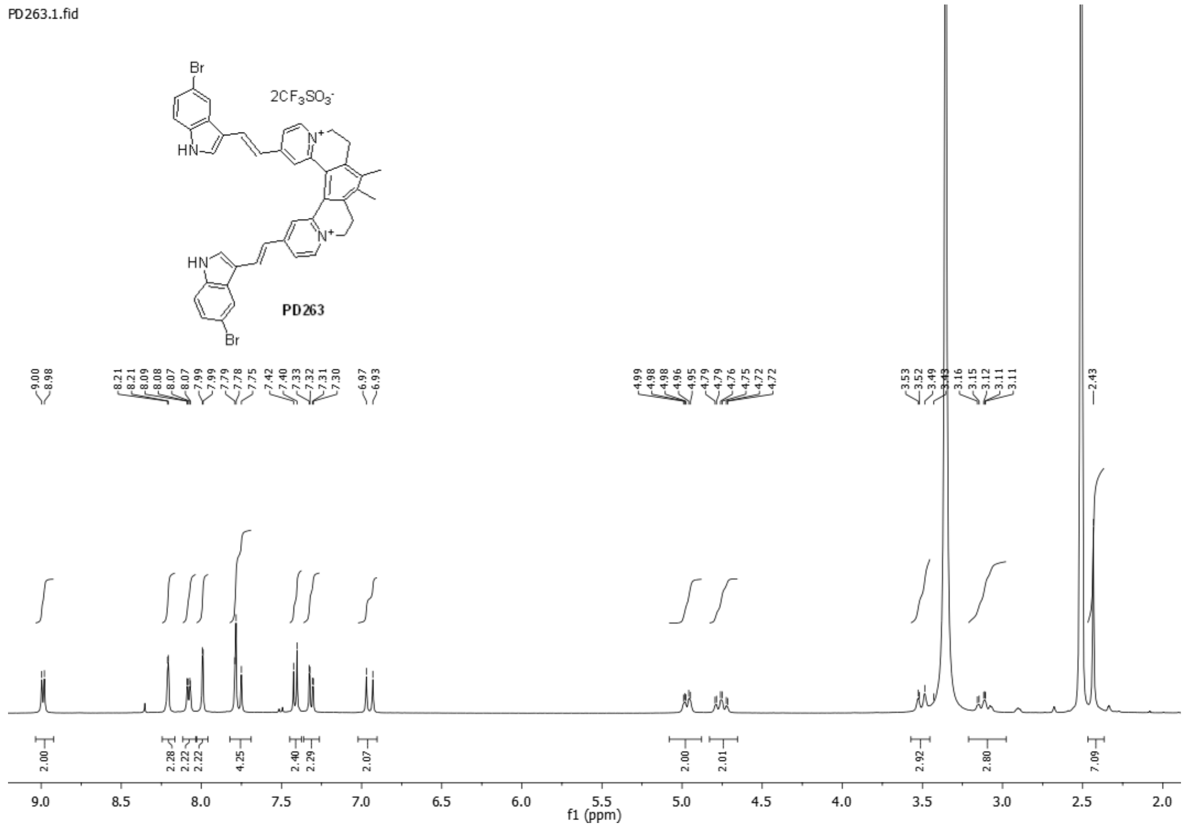
PD263.1.fid
2CF3SO3-
Br
HN
N+
PD263
9.00 8.98 8.21 8.21 8.09 8.08 8.07 8.07 7.99 7.99 7.79 7.78 7.75 7.42 7.40 7.33 7.32 7.31 7.30 6.97 6.93
4.99 4.98 4.98 4.96 4.95 4.79 4.79 4.76 4.75 4.72 4.72
3.53 3.52 3.49 3.43 3.16 3.15 3.12 3.11 3.11 2.43
2.00 2.28 2.22 2.22 4.25 2.40 2.29 2.07 2.00 2.01 2.92 2.80 7.09
9.0 8.5 8.0 7.5 7.0 6.5 6.0 5.5 5.0 4.5 4.0 3.5 3.0 2.5 2.0
f1 (ppm)

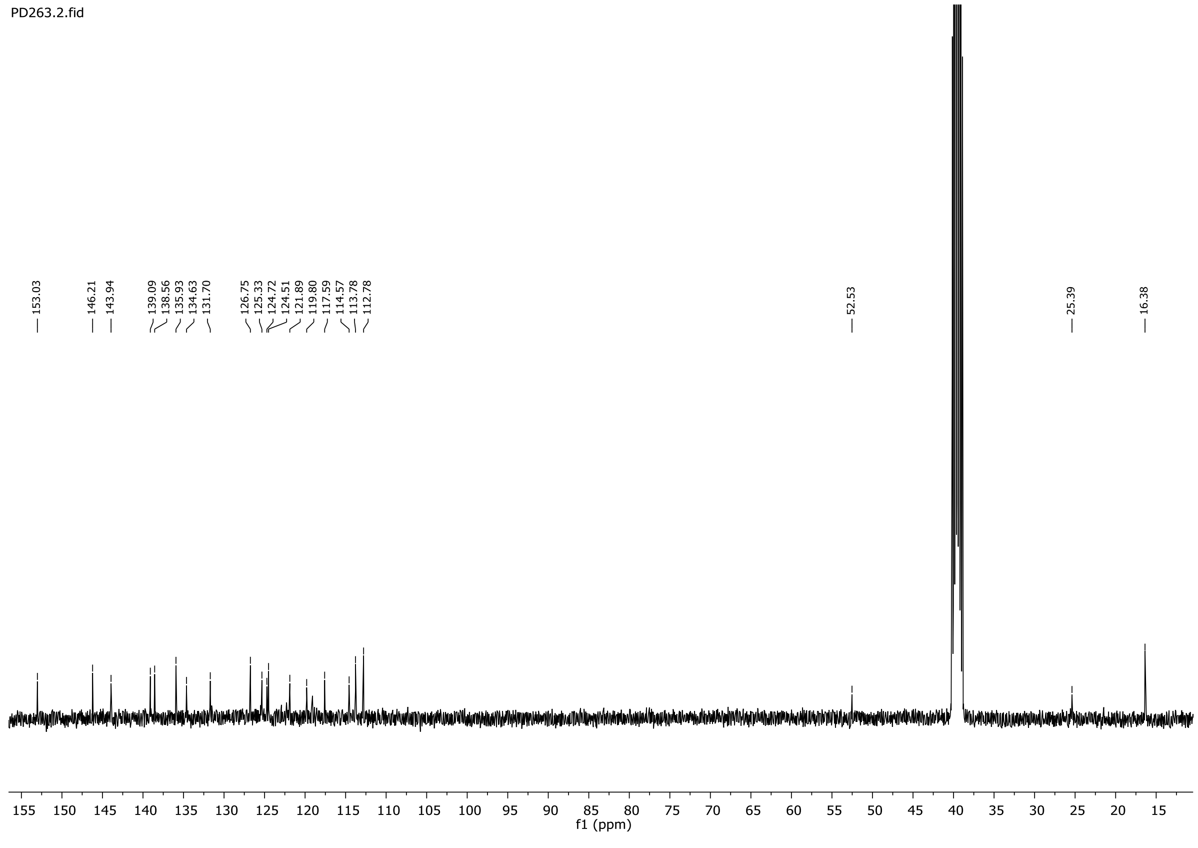
PD263.2.fid
153.03 146.21 143.94 139.09 138.56 135.93 134.63 131.70 126.75 125.33 124.72 124.51 121.89 119.80 117.59 114.57 113.78 112.78 52.53 25.39 16.38
155 150 145 140 135 130 125 120 115 110 105 100 95 90 85 80 75 70 65 60 55 50 45 40 35 30 25 20 15
f1 (ppm)

18 - PD262

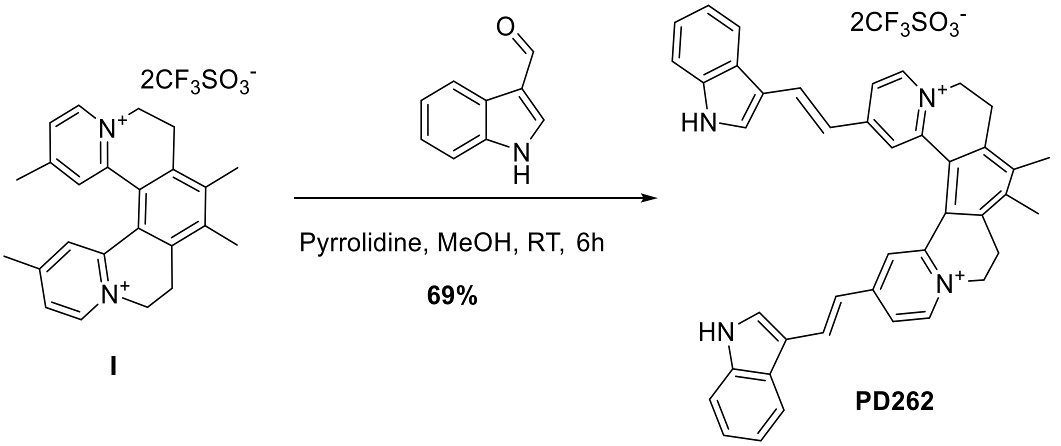

Helquat **I** (30 mg, 0.047 mmol), 1H-indole-3-carbaldehyde (204 mg, 1.4 mmol, 30.0 equiv.), pyrrolidine (0.07 mL, 0.70 mmol, 15.0 equiv.) and MeOH (2.0 mL) were placed in a 10-mL flask and stirred under argon at room temperature for six hours while being protected from ambient light using an aluminium-foil cover. The reaction progress was checked by TLC (mobile-phase Stoddart's magic mixture). The crude product was transferred to 50-mL centrifuge tubes and precipitated from the reaction mixture by the addition of Et_2O (30 mL to each tube). The resulting suspensions were centrifuged and supernatants were removed. Residues were dissolved in a minimum amount of MeOH (1.0 mL), after which Et_2O (20 mL) was added to each tube. Precipitates were centrifuged. This reprecipitation was repeated three more times. Centrifuged solids were collected in a glass vial using Et_2O. The resulting suspension was centrifuged and Et_2O was removed. The solids were dried under vacuum to obtain pure **PD262** as a red solid in 69% yield (29 mg, 0.032 mmol).

^{1}H NMR (401 MHz, acetonitrile-d_3) δ 10.71 (s, 2H), 8.54 (d, *J* = 6.8 Hz, 2H), 7.86–7.79 (m, 4H), 7.76 (d, *J* = 1.9 Hz, 2H), 7.67 (d, *J* = 16.1 Hz, 2H), 7.59 (s, 2H), 7.51–7.43 (m, 2H), 7.27–7.14 (m, 4H), 6.93 (d, *J* = 16.1 Hz, 2H), 4.81 (dd, *J* = 13.3, 4.5 Hz, 2H), 4.65 (td, *J* = 13.9, 3.6 Hz, 2H), 3.43 (d, *J* = 16.9 Hz, 2H), 3.14–2.98 (m, 2H), 2.41 (s, 6H).

^{13}C NMR (101 MHz, CD_3CN) δ 155.27, 147.56, 144.68, 140.94, 139.93, 138.69, 137.24, 133.03, 126.05, 125.75, 125.46, 124.22, 122.43, 120.93, 120.26, 117.69, 114.70, 113.60, 53.98, 26.69, 16.91.

MS (ESI+) m/z (%): 298 (100%, M-2TfO$^-$,Z = 2), 595 (40%, M-2TfO$^-$), 745 (10%, M-TfO$^-$)

HRMS (ESI+) m/z: [(M-2TfO$^-$)$^+$] ($C_{42}H_{36}N_4$, Z = 2) calc.: 298.14645, found: 298.14685.

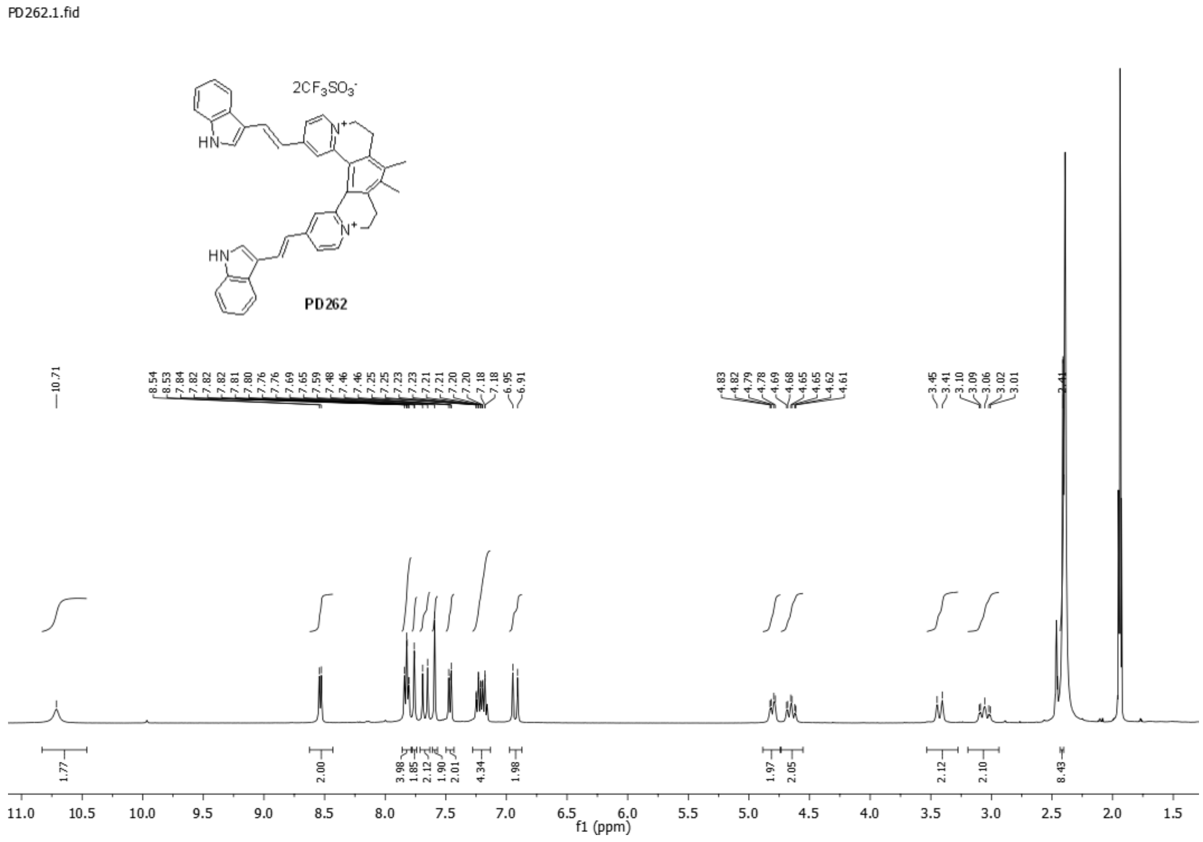
PD262.1.fid
2CF3SO3-
HN
N+
PD262
10.71
8.54 8.53 7.84 7.82 7.82 7.82 7.81 7.80 7.76 7.76 7.69 7.65 7.59 7.48 7.46 7.46 7.25 7.25 7.23 7.23 7.21 7.21 7.20 7.20 7.18 7.18 6.95 6.91
4.83 4.82 4.79 4.78 4.69 4.68 4.65 4.65 4.62 4.61
3.45 3.41 3.10 3.09 3.06 3.02 3.01
2.41
1.77
2.00
3.98 1.85 2.12 1.90 2.01
4.34
1.98
1.97 2.05
2.12 2.10
8.43
f1 (ppm)

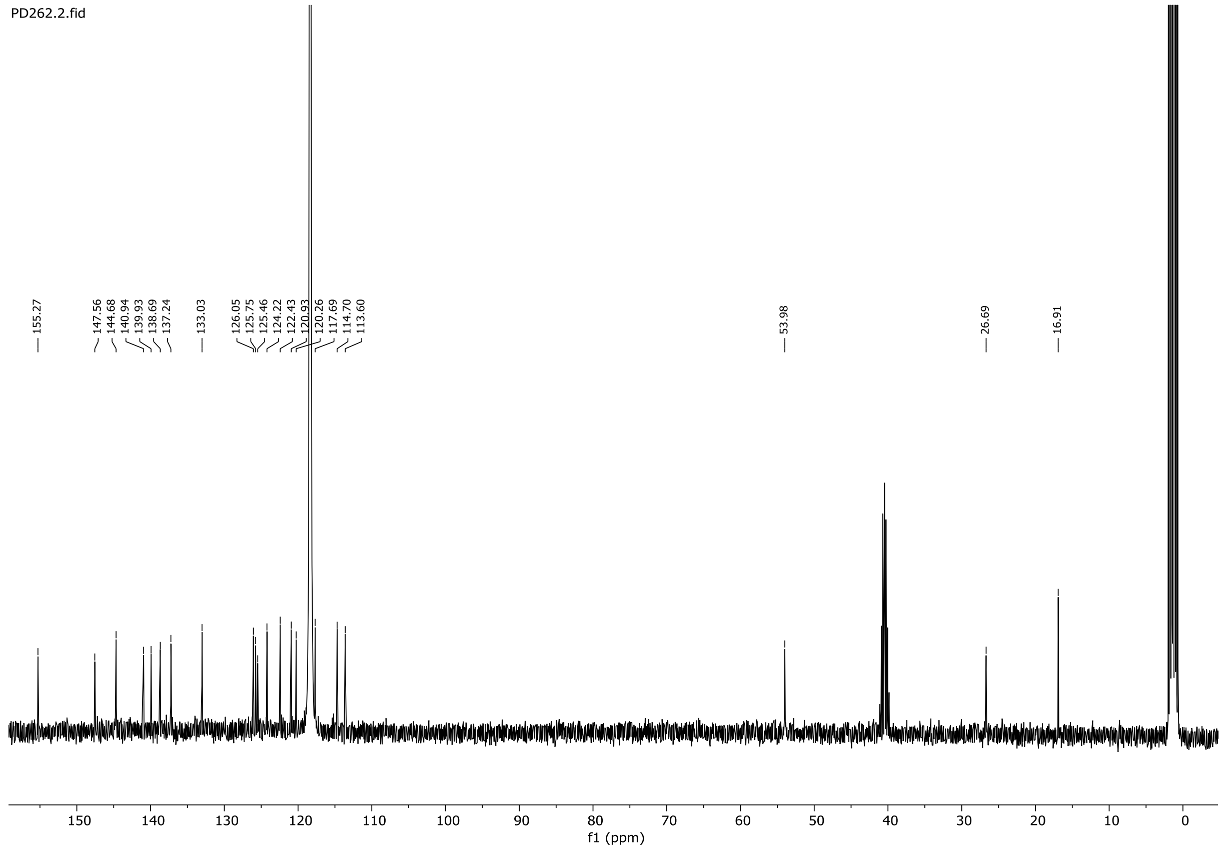
PD262.2.fid
155.27
147.56 144.68 140.94 139.93 138.69 137.24
133.03
126.05 125.75 125.46 124.22 122.43 120.93 120.26 117.69 114.70 113.60
53.98
26.69
16.91
f1 (ppm)

19 - PD209

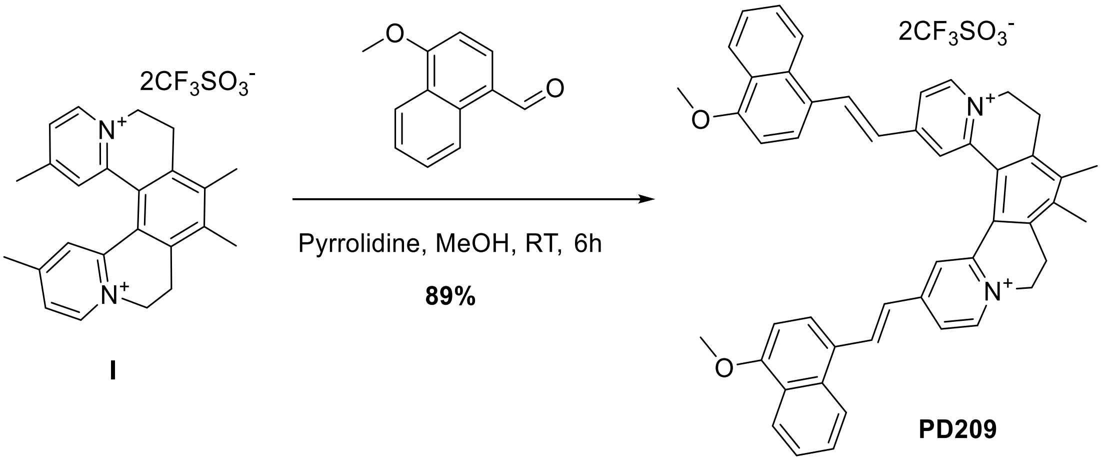

Helquat **I** (30 mg, 0.047 mmol), 4-methoxy-1-naphthaldehyde (260 mg, 1.4 mmol, 30.0 equiv.), pyrrolidine (0.07 mL, 0.70 mmol, 15.0 equiv.) and MeOH (2.0 mL) were placed in a 10-mL flask and stirred under argon at room temperature for six hours while being protected from ambient light using an aluminium-foil cover. The reaction progress was checked by TLC (mobile-phase Stoddart's magic mixture). The crude product was transferred to 50-mL centrifuge tubes and precipitated from the reaction mixture by the addition of Et_2O (30 mL to each tube). The resulting suspensions were centrifuged and supernatants were removed. Residues were dissolved in a minimum amount of MeOH (1.0 mL), after which Et_2O (20 mL) was added to each tube. Precipitates were centrifuged. This reprecipitation was repeated three more times. Centrifuged solids were collected in a glass vial using Et_2O. The resulting suspension was centrifuged and Et_2O was removed. The solids were dried under vacuum to obtain pure **PD209** as a red solid in 89% yield (41 mg, 0.042 mmol).

^{1}H NMR (401 MHz, acetonitrile-d_3) δ 8.88 (d, J = 6.6 Hz, 2H), 8.28–8.19 (m, 4H), 8.12–8.04 (m, 4H), 7.83 (d, J = 8.4 Hz, 2H), 7.76 (d, J = 8.3 Hz, 2H), 7.62 (ddd, J = 8.5, 6.8, 1.5 Hz, 2H), 7.54 (ddd, J = 8.0, 6.8, 1.2 Hz, 2H), 7.11 (d, J = 15.9 Hz, 2H), 6.91 (d, J = 8.3 Hz, 2H), 4.93 (dd, J = 13.5, 4.5 Hz, 2H), 4.74 (td, J = 14.1, 13.5, 3.7 Hz, 2H), 3.97 (s, 6H), 3.51–3.41 (m, 2H), 3.10 (td, J = 15.6, 4.4 Hz, 2H), 2.43 (s, 6H).

^{13}C NMR (101 MHz, CD_3CN) δ 158.49, 153.64, 148.02, 145.58, 141.13, 140.04, 138.51, 132.96, 128.48, 127.24, 126.85, 126.72, 126.05, 125.41, 125.11, 123.75, 123.71, 123.36, 122.63, 105.48, 56.65, 54.31, 26.45, 17.05.

MS (ESI+) m/z (%): 339 (100%, Z = 2).

HRMS (ESI+) m/z: [(M-2TfO$^-$)$^+$] ($C_{48}H_{42}N_2O_2$, Z = 2) calc.: 339.16177, found: 339.16150.

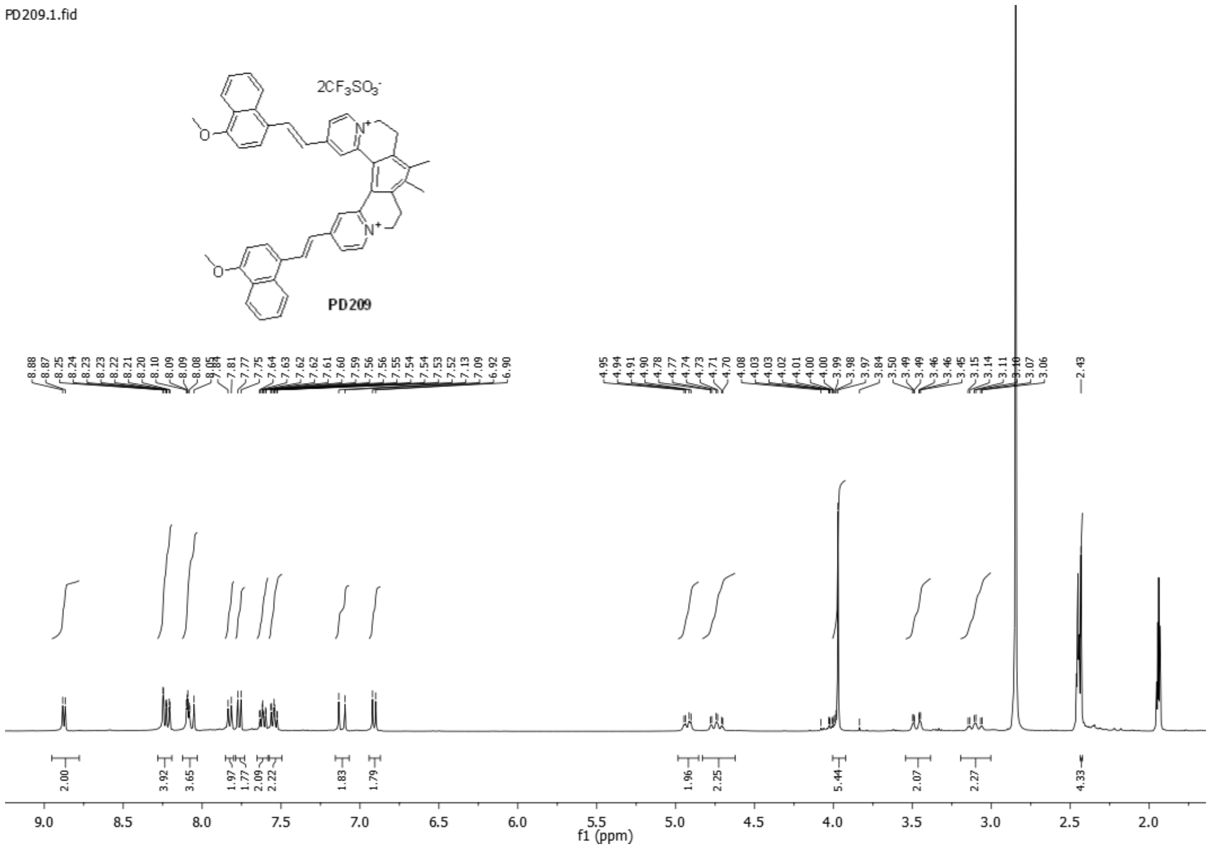
PD209.1.fid
2CF3SO3-
PD209
f1 (ppm)

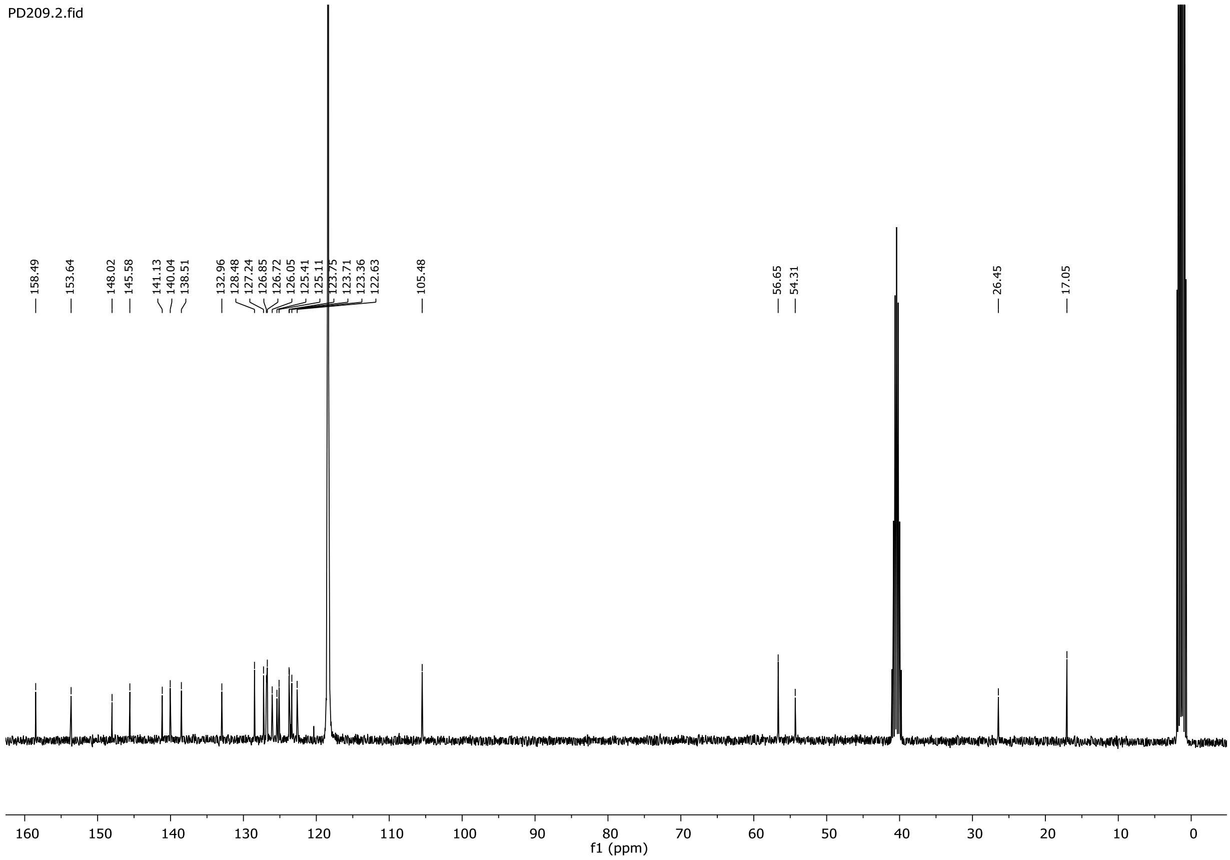
PD209.2.fid
f1 (ppm)
